# Proximity Engineering of Fe‒N_4_ Twins for Oriented Generation of Singlet Oxygen for Hospital Wastewater Treatment

**DOI:** 10.1002/anie.6249880

**Published:** 2026-02-06

**Authors:** Xinhao Wang, Zhaokun Xiong, Shuai Yang, Hongyu Zhou, Yanbiao Shi, Zelin Wu, Bingkun Huang, Lei Yang, Chuan‐Shu He, Xiaoguang Duan, Bo Lai

**Affiliations:** ^1^ State Key Laboratory of Hydraulics and Mountain River Engineering College of Architecture and Environment Sichuan University Chengdu China; ^2^ Sino‐German Centre for Water and Health Research Sichuan University Chengdu China; ^3^ School of Chemical Engineering The University of Adelaide Adelaide South Australia Australia

**Keywords:** electron spin state, Fe‒N_4_ twin sites, hospital wastewater treatment, selective singlet oxygen generation, volcano‐shaped Fenton‐like activity

## Abstract

Precisely tailoring the molecular configurations of single‐atom sites and elucidating their correlation with generated specific reactive species is crucial for advancing Fenton‐like chemistry toward targeted remediation. Herein, we developed a facile approach to precisely modulate the distances between isolated Fe‒N_4_ sites (d_Fe–Fe_) from nanometer (0.95 nm) to subnanometer (0.43 nm) to construct a family of well‐defined Fe‒N_4_ twins with manipulated ligand‐field strength and spin states. Different Fe‒N_4_ twin sites trigger a metal‐loading‐independent volcano‐shaped Fenton‐like activity trend. The optimal configuration, achieved at an Fe‒Fe distance of 0.43 nm (Fe_d0.43_SA), induces an intermediate‐spin (t_2g_4e_g_1) configuration that optimizes e_g_ orbital occupancy, thereby promoting peroxymonosulfate (PMS) adsorption to form *HSO_5_^−^ and subsequently lowers the energy barrier for coupling with another PMS to selectively generate singlet oxygen (^1^O_2_). The robust molecular catalyst with Fe‒N_4_ twin sites sustains over 120 h of continuous treatment of organic wastewater and demonstrates simultaneous disinfection and pharmaceutical removal of actual hospital wastewater. This work presents an advanced strategy for engineering single‐atom sites with multi‐site cooperativity to regulate Fenton‐like catalysis, enabling rapid and real‐world water purification.

## Graphical abstract

Precise modulation of Fe–N_4_ site distances constructs spin‐regulated Fe–N_4_ twins, triggering a volcano‐type Fenton‐like activity and promoting oriented singlet oxygen generation. The twin‐site catalyst enables highly selective and efficient hospital wastewater purification with simultaneous pharmaceutical degradation and disinfection under low peroxymonosulfate dosage.

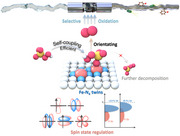

## 1 Introduction

Persistent organic pollutants (POPs) in aquatic environments pose a growing global challenge, with pharmaceutical contaminants and the associated risks of antibiotic resistance presenting serious threats to ecosystem stability and human health [1, 2]. Peroxymonosulfate (PMS)‐based advanced oxidation processes (AOPs) offer an effective strategy for water purification by generating reactive oxygen species (ROS) to degrade organic pollutants and inactive pathogens [3, 4]. Among these, free radicals, such as hydroxyl radicals (^•^OH, 1.9–2.7 V vs. NHE) and sulfate radicals (SO_4_^•−^, 2.5–3.1 V vs. NHE), are particularly attractive for their high oxidizing potential [5, 6]. However, their nonselective reactivity makes them vulnerable to background water constituents, inducing undesired side reactions, producing harmful disinfection byproducts, and consuming ROS, thereby lowering chemical utilization efficiency and significantly diminishing purification efficiency when treating complex water matrices [7, 8]. In contrast, singlet oxygen (^1^O_2_) has attracted increasing attention in AOP technologies due to the strong immunity to background interference and high selectivity toward organic contaminants [9, 10]. Furthermore, the singlet oxygen pathway enhances PMS utilization efficiency while minimizing toxic byproduct formation [11, 12]. Achieving oriented generation of ^1^O_2_ from PMS requires meticulously designed active sites with favorable geometric and electronic structures to synergistically regulate PMS adsorption configuration and activation pathways, thereby enabling the formation of key intermediates for ^1^O_2_ production.

The transition‐metal‐based single‐atom catalysts (TM‐SACs), featuring atomically dispersed metal sites, tailored coordination environment, and excellent stability, exhibit excellent PMS activation activity to generate diverse ROS [13, 14, 15]. Thus, TM‐SACs provide an ideal platform for elucidating the relationships between the microenvironment of active sites and catalytic behaviors. Our previous work revealed that converting active sites from nanoparticles to single atoms shifts the primary ROS from radicals to ^1^O_2_, which exhibits high selectivity toward electron‐rich contaminants in water [11]. Furthermore, designated CoN_4_ sites with weakly positive Co atom reduced the energy barriers for ^1^O_2_ production via facilitating one‐electron oxidation of PMS to yield SO_5_^•‒^ as the key intermediate [16]. Because of its moderate oxidation capacity (2.2 V vs. NHE) and high selectivity, ^1^O_2_ can effectively attack organic pollutants in complex water environments, thereby improving wastewater biodegradability and substantially reducing oxidant consumption [12].

Inspired by cooperative adsorption and co‐catalysis at adjacent active sites in natural enzyme systems, proximity engineering in SACs is a state‐of‐the‐art approach for precise regulation of catalytic behavior through electronic and geometric effects [17, 18]. For example, modulating the distance between adjacent Fe‒N_4_ sites altered the electronic structures of individual sites, markedly enhancing oxygen reduction activity when neighboring Fe atoms approached a separation of ∼0.7 nm [19]. Moreover, when copper‒copper (Cu‒Cu) atom distance (5–6 Å) geometrically matched to the peroxydisulfate (PDS) molecular size, the PDS adsorption and activation were greatly enhanced, promoting nonradical oxidation of contaminant via an interfacial electron‐transfer manner [17]. Although the substrate‐size‐matching strategy provides an effective approach for fine‐tuning SAC efficiency and selectivity, its applicability is limited by substrate specificity and precision of manipulation. Beyond geometric effects, the electronic configurations of metal centers, particularly their spin states, profoundly govern interactions and electron transfer with peroxides, thereby determining ROS selectivity. For example, introducing sulfur heteroatoms into the higher coordination shell of Fe‒N_4_ sites induced 3d orbital splitting and spin crossover, weakening Fe–O bonding and facilitating PMS‐derived O coupling, thereby achieving nearly 100% ^1^O_2_ selectivity in PMS activation [10]. Moreover, incorporating intrinsic carbon defects adjacent to Fe‒N_4_ sites shifted the Fe d‐band center via long‐range electronic interactions, optimizing orbital occupancy and antibonding states, thereby enhancing PMS activation and enabling selective formation of high‐valent Fe = O (O = Fe‒N_4_) [20]. Nevertheless, the intrinsic link between proximity‐induced electronic/spin‐state modulation and the selective formation of specific ROS (e.g., ^1^O_2_) remains poorly understood.

In this study, we fabricated a series of Fe‒SACs with well‐defined distances between adjacent Fe‒N_4_ sites, named Fe_dx_SA, where dx denotes the distance between adjacent Fe atoms (d_Fe‒Fe_). A hydrogel‐anchoring strategy was applied to regulate the distance of the Fe‒N_4_ site, while ensuring a high product yield. These proximity‐engineered Fe‒N_4_ twins enable precise regulation of geometric separation and modulated spin states to manipulate PMS activation pathways. Specifically, Fe‒N_4_ twin sites reorganize the d‐electron configuration and modify the ligand field of the Fe center, thereby inducing a spin‐state transition from a low‐spin state of individual Fe‒N_4_ site to an intermediate‐spin (t_2g_4e_g_1) configuration of the twin configuration. This moderately filled e_g_ orbital enhances the orbital coupling between the Fe centers and PMS, facilitating electron transfer from Fe to PMS and ultimately promoting selective ^1^O_2_ generation for phenol (PE) degradation. A suite of analytical experiments, including electron paramagnetic resonance (EPR), quenching experiments, and electrochemical analyses, demonstrated the dominant contribution of ^1^O_2_ in the Fe_dx_SA/PMS system. The amount of ^1^O_2_ production was also semi‐quantitatively assessed by chemical trapping test and quadrupole time‐of‐flight mass spectrometry (UPLC‐Q‐TOF‐MS/MS). In situ Raman and density functional theory (DFT) calculations provided further insights into selective ^1^O_2_ generation from PMS via atomic‐distance engineering. Moreover, the Fe_d0.43_SA/PMS system was employed to remove micropollutants and pathogenic microorganisms in real hospital wastewater, thereby reducing toxicity and antibiotic resistance.

## 2 Results and Discussions

### 2.1 Structural and Spin State Analysis of Fe_dx_SA

**FIGURE 1:**
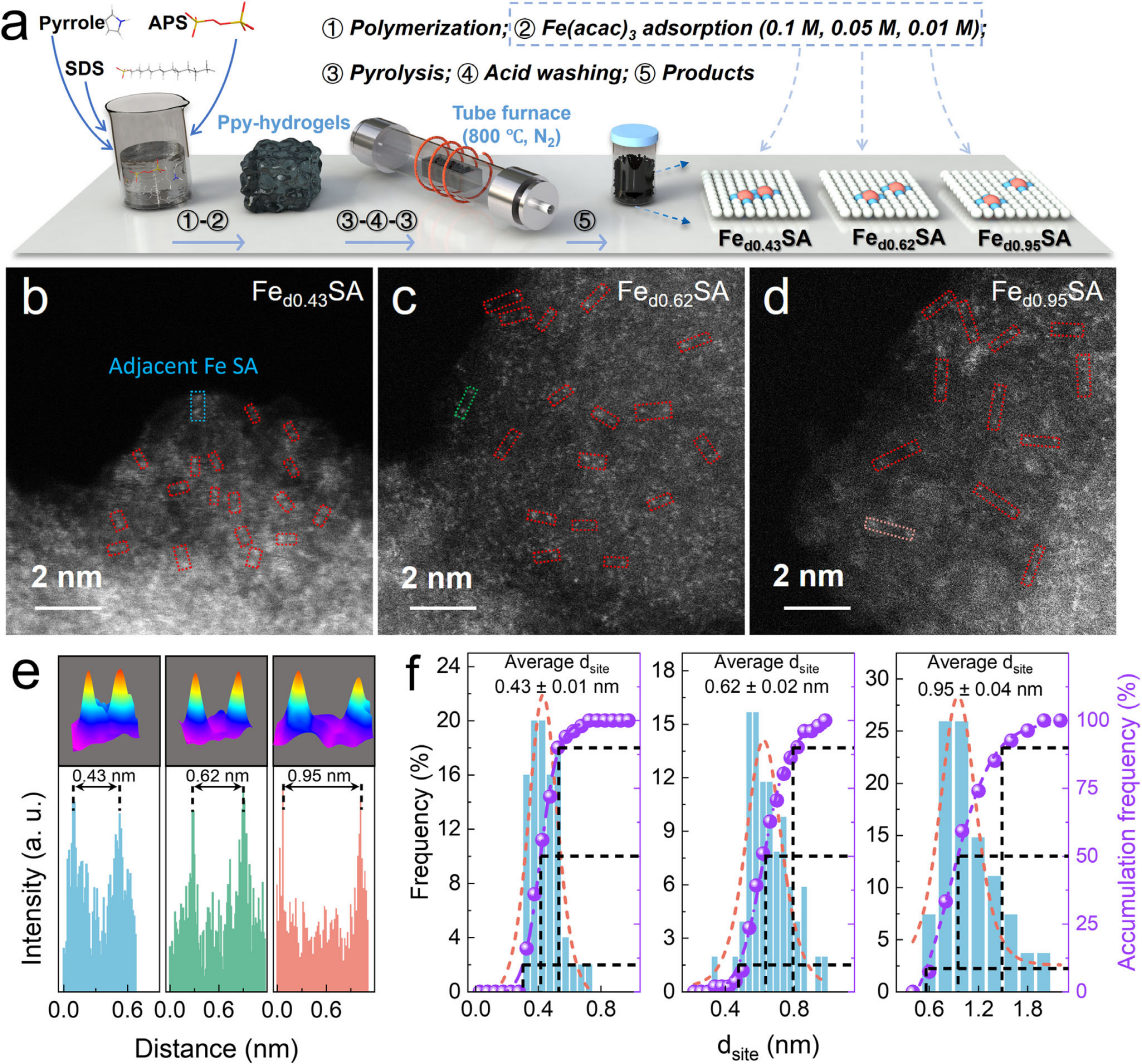
Regulation of the d_Fe‒Fe_ of Fe_dx_SA. (a) Schematic illustration of the synthesis approach of Fe_dx_SA. AC‐HAADF‐STEM images of (b) Fe_d0.43_SA, (c) Fe_d0.62_SA, and (d) Fe_d0.95_SA. (e) The intensity profile of adjacent Fe atoms (blue: Fe_d0.43_SA, green: Fe_d0.62_SA, and pink: Fe_d0.95_SA). (f) Statistical distribution of d_Fe‒Fe_ in three catalysts.

The synthesis processes of Fe_dx_SA are shown in Figure 1a (synthesis details were described in the Supporting Information) [19]. The regulation of the distance of Fe‒N_4_ twins was realized by modifying the concentration of the Fe(acac)_3_ precursor (CFe(acac)_3_). However, the excess Fe(acac)_3_ did not increase atomic Fe loading (Figure S1), likely due to the formation of metal nanoparticles, which were subsequently removed during acid washing [11]. The mass loading percentage was 1.39 wt% (Fe_d0.95_SA), 2.25 wt% (Fe_d0.62_SA), and 1.82 wt% (Fe_d0.43_SA). X‐ray diffraction (XRD) patterns (Figure S2) of Fe_dx_SA showed only two diffraction peaks assigned to the 002 (26°) and 101 (44°) planes of graphitic carbon, suggesting that Fe atoms were well‐dispersed without Fe clusters or nanoparticles [21].

Scanning electron microscope (SEM) images of CN, Fe_d0.43_SA, Fe_d0.62_SA, and Fe_d0.95_SA displayed similar irregular carbon spheres (Figure S3). High‐angle annular dark‐field scanning transmission electron microscopy (HAADF‐STEM) revealed that the Fe, C, and O were uniformly dispersed, which was also indicated by energy‐dispersive x‐ray spectroscopy (EDX) mapping analysis (Figures S4–S6). Furthermore, aberration‐corrected HAADF‐STEM (AC‐HAADF‐STEM) showed the fine dispersion of Fe atoms in Fe_dx_SA with well‐regulated distance of adjacent Fe‒N_4_ twins, as shown in Figures 1b–d and S7. Marked by dashed blue, green, and pink rectangles, d_Fe‒Fe_ of the adjacent Fe atoms was measured to be 0.43 nm (Fe_d0.43_SA), 0.62 nm (Fe_d0.62_SA), and 0.95 nm (Fe_d0.95_SA), respectively (Figure 1e). Then, around 100 atoms were collected from AC‐HAADF‐STEM images for each catalyst (histograms in Figure 1f) [19]. Statistical results showed that average twin distances (d_site, 50_) were 0.43 ± 0.01 nm (Fe_d0.43_SA), 0.62 ± 0.02 nm (Fe_d0.62_SA), and 0.95 ± 0.04 nm (Fe_d0.95_SA), which were consistent with the simulated twin models (Figures 1f and S8).

**FIGURE 2:**
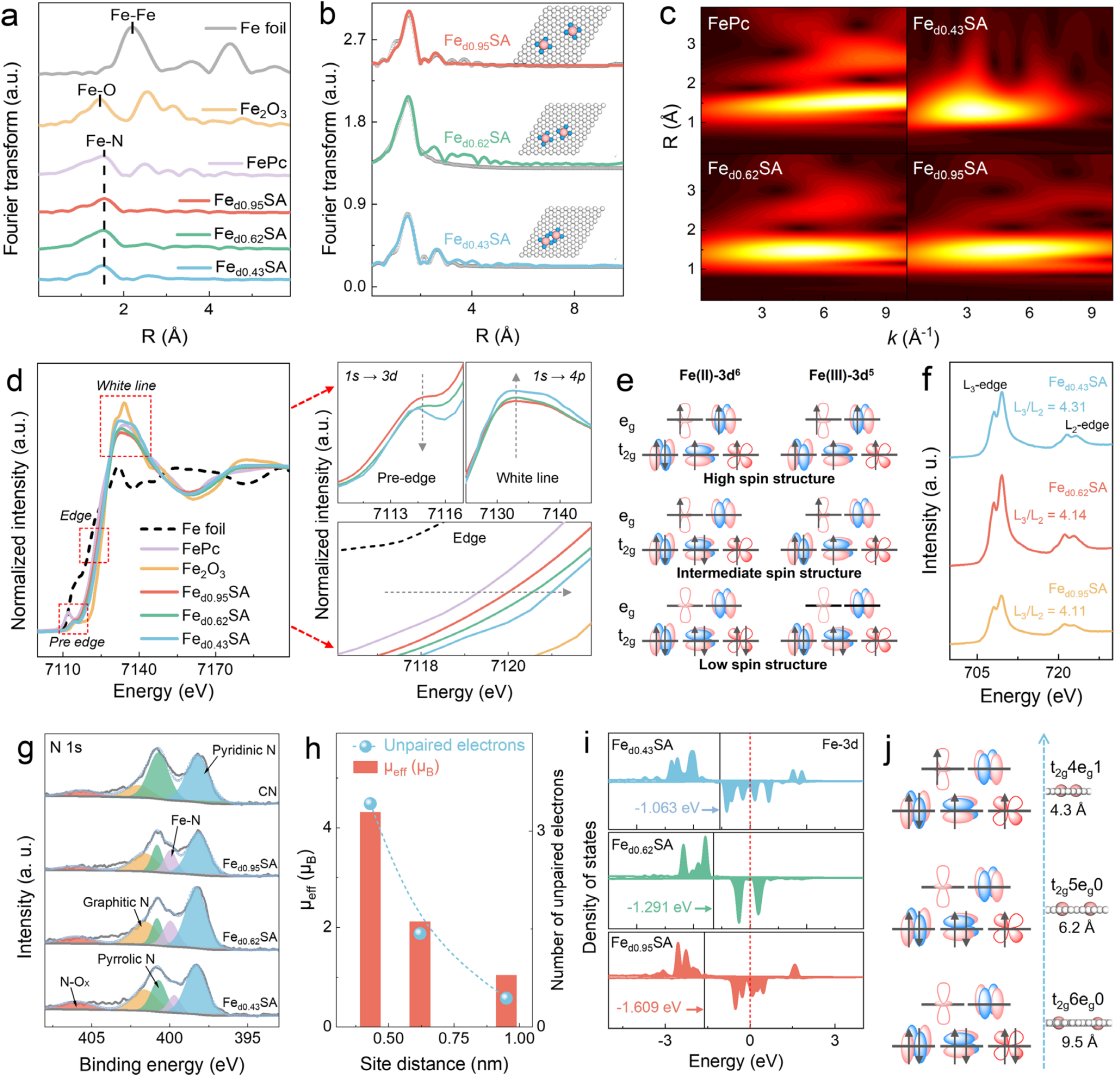
Atomic coordination and spin state analysis. (a) Fourier‐transform extended XAFS spectra. (b) EXAFS in R‐space and corresponding fitting curves for Fe_dx_SA. (c) Wavelet‐transformed images. (d) Fe K‐edge XANES spectra. (e) Schematic diagram of the d‐orbital splitting manner of high spin, intermediate spin, and low spin states for Fe(II) or Fe(III). (f) Fe L‐edge XANES spectra. (g) XPS spectrum of N 1s. (h) Calculated *µ*_eff_ values and the number of unpaired electrons for Fe_dx_SA. (i) Fe 3d DOS plots of Fe_dx_SA. (j) The relationship between d_Fe‒Fe_ and spin states.

X‐ray absorption fine structure spectroscopy (XAFS) was carried out to further confirm the chemical state and coordination environment of Fe atoms loaded onto Fe_dx_SA [22]. The Fourier‐transform extended XAFS (FT‐EXAFS) spectra of Fe_d0.43_SA, Fe_d0.62_SA, and Fe_d0.95_SA had a peak for metal‐N*_x_* at about 1.53 Å resembling the FePc reference, which was similar to the Fe‒N_4_ but significantly different from the Fe─Fe bond (Fe foil) at 2.2 Å and Fe─O bond (Fe_2_O_3_) at 1.3 Å (Figure 2a) [19]. The coordination number of the first shell of Fe in Fe_dx_SA was close to 4 (Figures 2b, S9, and Table S1). Furthermore, only one maximum intensity at ∼ 4 Å^−1^ in wavelet‐transformed (WT) images also confirms the Fe‒N coordination in the first shell (Figure 2c) but is significantly different from the bond of Fe─Fe or Fe─O (Figure S10) [20].

Besides, the relations among CFe(acac)_3_, Fe loading, and d_Fe‒Fe_ were further investigated. Figure S11a demonstrates that Fe loading increased from 1.39 to 4.2 wt% when CFe(acac)_3_ increased from 0.01 to 0.075 M. However, when CFe(acac)_3_ reached 0.1 M, Fe loading dramatically declined to 1.82 wt%. The result suggested that an excessive dose of CFe(acac)_3_ facilitated the aggregation of metal atoms into clusters or nanoparticles, which were subsequently removed by acid washing (Figure S11b). The above results confirm that the Fe species were atomically dispersed as Fe‒N_4_ in Fe_dx_SA, with the d_Fe‒Fe_ spacing precisely regulated across three Fe‒N4 twin catalysts.

Figure 2d presents the Fe K‐edge x‐ray absorption near‐edge structure (XANES) spectra of three Fe‒N_4_ SACs with distinct inter‐site distances. In the pre‐edge region, the absorption intensity decreases with reducing d_Fe‒Fe_, with all samples exhibiting intensities intermediate between FePc and Fe_2_O_3_. This trend suggests that the decreased twin distance enhances electronic coupling between metal centers, thereby attenuating the 1s → 3d transition [23, 24]. Notably, the most distantly isolated Fe_d0.95_SA exhibits a pre‐edge profile more akin to that of FePc, whereas Fe_d0.43_SA, containing the closest‐paired Fe‒N_4_ sites, shows a pre‐edge profile resembling that of Fe_2_O_3_. In the edge region, the absorption energies of all samples lie between Fe foil and Fe_2_O_3_ and progressively shift to higher energies with decreasing d_Fe‒Fe_, indicative of an increase in the average oxidation state and a concomitant reduction in d‐electron count [25].

Furthermore, analysis of the white‐line region reveals that as the d_Fe‒Fe_ decreases, the white‐line peak becomes both more intense and broader. This not only reflects an elevated oxidation state of Fe, but also indicates a spin‐state transition [24]. Specifically, as d_Fe‒Fe_ decreased, low‐spin (LS) Fe species of weakly interacted Fe‒N_4_ sites in lower oxidation states transitioned to a mixed‐spin configuration of the twin sites with greater high‐spin (HS) contribution in higher oxidation states, resulting in an overall intermediate‐spin (IS) character (Figure 2d,e). A positive relationship was previously revealed between the area ratios of L_3_/L_2_ and the spin state [26]. Figure 2f shows the Fe L_3_/L_2_ ratio in Fe L‐edge XANES spectra increases from 4.11 (Fe_d0.95_SA) to 4.14 (Fe_d0.62_SA) and reaches 4.31 for Fe_d0.43_SA, indicating a progressive increase in Fe spin state as the d_Fe–Fe_ decreases. High‐resolution N 1s spectra indicate that isolated Fe‒N_4_ sites are primarily coordinated by pyridinic nitrogen, whereas the Fe‒N_4_ twins are accompanied by an increased proportion of pyrrolic nitrogen as the metal atoms get closer (Fe_d0.43_SA) (Figure 2g and Table S2). This coordination‐chemistry shift indicates a transition from a strong‐ to a weak‐ligand‐field environment, consistent with the observed spin‐state alteration.

Magnetic measurements, obtained via temperature‐dependent zero field cooling and field cooling procedure (ZFC/FC) magnetization following the Curie‒Weiss law, reveal effective magnetic moments (*µ*_eff_) of 4.31, 2.12, and 1.04 *µ*_B_ for Fe_d0.43_SA, Fe_d0.62_SA, and Fe_d0.95_SA, corresponding to average numbers of unpaired electrons are calculated to be approximately 3.42, 1.43, and 0.44 (Figures 2h and S12), respectively [26, 27]. Moreover, ^57^Fe Mössbauer spectroscopy of Fe_dx_SA showed only doublet signals without singlet or sextet components, excluding the existence of iron crystalline species, consistent with XAS and AC‐HAADF‐STEM results (Figure S13). Quantitative analyses further revealed that the dominant components corresponding to Fe_d0.43_SA, Fe_d0.62_SA, and Fe_d0.95_SA were doublet 1 (D1, IS Fe(III), 62%), doublet 2 (D2, LS Fe(III), 46%), and doublet 3 (D3, LS Fe(II), 53%), respectively (Table S3) [10, 28, 29]. Density of states (DOS) analyses further reveal that as the d_Fe‒Fe_ decreases from 0.95 to 0.43 nm, the d‐band (ɛ_d_) center shifts upward from ‒1.609 to ‒1.063 eV, and the gap between the ɛ_d_ and the Fermi level (E_F_) narrows (Figures 2i and S14), thereby enhancing reactant binding capacity and facilitating electron transfer processes. This shift indicates increased d‐electron occupation, particularly within e_g_ orbitals [30, 31]. Projected density of states (PDOS) further confirms enhanced filling of e_g_ states and reduction in t_2g_ state occupancy near the E_F_ (Figure S15), suggesting a reduction in the crystal field splitting energy, thereby favoring the transformation to the mixed‐spin configuration [32]. Hence, the above discussion demonstrates that Fe_d0.43_SA predominantly exhibits a mixed IS state, while Fe_d0.62_SA and Fe_d0.95_SA are in dominant LS states as Fe(III)‐3d^5^ and Fe(II)‐3d^6^, respectively (Figure 2j).

### 2.2 Fe‒N_4_ Twins Dependent Catalytic Activity and Mechanism of Fe_dx_SA

**FIGURE 3:**
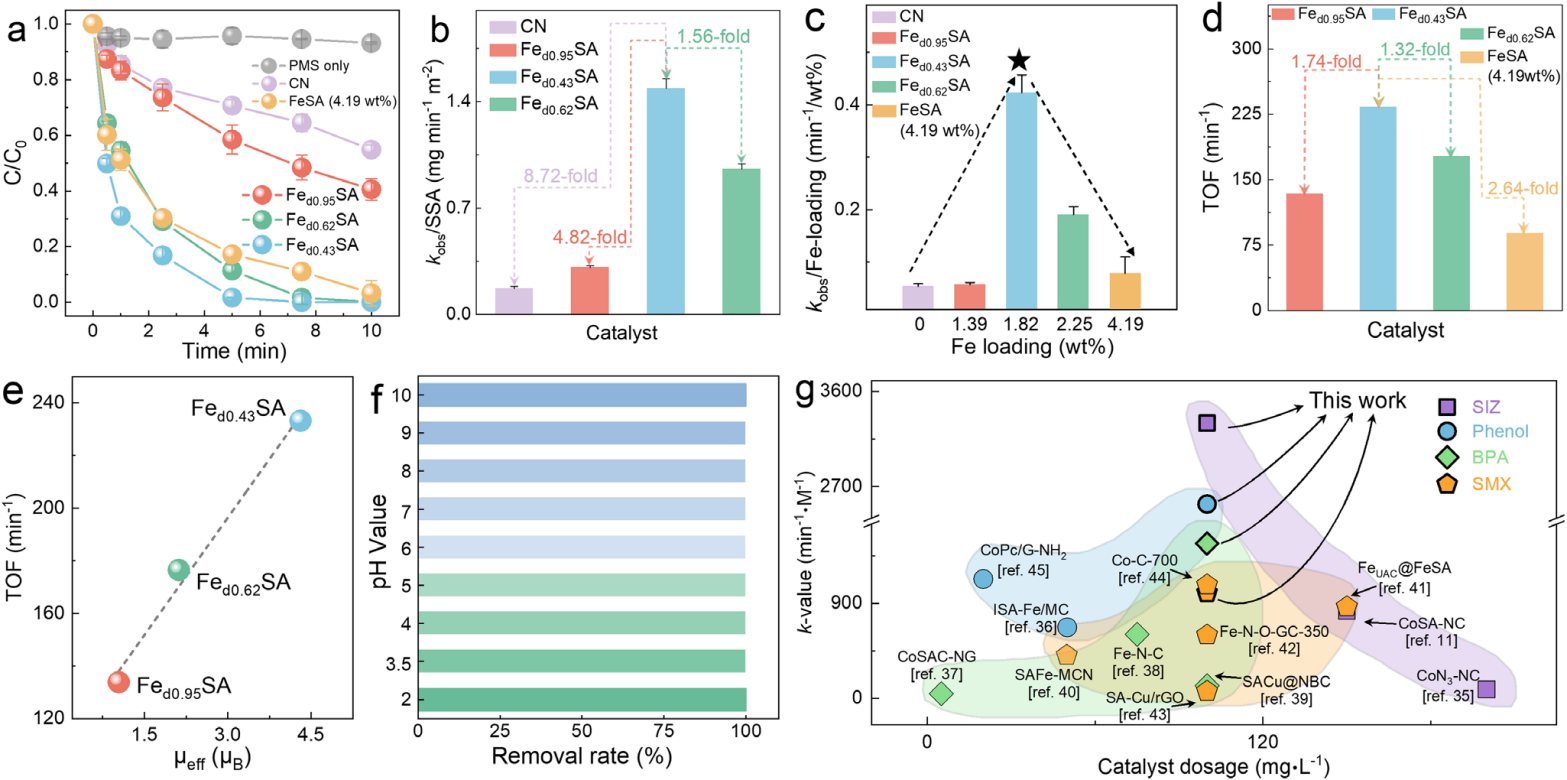
Catalytic performance of Fe_dx_SA in PMS‐based Fenton‐like reaction. (a) The PE degradation in different systems. Comparison of (b) *k*_obs_/SSA, (c) *k*_obs_/Fe‐loading, and (d) TOF for Fe_dx_SA catalysts. (e) Relation between the intrinsic reactivity of Fe_dx_SA and *µ*_eff_ values. (f) The effects of a wide pH range (2–10) on PE removal in Fe_d0.43_SA/PMS. (g) Comparison of the kinetics of organic contaminants removal by TM‐N‐C SACs for activation of PMS systems [11, 35, 36, 37, 38, 39, 40, 41, 42, 43, 44, 45]. Experimental conditions: [catalyst]_0_ = 100 mg L^−1^, [PMS]_0_ = 0.3 mM, [PE]_0_ = 40 µM. Error bars are the standard error values of three tests (*n* = 3).

The Fenton‐like performance of Fe_dx_SA was evaluated for PMS activation to oxidize phenolic pollutants in water. Under the optimal reaction conditions (Figure S16), PMS alone cannot degrade PE, and approximately 40% of PE was adsorbed by Fe_d0.43_SA (Figures 3a and S17). However, Fe_d0.62_SA and Fe_d0.95_SA only attained 20% PE adsorption, similar to the CN system. The different adsorption capacities were attributed to the specific surface area (SSA) of these catalysts; Fe_d0.43_SA exhibited the highest SSA (517 m^2^ g^−1^), followed by Fe_d0.62_SA (450 m^2^ g^−1^), Fe_d0.95_SA (279 m^2^ g^−1^), and CN (317 m^2^ g^−1^) (Figure S18). Changes in the ratio of *I*_D_ (1343 cm^−1^) to *I*_G_ (1574 cm^−1^) in the Raman spectra indicated that the graphitization degree declined. The defects/disorders observed with the decrease in d_Fe‒Fe_ are due to increased metal loading, which intensifies graphitization of the catalyst (Figure S19) [33]. With the addition of PMS, Fe_d0.43_SA possessed the highest catalytic activity among the prepared catalysts, achieving complete PE removal within 5 min and 100% PMS utilization (Figures 3a and S20a). The observed rate constant (*k*_obs_) of the Fe_d0.43_SA/PMS system (0.77 min^−1^) was approximately 14 times higher than the CN/PMS system (0.08 min^−1^) (Figure S20b).

Furthermore, normalized *k*_obs_/SSA and *k*_obs_/Fe‐loading values were employed to evaluate the intrinsic activity [34]. The *k*_obs_/SSA value for Fe_d0.43_SA is 1.48 mg min^−1^ m^−2^, which is 8.72‐, 4.82‐, and 1.56‐fold that of CN, Fe_d0.95_SA, and Fe_d0.62_SA, respectively, further indicating the promoting effect of the optimal Fe‒Fe distance (Figure 3b). The impact of Fe loading on intrinsic catalytic activity was assessed. Figure 3c showed a volcano‐shaped trend for *k*_obs_/Fe loading, where moderate Fe contents enhanced the activity, while excessive Fe loading resulted in a decrease in *k*_obs_/Fe loading. However, Fe_d0.43_SA/PMS with 1.82 wt% Fe loading still maintained the highest *k*_obs_/Fe loading value at 0.42 min^−1^/wt%. Therefore, Fe_d0.43_SA with Fe‒N_4_ twin sites exhibited higher intrinsic activity than isolated or more closely packed Fe‒N_4_ sites. To further evaluate the intrinsic reaction rate of per metal center, turnover frequency (TOF) of Fe_dx_SA was calculated (Figure 3d). Notably, Fe_d0.43_SA (with a lower metal loading) exhibited a much higher TOF (233.25 min^−1^) than Fe_d0.62_SA (176.47 min^−1^) and FeSA (4.19 wt%, 88.50 min^−1^). Furthermore, Figure 3e shows that the TOF value of Fe_dx_SA increases with the spin state transformation from LS (Fe_d0.95_SA) to IS (Fe_d0.43_SA) as the d_Fe‒Fe_ distance decreases. Thus, engineered Fe‒N_4_ twin sites with well‐defined proximity exhibited much higher intrinsic PMS activation activity than other coordination, irrespective of metal loading.

Moreover, the efficiency of the Fe_d0.43_SA/PMS system is pH‐independent, achieving 100% PE degradation within 10 min at pH 2.0–10.0 with ultralow iron leaching (Figures 3f and S21). Figure 3g and Table S4 show that Fe_d0.43_SA demonstrates the highest *k*‐value among state‐of‐the‐art M‐SACs (M for different metals) for oxidation of PE, sulfisoxazole (SIZ), bisphenol A (BPA), and sulfamethoxazole (SMX).

**FIGURE 4:**
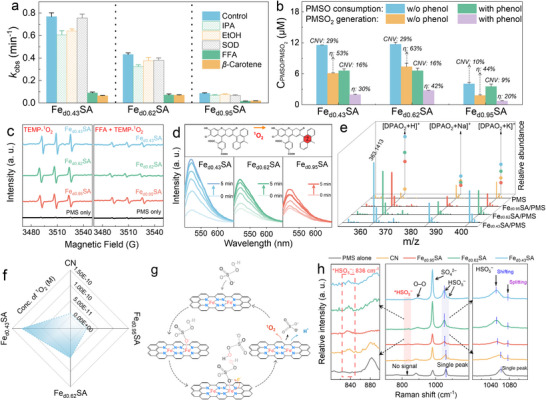
Spin‐state engineering via Fe‒N_4_ proximity regulates ROS generation in PMS‐based Fenton‐like reactions. (a) Comparison of kinetic constants under different quenching conditions in Fe_dx_SA/PMS systems. Experimental conditions: [catalyst]_0_ = 100 mg L^−1^, [PMS]_0_ = 0.3 mM, [PE]_0_ = 40 µM, [IPA]_0_ = [IPA]_0_ = [EtOH]_0_ = 300 mM, [FFA]_0_ = 10 mM, [*p*‐BQ]_0_ = 5 mM, and [β‐carotene]_0_ = 0.1 mM. (b) PMSO consumption (the conversion efficiency for PMSO in the figure is labeled as CNV) and PMSO_2_ production in Fe_dx_SA/PMS systems with/without PE. (c) EPR spectra in Fe_dx_SA/PMS systems with TEMP as the trapping agent of ^1^O_2_. (d) Fluorescence spectra of SOSG‐EN in Fe_dx_SA/PMS systems. (e) UPLC‐Q‐TOF‐MS/MS chromatograms for DPAO_2_ ([DPAO_2_ + H]^+^, [DPAO_2_ + Na]^+^, and [DPAO_2_ + K]^+^). (f) Comparison of the concentration of ^1^O_2_ for different catalyst systems. (g) The schematic diagram of the formation route of ^1^O_2_. (h) In situ Raman spectra in Fe_dx_SA/PMS systems. Error bars are the standard error values of three tests (*n* = 3).

To clarify the major ROS in Fe_dx_SA systems and analyze the influence of d_Fe‒Fe_ on ROS generation, the possible ROS species (e.g., ^•^OH, SO_4_^•−^, O_2_^•−^, and ^1^O_2_) were first investigated by selective quenching experiments. As shown in Figures 4a and S22, methanol (MeOH), ethanol (EtOH), *tert*‐butyl alcohol (TBA), and isopropanol (IPA), as scavengers for ^•^OH and SO_4_^•−^, all exhibited negligible inhibition on PE removal [46]. Moreover, EPR spectra and coumarin probe experiment failed to detect the characteristic signals for radical species in Fe_dx_SA/PMS systems (Figure S23), excluding the generation of ^•^OH and SO_4_^•−^. The seven peaks signal for DMPO‐X (αN = 7.3 ± 0.1 G and αH = 3.9 ± 0.1 G) was observed in Fe_d0.43_SA/PMS, suggesting the presence of nonradical species, such as surface complexes with much longer lifetimes, that could directly mediate electron transfer between PMS and the pollutant [47, 48]. However, Figure S24 shows that PE removal efficiency was significantly reduced by prolonging the premix time, suggesting that the PMS complex might transform into other ROS rather than remaining on the catalyst surface. The open‐circuit potentials showed that PMS addition sharply increased the potential, indicating the formation of a PMS complex that elevated the surface potential of Fe_dx_SA (Figure S25). However, the potential did not drop sharply with PE addition, remaining stable over time, ruling out catalyst‐shuttled electron transfer.

High‐valent iron‐oxo species have been extensively reported in the SAC/PMS system [49]. Methyl phenyl sulfoxide (PMSO) is used as a selective probe for high‐valent iron‐oxo species. Figures 4b and S26 show that, for PMS systems catalyzed by Fe_d0.43_SA, Fe_d0.62_SA, and Fe_d0.95_SA, the corresponding PMSO conversion efficiencies were 29%, 29%, and 10%, while the methyl phenyl sulfone selectivity (*η*_PMSO2_) was only 53%, 63%, and 44%, respectively. The PE addition further reduced both PMSO conversion efficiency and *η*_PMSO2_. Besides, the negligible formation of ^18^O‐isotope‐labeled methyl phenyl sulfone (PMS^16^O^18^O) in the ^18^O‐isotope‐labeling experiment excluded the formation of high‐valent iron‐oxo (Figure S27a–f). The conclusion is further supported by the negligible inhibitory effect of PMSO addition on PE removal, indicating that high‐valent iron‐oxo species play an insignificant role in PE oxidation (Figure S27g–i).

Furthermore, furfuryl alcohol (FFA) and superoxide dismutase (SOD), which can scavenge ^1^O_2_ (*k*_FFA_, ^1^O_2_ = 1.2 × 10^8^ M^−1^ s^−1^) and O_2_^•−^ (*k*_SOD_, O_2_^•−^ = 2.0 × 10^9^ M^−1^ s^−1^), were introduced in the Fe_dx_SA/PMS systems to assess the contribution of ^1^O_2_ and O_2_^•−^ [50, 51]. With the addition of FFA (Figures 4a and S28), *k*_obs_ of PE degradation was suppressed markedly from 0.77 to 0.09 min^−1^ in the Fe_d0.43_SA/PMS/PE system. Additionally, the β‐carotene as a probe for ^1^O_2_ (*k* ^1^O_2, β‐carotene_ = 2.0–3.0 × 10^10^ M^−1^ s^−1^) exhibited a similar inhibitory effect to FFA (Figures 4a and S29,30) [52]. Furthermore, the EPR spectra displayed triplet peak signals with a strength ratio of 1:1:1 (α^N^ = 17.24 G) of TEMP‐^1^O_2_ (Figure 4c), and the intensity of the peaks increased with the reduced Fe‐twin distance. The intensity of the TEMP‐^1^O_2_ signal declined substantially with the addition of FFA in all three systems. Furthermore, no characteristic EPR signals of O_2_^•‒^ were detected in all Fe_dx_SA/PMS systems, and the addition of SOD as the O_2_^•‒^ scavenger had minimal impact on PE removal, indicating that O_2_^•‒^ played a negligible role in the process (Figures 4a and S31,S32). Collectively, ^1^O_2_ serves as the primary reactive species in Fe_dx_SA/PMS systems.

### 2.3 Origin of Fe‒N_4_ Twins‐Dependent ^1^O_2_ Selectivity

The singlet oxygen sensor green (SOSG) was used for semi‐quantitative analysis of ^1^O_2_ by tracking the characteristic oxidation product of SOSG, the endoperoxide (SOSG‐EN) [53, 54]. A weak fluorescence signal at 525 nm was observed for sole PMS, with minimal SOSG‐EN accumulation over time (Figure S33a), indicating slow ^1^O_2_ production from PMS self‐decomposition. The CN/PMS system showed only a slight signal increase with limited ^1^O_2_ generation capacity (Figure S33b). When the Fe‒N_4_ twin catalysts were used, the signal of SOSG‐EN significantly increased with decreased d_Fe‒Fe_ (Figure 4d). Besides, the degradation rate of PE dramatically declined in Fe_dx_SA/PMS with the addition of KSCN to poison the metal sites, while CN/PMS was only slightly affected (Figure S34). This result certified the critical role of Fe‒N_4_ sites in PMS activation to selectively yield ^1^O_2_. Moreover, ^1^O_2_ can oxidize 9,10‐diphenylanthraquinone dyes (DPA) to form DPA endoperoxide (DPAO_2_), as detected by UPLC‐Q‐TOF‐MS/MS [55]. Figure 4e showed that the peaks of DPAO_2_ ([DPAO_2_ + H]^+^, [DPAO_2_ + Na]^+^, and [DPAO_2_ + K]^+^) were observed in Fe_dx_SA/PMS systems. The relative abundance of DPAO_2_ followed Fe_d0.43_SA/PMS > Fe_d0.62_SA/PMS > Fe_d0.95_SA/PMS > PMS. The steady‐state concentration of ^1^O_2_ ([^1^O_2_]_ss_) was 1.2 × 10^−10^ M in the Fe_d0.43_SA/PMS system, which was about 14‐, 9‐, and 2‐fold higher than CN/PMS, Fe_d0.95_SA/PMS, and Fe_d0.62_SA/PMS, respectively (Figures 4f and S35a,b). Moreover, due to the longer lifetime of ^1^O_2_ in deuterium oxide (D_2_O, 50 µs) compared to H_2_O (4 µs), Fe_dx_SA exhibited faster PE degradation in D_2_O (Figure S35c,d), further confirming the strong selectivity toward ^1^O_2_ generation [56]. Overall, these results illustrated the selective generation of ^1^O_2_ in the Fe_dx_SA/PMS systems with precisely regulated d_Fe‒Fe_.

The oxygen source of ^1^O_2_ was investigated as it might come from O_2_ (dissolved oxygen), H_2_O, or PMS. Purging the solution with N_2_ (reducing the oxygen level from 9.1 to 1.4 mg L^−1^) did not affect performance (Figures S36 and S37), while replacing water with methanol as the reaction medium also showed limited inhibition (Figure S38), indicating that ^1^O_2_ stemmed from PMS. As shown in Figure S39, increasing the concentration of sodium nitrite (NaNO_2_, a scavenger of SO_5_^•−^) from 1 to 5 mM did not suppress PE removal, indicating that ^1^O_2_ was not generated through rapid self‐coupling of SO_5_^•−^, supported by the monitored current transfer direction from catalysts to PMS, which would not generate SO_5_^•−^ (Figure S40) [57]. Thus, ^1^O_2_ generation is proposed to proceed through direct coupling of surface‐activated PMS with a surrounding PMS via *HSO_5_^‒^ + HSO_5_^‒^→^1^O_2_ + 2H^+^ + 2SO_4_^2‒^ (Figure 4g) [57, 58]. This is further supported by the slowly yet continuously decreased solution pH when the catalyst was introduced into the PMS solution (Figure S41). The formation of surface PMS complex was analyzed by in situ Raman spectra (Figure 4h). The peaks at 1062, 982, and 882 cm^−1^ were assigned to HSO_5_^‒^ and SO_4_^2‒^ components as well as the peroxyl O─O bond in PMS, respectively [26]. Compared to the sole PMS system, a new peak (836 cm^−1^) appeared in Fe_dx_SA/PMS systems, attributed to the metastable metal‐PMS complex (*PMS) [14, 26]. Compatibly, the maximum value of ISO_4_^2‒^/IHSO_5_^‒^ was observed in the Fe_d0.43_SA/PMS system, and the value decreased with the increase of d_Fe‒Fe_ (Table S5), demonstrating that the closer distance of Fe‒N_4_ twins facilitated optimal PMS adsorption and decomposition to SO_4_^2‒^. Moreover, the peak of HSO_5_^‒^ was split into two peaks in Fe_dx_SA/PMS systems, and the peak center gradually blue shifted with the decrease in d_Fe‒Fe_, moving to 1052 cm^−1^ in the presence of Fe_d0.43_SA, compared with 1062 cm^−1^ for free PMS and 1060 for CN (Figure 4h and Table S5), indicating the strong electronic interactions between PMS and the Fe center [14].

**FIGURE 5:**
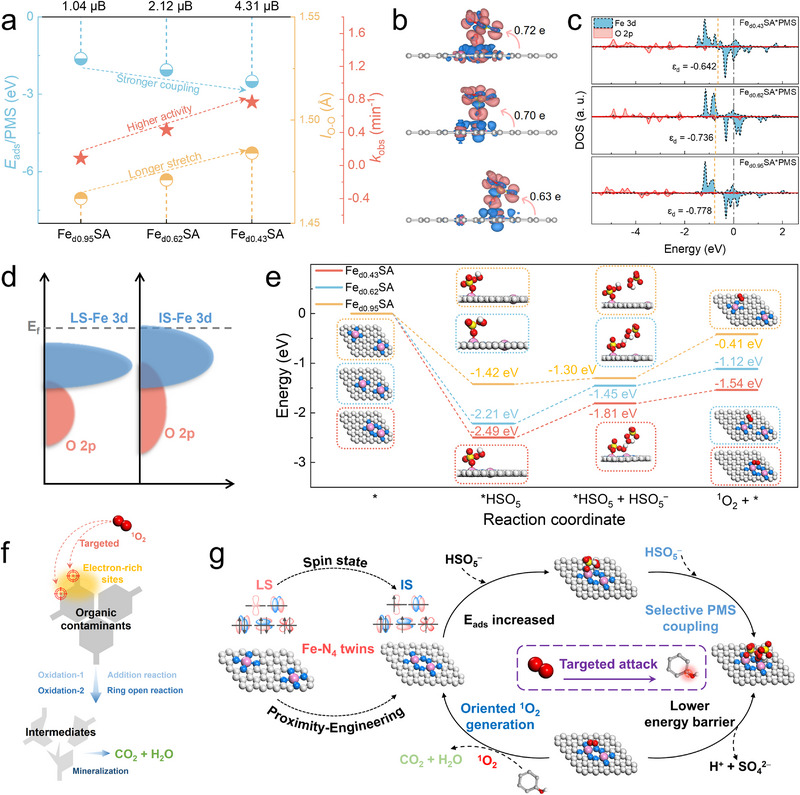
Unraveling the mechanism of IS Fe‒N_4_ twin sites on selective ^1^O_2_ generation and targeting contaminants oxidation. (a) Correlation among the coupling strength to PMS, the stretching length of O‒O bind in PMS, the oxidation capacity, and the *µ*_eff_ of Fe_dx_SA. (b) The charge comparison of density difference and Bader charge of Fe_d0.43_SA (top), Fe_d0.62_SA (middle), and Fe_d0.95_SA (bottom) (blue and red represent the dissipation and aggregation of electrons, respectively). (c) PDOS of Fe 3d and O 2p after adsorbing PMS on Fe‒N_4_ in different Fe_dx_SA systems. (d) Illustration for the upshifted d‐band center from LS to IS. (e) Reaction pathways and the energy profile diagram of PMS dissociation and ^1^O_2_ generation on Fe_dx_SA (insert: corresponding intermediate configuration). (f) Proposed mechanism of ^1^O_2_ selective oxidation toward electron‐rich contaminants. (g) The proposed mechanism for the effects of Fe‒N_4_ twin sites on PMS activation.

Furthermore, computations show that the introduction of adjacent Fe‒N_4_ twin sites substantially enhances PMS adsorption, as supported by adsorption energy (*E*_ads_) calculations (Figures S42–S46). Notably, Fe_d0.43_SA, featuring the IS state, exhibits a much stronger PMS adsorption affinity (‒2.45 eV) than LS‐based Fe_d0.62_SA (‒2.05 eV) and Fe_d0.95_SA (‒1.62 eV), attributed to its unique t_2g_4e_g_1 electronic configuration. In this configuration, the unpaired electron in the higher‐energy e_g_ orbital facilitates more efficient electron transfer, while the partially vacant e_g_ orbital strengthens PMS binding via optimized orbital overlap [59]. As evidenced in Figure 5a,b, the bond length of peroxyl O─O bond in Fe_d0.43_SA (1.48 Å) is extended compared to Fe_d0.62_SA (1.47 Å) and Fe_d0.95_SA (1.46 Å), and is accompanied with a greater Bader charge transfer from Fe‒N_4_ to PMS in Fe_d0.43_SA (0.72 e). The electrochemical impedance spectra and Tafel polarization diagram further supported the superior charge‐transfer capacity of Fe_d0.43_SA (Figure S46b,c) [56]. This collectively corroborates the enhanced PMS activation of Fe‒N_4_ twin sites with the IS configuration. Moreover, the shortened O─H bond in adsorbed HSO_5_^‒^ suggests stronger proton binding compared to free PMS, making direct deprotonation less favorable (Figure S46d). This thermodynamic constraint promotes the selective *HSO_5_^‒^ coupling with a free HSO_5_^‒^ to generate ^1^O_2_ [58].

The proximity‐engineered Fe‒N_4_ twin sites narrow ɛ_d_ of Fe atoms after PMS adsorption, and the optimized ɛ_d_ for Fe_d0.43_SA, Fe_d0.62_SA, and Fe_d0.95_SA are ‒0.64, ‒0.74, and ‒0.78 eV (Figure 5c). According to Hammer and Nørskov, ɛ_d_ closer to E_F_ leads to reduced occupation of d–σ* antibonding states, thereby facilitating stronger substrate–metal interactions [60]. For Fe_d0.43_SA, the half‐filled dz^2^ orbital overlaps effectively with the 𝜋* orbital of PMS, forming a single occupied dz^2^–𝜋^*^ bonding state (Figure S47). This orbital interaction induces a synergistic σ‐donation/π‐back‐donation process that partially populates the O‒O antibonding orbital, thereby activating while stabilizing the surface‐confined PMS (*HSO_5_^‒^). In this configuration, the Fe─O bond is strengthened for HSO_5_^‒^ adsorption while the O─O bond is weakened but not completely cleaved, preventing homolytic rupture into SO_4_^•‒^/^•^OH while providing sufficient electronic density to promote bimolecular coupling between PMS* and free PMS, ultimately yielding ^1^O_2_ [10, 45]. As revealed by the projected crystal orbital Hamilton population (pCOHP) analysis (Figure S48), the IS state enhances Fe–O bonding, as evidenced by a higher integrated pCOHP (IpCOHP) value (0.026), confirming the stabilization of PMS*. Therefore, with a moderate degree of e_g_ filling and adsorption capacity, the IS (t_2g_4e_g_1) configuration of Fe centers in Fe‒N_4_ twin sites optimizes d‐electron orbital interactions, accelerates electron transfer into the PMS O‒O antibonding orbital, and steers PMS activation toward selective ^1^O_2_ generation rather than radical pathways (Figure 5d).

Reaction pathways and the energy profile diagram of PMS dissociation and step‐wise ^1^O_2_ generation on Fe_dx_SA were calculated in Figure 5e. The energy profiles demonstrated that a more negative Gibbs free energy in Fe_d0.43_SA/PMS system among the three systems in every step, and the free energy of *^1^O_2_ followed the sequence of Fe_d0.43_SA/PMS (−1.54 eV) < Fe_d0.62_SA/PMS (−1.12 eV) < Fe_d0.95_SA/PMS (−0.41 eV). The result indicates that the thermodynamic feasibility for selective ^1^O_2_ generation is most favorable at the Fe‒N_4_ twin sites, with a distance of 0.43 nm, matching experimental results. Besides, when the second PMS molecule was adsorbed, the O─H bond of PMS molecules was stretched toward ^1^O_2_ generation and release of H^+^ and SO_4_^2‒^ (Figure S49) [57, 58].

Electrostatic potential (ESP) analysis revealed that electron‐rich contaminants (e.g., SIZ, PE, BPA, SMX, and acyclovir (ACV)) possess higher ESP values and exhibit higher degradation efficiencies in the Fe_d0.43_SA system, compared with electron‐deficient aromatics (nitrobenzene (NB), benzoic acid (BA), and atrazine (ATZ)) (Figure S50 and Table S6) [51]. Such selectivity is consistent with the benchmark ^1^O_2_ system of HClO/H_2_O_2_ (Figure S51) [61]. The preferential reactivity toward electron‐rich organics arises from their smaller HOMO–LUMO gaps relative to ^1^O_2_ (Figure S52), which reduces the electron‐transfer barrier from the pollutant's HOMO to the LUMO of ^1^O_2_ [62]. In addition, UPLC‐Q‐TOF‐MS/MS analyses revealed that ^1^O_2_ preferentially attacks phenolic hydroxyls, amino groups, and their ortho/para positions of the pollutants (Figures S53, S54 and Table S7), indicating that the dominant pathway involves electrophilic addition of ^1^O_2_, followed by ring‐opening and subsequent mineralization into small molecules (Figure 5f).

Overall, the proximity of adjacent Fe‒N_4_ twin sites induces a spin‐state transition of Fe 3d orbitals from LS to IS, enhancing PMS adsorption and accelerating electron transfer into the O‒O antibonding orbital of PMS. The electron injection stabilizes *PMS and prohibits direct O‒O homolysis, facilitating bimolecular PMS coupling to yield ^1^O_2_ for selective degradation of electron‐rich contaminants (Figure 5g).

### 2.4 Potential Practicability of Decontamination and Disinfection

**FIGURE 6:**
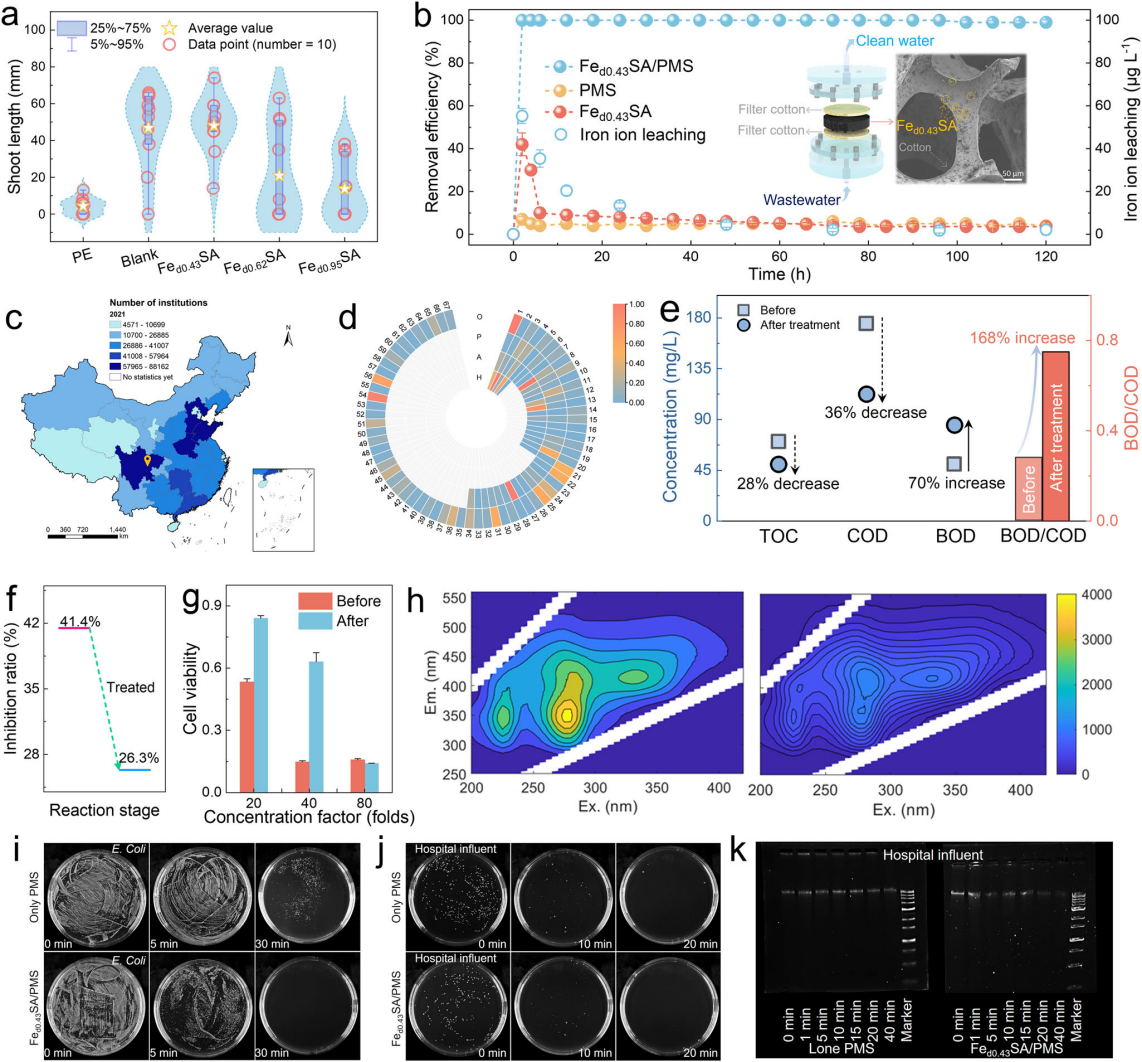
Water purification device integration and continuous operation. (a) Violin plot of wheat shoot length distribution of wheat seed growth in various systems. (b) Performance of Fe_d0.43_SA/PMS in the continuous‐flow mode for PE removal and the concentration of iron ion leaching (inserted plot: schematic illustration of the continuous‐flow mode and SEM image of cotton fibers loaded with Fe_d0.43_SA). Experimental conditions: [catalyst]_column_ = 100 mg, [PMS]_0_ = 0.15 mM, [PE]_0_ = 40 µM, flow rate = 80 mL h^−1^. (c) The distribution of medical and health institutions in China by region, 2021 (the data on the number of medical and health institutions for different provinces were obtained from the National Health Commission of the People's Republic of China, http://www.nhc.gov.cn/). (d) The classification and relative concentrations of pharmaceutical compounds in real hospital wastewater (a hospital in Sichuan province; data were given normalized treatment; H: hormones, A: antibiotics, P: psychotropics, O: other pharmaceuticals; more details in Supporting Information). (e) Water quality parameters (TOC, COD, BOD, and BOD/COD) of hospital wastewater before and after reaction. (f) The evaluation of acute toxicity in hospital wastewater before and after treatment. (g) Concentration effect from the cytotoxicity assays of hospital wastewater. (h) The excitation–emission matrix (EEM) of hospital wastewater before and after treatment. Experimental conditions: [catalyst]_0_ = 100 mg L^−1^, [PMS]_0_ = 0.5 mM, stirring for 1 h. Disinfection of (i) *E. coli* and (j) actual hospital influent. (k) Plasmid DNA agarose gel electrophoresis for the treated actual hospital influent. Error bars are the standard error values of three tests (*n* = 3).

We further assessed the application potential of the ^1^O_2_‐dominated Fe_d0.43_SA/PMS system. The Fe_d0.43_SA/PMS system showed exceptional performances with the presence of different inorganic ions (i.e., Cl^‒^, NO_3_^‒^, H_2_PO_4_^‒^, and HCO_3_^‒^) (Figure S55). Besides, the Fe_d0.43_SA/PMS system achieved 100% PE removal in the different actual water matrices, including lake, river, and tap water (Figure S56a). Furthermore, the acute toxicity of the PE solution was markedly reduced after treatment, as evidenced by a reduced growth inhibition rate in *Photobacterium phosphoreum* T3 from 26.8% to 15.6% (Figure S56b). The wheat growth experiment further validated the detoxification effect, as both germination rate and shoot elongation were markedly improved in Fe_d0.43_SA/PMS‐treated effluent compared to the untreated control. These results underscore the biosafety of the treated wastewater (Figures 6a and S56c). In addition, a continuous‐flow model was established to test the stability of the Fe_d0.43_SA/PMS system, in which cotton fibers were employed as supports to anchor Fe_d0.43_SA and maintain uniform catalyst dispersion, enabling ∼100% PE removal and ultralow iron ion leaching for 120 h (Figures 6b and S57,S58). The cost of treating 1 ton of phenol wastewater is estimated as US$ 0.38 (Figure S59 and Table S8).

As shown in Figure 6c, statistical data indicate that in 2021 alone, China had over 1.03 million medical institutions. Consequently, the treatment of medical wastewater has become an urgent challenge due to the presence of diverse pharmaceutical contaminants that are typically found at low concentrations, exhibit high biological toxicity, and are resistant to conventional biochemical degradation [63]. We analyzed pharmaceutical compositions in hospital wastewater from a hospital in Sichuan province (Figure 6d) and detected 126 compounds, which are divided into hormones, antibiotics, psychotropics, and other pharmaceuticals (Supporting Information). The Fe_d0.43_SA/PMS system was applied to treat real‐world hospital wastewater to assess its application potential. As shown in Figure 6e, despite the moderate removal efficiencies of total organic carbon (TOC, ∼28%) and chemical oxygen demand (COD, ∼36%), the Fe_d0.43_SA/PMS system substantially improved the biodegradability of hospital wastewater. The biochemical oxygen demand (BOD) increased by ∼70% and the BOD/COD ratio rose from 0.28 to 0.75 (∼168% increase), while wastewater samples became noticeably more transparent (Figure S60). The improvement in biodegradability was achieved at a low PMS dosage, underscoring the high oxidant utilization efficiency and selective oxidation characteristics of the ^1^O_2_ system toward refractory, low‐concentration organics typical of hospital wastewater.

Acute and cell toxicity were used to evaluate changes in the toxicity of hospital wastewater after treatment with Fe_d0.43_SA/PMS. Figure 6f shows that the growth inhibition rate substantially declined from 41.4% to 26.3%. Moreover, as shown in Figure 6g, at a low concentration factor, the treated sample promoted cell growth compared with the original sample. Furthermore, the evolution of organic substances in hospital wastewater was evaluated by three‐dimensional fluorescence spectra. As shown in Figure 6h, highly illuminated fluorescent areas were divided into three components (component 1: Ex/Em = 220–250/330–380 nm, component 2: Ex/Em = 250–360/280–380 nm, component 3: Ex/Em = 250–420/380–520 nm), which represents the protein‐tryptophan‐like component, the phenyl ring‐containing proteins and soluble microbial metabolites, and the humic acid‐like substance, respectively [64]. The fluorescence intensity of components 1, 2, and 3 was decreased after treatment, suggesting that abundant ^1^O_2_ effectively oxidized the fluorescent groups in wastewater. Moreover, Figure S61 demonstrates long‐lasting catalytic performance in real hospital wastewater treatment, demonstrating greater efficiency and effectiveness than PMS alone as the disinfectant.

Furthermore, the disinfection of hospital wastewater poses a critical challenge due to the risk of pathogen contamination. To systematically evaluate the bactericidal performance of the Fe_d0.43_SA/PMS system, *Escherichia coli* (*E. coli*, a model Gram‐negative indicator bacterium) was employed in inactivation assays. As shown in Figure 6i, the Fe_d0.43_SA/PMS system achieved 100% bacterial inactivation within 30 min, outperforming PMS alone. To further assess its applicability, the disinfection efficiency of Fe_d0.43_SA/PMS was tested in real hospital wastewater (Figure 6j,k). Although PMS alone exhibited a comparable bactericidal efficiency of 95% within 20 min, DNA gel electrophoresis analysis indicated that intracellular DNA remained intact, highlighting the risk of horizontal gene transfer despite bacterial inactivation. In contrast, the Fe_d0.43_SA/PMS system induced substantial degradation of both intracellular and extracellular DNA. This not only led to irreversible microbial inactivation but also mitigated the potential risk of antibiotic resistance gene transmission, highlighting its promise for advanced disinfection in medical and hospital wastewater treatment.

## 3 Conclusion

In summary, synergistic Fe‒N_4_ twins provide a unique microenvironment for the oriented generation of ^1^O_2_ due to the interplay between d_Fe‒Fe_, spin crossover, and nonradical PMS‐coupling pathways. The optimal d_Fe‒Fe_ (∼0.43 nm) facilitates proximity‐induced electronic interactions between adjacent Fe‒N_4_ sites, forming optimal Fe‒N_4_ twins with IS (t_2g_4e_g_1) state and half‐filled dz^2^ orbitals that overlap effectively with 𝜋* orbital of PMS. Such dz^2^‐𝜋* orbital coupling lowers the activation barrier for PMS coupling and promotes selective ^1^O_2_ generation. The longer lifetime, higher selectivity, and resistance to scavenging of ^1^O_2_ ensure high effective utilization of the oxidant. Moreover, the Fe_d0.43_SA/PMS system demonstrates superior performance in treating real hospital wastewater, efficiently degrading pharmaceutical pollutants and inactivating pathogenic microbes. The treated effluent exhibits greatly improved biocompatibility and a lower risk of transferring antibiotic resistance genes, underscoring its potential for mitigating environmental risks associated with pharmaceutical residues and microbial contamination in pharmaceutical and hospital wastewater treatment.

## Conflicts of Interest

The authors declare no conflicts of interest.

## Supporting information




**Supporting File 1**: anie71430‐sup‐0001‐SuppMat.docx.

## Data Availability

The data that support the findings of this study are available from the corresponding author upon reasonable request.
