# Supplementary material for: Proximity Engineering of Fe‒N4 Twins for Oriented Generation of Singlet Oxygen for Hospital Wastewater Treatment
Source: Angew Chem Int Ed Engl. 2026 Feb 6;65(12):e6249880. doi: 10.1002/anie.6249880 (PMC12990965; doi:10.1002/anie.6249880)
Supplement: Supplementary file 1 — Supporting File 1: anie71430‐sup‐0001‐SuppMat.docx. [file ANIE-65-e6249880-s001.docx]

Supporting Information

**Proximity Engineering of Fe****‒N_4_ Twins for Oriented Generation of Singlet Oxygen for Hospital Wastewater Treatment**

Xinhao Wang^[a], [b], [c]^, Zhaokun Xiong*^[a], [b]^, Shuai Yang^[a], [b]^, Hongyu Zhou^[c]^, Yanbiao Shi^[a], [b]^, Zelin Wu^[a], [b]^, Bingkun Huang^[a], [b]^, Lei Yang^[a], [b]^, Chuan-Shu He^[a], [b]^, Xiaoguang Duan*^[c]^, Bo Lai*^[a], [b]^

[a] Dr. X. Wang, Z. Xiong, S. Yang, Y. Shi, Z. Wu, B. Huang, L. Yang, Prof. C. He, Prof. B. Lai
State Key Laboratory of Hydraulics and Mountain River Engineering, College of Architecture and Environment
Sichuan University
Chengdu 610065, China
E-mail: scuxzk@scu.edu.cn, laibo@scu.edu.cn

[b] Dr. X. Wang, Z. Xiong, S. Yang, Y. Shi, Z. Wu, B. Huang, L. Yang, Prof. C. He, Prof. B. Lai
Sino-German Centre for Water and Health Research
Sichuan University
Chengdu 610065, China
E-mail: scuxzk@scu.edu.cn, laibo@scu.edu.cn

[c] Dr. X. Wang, H. Zhou, Prof. X. Duan
School of Chemical Engineering
The University of Adelaide
Adelaide, SA, 5005 Australia
E-mail: xiaoguang.duan@adelaide.edu.au

**Experimental section:**

**Chemical agents and experimental procedure.** Pyrrole monomers, sodium dodecyl sulfate (SDS, C_12_H_25_OSO_3_Na), ammonium peroxydisulfate (APS), iron(III) 2,4-pentanedionate (Fe(acca)_3_), phenol (PE, 99%), bisphenol A (BPA), sulfisoxazole (SIZ, 99%), sulfamethoxazole (SMX), acyclovir (ACV), carbamazepine (CBZ), atrazine (ATZ), benzoic acid (BA), peroxymonosulfate (PMS, 0.5K_2_SO_4_·0.5KHSO_4_·KHSO_5_), potassium thiocyanate (KSCN), furfuryl alcohol (FFA), 5,5-dimethyl-1-pyrroline N-oxide (DMPO), 2,2,6,6-tetramethyl-4-piperidinyloxyl (TEMP), 9,10-diphenylanthraquinone dyes (DPA), H_2_^18^O, D_2_O, β-carotene, methyl phenyl sulfoxide (PMSO), methyl phenyl sulfone (PMSO_2_), *p*-benzoquinone (*p*-BQ), superoxide dismutase (SOD), 2,2'-azinobis(3-ethylbenzothiazoline-6-sulfonic acid ammonium salt) (ABTS), and CoSO_4_·7H_2_O were purchased from Shanghai Aladdin reagent Inc. Singlet Oxygen Sensor Green (SOSG) was purchased from Meilunbio. Methanol (MeOH), tert-butyl alcohol (TBA), Na_2_S_2_O_3_, H_2_SO_4_, and NaOH were all purchased from the Chengdu Kelong Chemical Reagent Factory. Deionized water was used throughout the experiment. All of the chemicals were of analytical grade or higher and used directly without further purification.

**Synthesis of Fe_dx_SA.** Firstly, the polypyrrole (PPy) hydrogels were prepared. Pyrrole monomers and sodium dodecyl sulfate (SDS) were dissolved in 10 mL of deionized water (solution A). Solution B was a mixed solution of ammonium peroxydisulfate (APS) and deionized water. Solutions A and B were mixed and rested for about 1 hour for polymerization. Then, the PPy hydrogels were washed with deionized water to remove residual reagents. Afterward, iron(III) 2,4-pentanedionate (Fe(acac)_3_) was dissolved in 40 mL of EtOH to serve as the metal source (solution C). PPy hydrogels were dipped in solution C and stirred for 10 h (Fe-PPy hydrogels). To control the distribution of Fe sites, several concentrations of Fe(acac)_3_ were used: 0.01 M, 0.05 M, and 0.10 M. Subsequently, Fe-PPy hydrogels undergo the process of EtOH washing, pyrolysis, acid washing, and then pyrolysis to synthesize Fe_dx_SA. To clarify the catalysts, Fe_dx_SA were labeled with the measured d_Fe‒Fe_ values, which were named Fe_d0.43_SA, Fe_d0.62_SA, and Fe_d0.95_SA corresponding to samples prepared using 0.1 M, 0.05 M, and 0.01 M Fe(acac)_3_, respectively. Additionally, CN and FeSA (wt% = 4.19%) were synthesized using the same method as FedxSA, except for the concentration of Fe(acac)_3_ (CN: 0 M, FeSA (wt% = 4.19%): 0.075 M).

**Fenton-like reaction procedure.** All experiments were carried out in a 250 mL glass beaker containing 100 mL of PE (40 μM) with a constant stirring rate of 300 rpm at 30 ± 1 °C. Each reaction was started with the required dosages of catalysts and PMS. At each time interval, 0.22 μm polytetrafluoroethylene syringe filter discs were used to filter the samples, which were then quenched by Na_2_S_2_O_3_ solution (50 μL) before analysis. The initial pH was adjusted by H_2_SO_4_ or NaOH after the addition of PMS. All experiments were carried out in duplicate or triplicate, and the data obtained were averaged.

**Detection of the pollutants.** PE concentration was analyzed by reversed-phase high-performance liquid chromatography (Agilent, USA) using an Eclipse XDB C18 column (5 μm, 4.6 × 250 mm). The mobile phase consisted of formic acid (0.1%) and acetonitrile with a ratio of 50:50 (v/v) at a flow rate of 1.0 mL min^‑1^, and the column temperature was set at 30 °C (**Table S6**).

**Chemical detection of singlet oxygen.** SOSG was applied to the detection of ^1^O_2_ generation. The 500 μM SOSG stock solution was prepared by dissolving 100 μg of SOSG in 300 μL MeOH. Fe_dx_SA (1 mg) and SOSG (30 μL) were added to 2950 μL DI water under stirring, and PMS (20 μL, the concentration of the store solution: 10 mM) was added to the breaker after 5 seconds. 200 μL of the sample was withdrawn and diluted to 2 mL at 1, 2, 3, 4, and 5 min. The sample was measured by a fluorescence spectrophotometer (F-7100 FL 220-240 V, Hitachi) immediately. The excitation wavelength and emission wavelength were 480 nm and 500 nm (start) ‒ 650 nm (end).

Additionally, the 9,10-diphenylanthraquinone dyes (DPA) were used to further confirm the generation of ^1^O_2_ and semi-quantitatively assess production. The stock solution of DPA (1 mM) was prepared in a mixture of acetonitrile and chloroform (4:1, v/v). The DPA solution (50 μM, diluted from stock) was treated with PMS in the presence of different Fe_dx_SA. The treated solution was collected using a poly-sery HLB (Anpel, Shanghai) solid-phase extraction (SPE) cylinder. The production of DPAO_2_ was analyzed by UPLC-Q-TOF-MS/MS (Agilent 1290 Infinity II UPLC with Agilent G6545 Q-TOF). The formic acid (0.1%) and methanol were used as mobile phases A and B, respectively. The flow was 0.2 mL min^-1^. Furthermore, the scan range for the intermediate mass was 50-800 m/z, with positive-mode electrospray ionization (ESI+). An Agilent Infinity Lab Poroshell 120 EC-C18 column (2.1 × 50 mm, 1.9 μm) was used to separate the samples, with the column temperature set at 40 °C.

**The** **recovery method of catalysts in the cycling experiments.** Separate the catalysts (after 20 min reaction) from the reaction solution by suction filtration. Then, the catalysts were washed with DI water several times, and the catalysts were separated from the filter paper into the new solution by transient ultrasound (for about 2-3 seconds).

**The extended X-ray absorption fine structure (EXAFS) measurements.** EXAFS measurements were carried out on the sample at 21 Al X-ray nanodiffraction beamline of Taiwan Photon Source (TPS), National Synchrotron Radiation Research Center (NSRRC). This beamline adopted a 4-bounce channel-cut Si (111) monochromator for mono-beam X-ray nanodiffraction and X-ray absorption spectroscopy. The end-station is equipped with three ionization chambers and a Lytle/SDD detector after the focusing position of KB mirror for transmission and fluorescence mode X-ray absorption spectroscopy. The photon flux on the sample ranges from 1 × 10^11^ ~ 3 × 10^9^ photon/sec for X-ray energy from 6 - 27 k eV.

The data for the L-edge XANES measurement were collected on the soft X-ray spectroscopy (SXRS) beamline at the Australian Synchrotron (Melbourne) in a transition mode at room temperature.

**Temperature-dependent magnetization (M) measurements.** ZFC/FC susceptibility curves were conducted under H = 500 Oe and variable-temperature (0-400 K) for temperature-dependent magnetization measurement by PPMS-SQUID system. The *μ*_eff_ is obtained by *μ*_eff_ = $\sqrt{\text{8}\text{C}}$μ_B_ according to the Langevin theory. μ_B_ means the Bohr magneton, *C* is Curie constant and obtained from the fittings on the susceptibility (χ = *M*/*H*) obeyed by Curie–Wiess law: χ = *C*/(*T*–Θ), where Θ is Curie–Weiss temperature. Besides, the number of unpaired electron (n) is calculated via equation 1.

$\mu_{eff}=\sqrt{n\left(n+2\right)}$ (equation 1)

**Mössbauer spectra measurements:** Mössbauer spectroscopy was measured using Iron Analytics with a transmission detector. Each spectrum was collected under room temperature condition for more than 48 h.

**In-situ Raman analysis.** Raman spectroscopy (Renishaw InVia Raman microscope) was obtained with a He-Ne laser (λ = 532 nm) as the excitation source. First, Catalysts were placed on a glassy carbon sheet and pressed lightly. PMS solution (1 M) was dropped onto the catalysts. Then, the glassy carbon sheet was scanned from 700 to 1200 cm^−1^. The irradiation did not induce PMS decomposition for PMS alone in the experiment.

**Activity evaluation.**

The kinetic rate constants (*k*_obs_) of PE degradation were further analyzed, and the kinetic equation is as follows:

$\text{ln}\frac{\text{C}_{\text{t}}}{\text{C}_{0}}=\text{-}k_{\text{obs}}\times\text{t}$ (equation 2)

Where C_t_ is the concentration of contaminants at t min, C_0_ is the initial concentration of contaminants, and t is the reaction time point.

The SSA normalized *k*_obs_ (*k*_obs_/SSA) was analyzed to evaluate the contribution of SSA of catalysts, and the equation is as follows:

$\text{SSA normalized }k_{\text{obs}}\text{=}\frac{k_{\text{obs}}}{\text{SSA}}$ (equation 3)

Where *k*_obs_ is calculated via equation S1 and *SSA* is the specific surface area of catalysts.

The Fe loading normalized *k*_obs_ (*k*_obs_/Fe-loading) was analyzed to evaluate the contribution of Fe loading of catalysts, and the equation is as follows:

$\text{Fe loading normalized }k_{\text{obs}}\text{=}\frac{k_{\text{obs}}}{\text{Fe-loading}}$ (equation 4)

Where *k_obs_* is calculated via equation S1 and Fe-loading was measured via ICP-OES.

Turnover frequency (TOF) was analyzed to evaluate the intrinsic activity of catalysts, and the equation is as follows:

$\text{TOF}=\frac{\text{C}_{0}\times\text{(contaminants removal/\%)}}{\text{t}\times\text{C}_{\text{Fe}}}$ (equation 5)

$\text{C}_{\text{Fe}}=\frac{\text{C}_{\text{cata.}}\times\text{(Fe content/\%)}}{56}$ (equation 6)

Where C_0_ is the initial concentration of contaminants (mM), *t* is the reaction time, C_Fe_ is the Fe element concentration (mM), and C_cata._ is the concentration of the catalyst (mg L^-1^).

**Quantification of singlet oxygen.** FFA was used as a probe to determine the steady-state concentration of ^1^O_2_ ([^1^O_2_]_ss_), employing a second-order reaction rate constant of 1.2×10^8^ M^-1^ s^-1^ for the interaction between ^1^O_2_ and FFA. It was assumed that the physical quenching of ^1^O_2_ by FFA could be disregarded, allowing for the calculation of the steady-state concentration of ^1^O_2_ using the equations provided as follows:

$\frac{\text{d}\left[\text{FFA}\right]}{\text{dt}}=-k_{{}^{1}\text{O}_{2}\text{,FFA}}\left[{}^{1}\text{O}_{2}\right]_{\text{ss}}\left[\text{FFA}\right]$ (equation 7)

$\left[{}^{1}\text{O}_{2}\right]_{\text{ss}}=\frac{\text{ln}\left(\left[\text{FFA}\right]_{0}\text{/}\left[\text{FFA}\right]_{\text{t}}\right)}{\text{t}\times k_{{}^{1}\text{O}_{2}\text{,FFA}}}$ (equation 8)

**Electrochemical measurements.** Electrochemical measurements were carried out on a CHI 660E electrochemical workstation using a conventional three-electrode system: platinum as the counter electrode, a saturated calomel electrode as the reference electrode, and a glassy carbon electrode as the working electrode. Place the three electrodes in a 50 mL beaker containing 0.5 M Na_2_SO_4_ solution of 25 mL. Among them, the glassy carbon electrode needs to be polished on the polishing cloth. The sample (4 mg), ethanol (960 μL), and Nafion reagent (40 μL) are mixed and dispersed ultrasonically for 30 minutes, then uniformly spread onto the conductive surface of the glassy carbon electrode. When the working electrode is air-dried, install the electrodes and start testing.

**DFT calculation method.** We have employed the first-principles calculations^[1, 2]^ to perform density functional theory (DFT) calculations within the generalized gradient approximation (GGA) using the Perdew-Burke-Ernzerhof (PBE)^[3]^ formulation. We have chosen the projected augmented wave (PAW) potentials^[4]^ to describe the ionic cores, and we have included valence electrons using a plane-wave basis set with a kinetic-energy cutoff of 400 eV. First principles calculations employed the Vienna Ab-initio Simulation Package (VASP).^[5, 6]^ The exchange-correlation effects were described by the Perdew-Burke-Ernzerhof (PBE) functional within the generalized gradient approximation (GGA) method.^[3, 7]^ The core-valence interactions were accounted by the projected augmented wave (PAW) method.^[4]^ The energy cutoff for plane wave expansions was set to 520 eV. The structural optimization was completed for energy and force convergence set at 1.0×10^-5^ eV and 0.02 eV Å^-1^, respectively. The Brillouin zone was sampled with a 2×2×1 grid centered at the gamma (Γ) point. Grimme’s DFT-D3 methodology^[6]^ was used to describe the dispersion interactions. Finally, the adsorption energies (E_ads_) were calculated as E_ads_ = E_ad/sub_ - E_ad_ - E_sub_, where E_ad/sub_, E_ad_, and E_sub_ are the total energies of the optimized adsorbate/substrate system, the adsorbate in the structure, and the clean substrate, respectively. Due to the magnetism of transition metal elements, spin polarization was considered in all calculations. We established initial magnetic moments for Fe atom based on the possible valence states and conducted single point energy calculations.

**Analysis of the oxidation products**

For analysis of the dissolved products transformation, the reaction solution was filtered by 0.22 µm PTFE membranes. Solution was quenched by MeOH and subjected to UPLC-Q-TOF-MS/MS (Agilent 1290 Infinity II UPLC with Agilent G6545 Q-TOF) with an electron spray ionization (ESI) source. The formic acid (0.1%) and methanol were used as mobile phases A and B, respectively. The flow rate was 0.2 mL/min, and the gradient elution conditions are shown in **Table S7**. The ion-transfer capillary temperature was 500 ◦C during the ESI-MS analysis. Furthermore, the scan range for intermediate mass was from 50 to 800 m/z in positive mode ESI+ for SIZ and ACV, and in negative mode ESI- for PE and BPA. An Agilent InfinityLab Poroshell 120 EC-C18 column (2.1 × 50 mm, 1.9 µm) was used to separate samples with the column temperature at 40 ◦C.

**Disinfection experiments.** The counts of viable fecal bacteria in the water sample before and after the disinfection process were determined using the plate colony-counting method. The beef extract solid medium, the liquid medium, and the 0.9% sodium chloride solution were sterilized using a high-pressure steam sterilizer.

Genomic DNA was extracted using a bacterial DNA extraction kit, confirmed by DNA agarose gel electrophoresis (at 120 V for 40 min with 1% agarose gel in 1 × TAE buffer), and imaged using a gel imaging system (Tanon 3500B, Tanon B, China).

**The acute toxicity experiments.** The acute toxicity of samples was evaluated based on the ISO 11348-3 test protocol. The bioluminescence emitted by the photobacterium *Vibrio fischeri* can change with toxicity. The luminescence intensity was measured by Microplate Reader (Biotek Synergy H1), and the inhibition was calculated. The solution pH of every sample was adjusted to 6.8 (NaOH and H_2_SO_4_).

The inhibition ratio (IR) of different samples was calculated as equation 9.

IR = 1 − (Abs_s_-Abs_b_)/(Abs_n_-Abs_b_) (equation 9)

where Abs_s_, Abs_b_, and Abs_n_ represent the absorbance at 450 nm for each sample, blank control (only added 2% NaCl solution without Vibrio Fischer), and negative control (added 2% NaCl solution with Vibrio Fischer), respectively.

**The cytotoxicity experiments.** The solid phase extraction (SPE) was first performed to concentrate the solution. The SPE cartridges (Poly-Sery HLB Pro 500 mg, 6 mL, Anpel, China) were activated with 10 mL of methanol and 10 mL of ultrapure water. Then, the pH of the sample was adjusted to 2.00 with H_2_SO_4_ and passed through the extraction cartridges at a flow rate of 5 mL min^-1^. After the extraction, the SPE cartridges were eluted with 10 mL of methanol and further dried under a gentle stream of nitrogen. The obtained organic extracts were then dissolved in 800 μL of 2.5% DMSO and 2% NaCl solution for the cytotoxicity assay.

The cytotoxicity assay was then developed by the Cell Counting Kit-8 (CCK-8, Dojindo Laboratories, Japan) containing the effective component tetrazolium salt 2-(2-methoxy-4-nitrophenyl)-3-(4-nitrophenyl)-5-(2,4-disulfophenyl)-2H-tetrazolium monosodium salt (WST-8). Briefly, organic extracts from SPE were diluted to different concentrations corresponding to different concentration factors (0.5% DMSO dissolved in culture medium), then added to cells in 96-well plates and incubated in an incubator for 48 h. The 96-well plates were then washed with phosphate buffer solution (PBS). CCK-8 dilution (dissolved in DMEM/F12 medium) was added to the 96-well plates. After incubation at 37°C for 1-2 h, the number of viable cells was quantified by reading the absorbance at 450 nm with a microplate reader (BioTek, China). The cell viability (CV) of different samples was calculated as equation 10.

CV = (A_s_-A_b_)/(A_n_-A_b_) (equation 10)

where A_s_, A_b_ and A_n_ represent the absorbance for samples, blank control (CCK-8 reagent dissolved in DMEM/F12 only), and negative control (0.5% DMSO dissolved in culture medium only), respectively.**Supplementary Text**

**H (Hormones):** 1. Cortisone, 2. Norethynodrel, 3. Progesterone, 4. Scopolamine, 5. Triamcinolone acetonide;

**A (Antibiotics):** 1. Acyclovir, 2. Azithromycin, 3. Buspirone, 4. Carbamazepine, 5. Cefazolin, 6. Cefradine, 7. Chloramphenicol, 8. Dexamethasone, 9. Fluconazole, 10. Nifedipine, 11. Norfloxacin, 12 Rifampicin, 13. Tramadol, 14. Clarithromycin, 15. Erythromycin ethyl succinate, 16. Roxithromycin, 17. Sulfisoxazole, 18. Sulfacetamide, 19. Sulfaguanidine, 20. Sulfamerazine, 21. Sulfisoxazole;

**P (Psychotropics):** 1. Amitriptyline, 2. Brucine, 3. Citalopram, 4. Clozapine, 5. Diazepam, 6. Doxepin, 7. Felodipine, 8. Flunarizine, 9. Fluoxetine, 10. Fluphenazine, 11. Fluvoxamine, 12. Isoprenaline, 13. Lorazepam, 14. Meprobamate, 15. Methorphan, 16. Midazolam, 17. Nikethamide, 18. Paroxetine, 19. Phenytoin, 20. Quetiapine, 21. Quinidine, 22. Reserpine, 23. Risperidone, 24. Sertraline, 25. Tetracaine, 26. Tiapride, 27. Triazolam, 28. Venlafaxine, 29. Ziprasidone, 30. Chlordiazepoxide, 31. Chlorpromazine, 31. Clomipramine, 32. Estazolam, 33. Fenfluramine;

**O (Other pharmaceuticals):** 1. Atropine, 2. Benazepril, 3. Bisoprolol, 4. BroMbuterol, 5. Butyl 4-aminobenzoate, 6. Caffeine, 7. Carvedilol, 8. Cetirizine, 9. Chenodeoxycholic acid, 10. Cimbuterol, 11 Cisapride, 12. Cyproheptadine, 13. Demoxepam, 14. Difenidol, 15. Diphenoxylate, 16. Fenbufen, 17. Futalin, 18. Gemfibrozil, 19. Glipizide, 20. Ibuprofen, 21. Labetalol, 22. Lamotrigine, 23. Levonorgestrel, 24. Mabuterol, 25. Metoprolol, 26. Mizolastine, 27. Naltrexone, 28. Naproxen, 29. Phenacetin, 30. Pidotimod, 31. Propranolol, 32. Pseudoephedrine, 33. Sildenafil, 34. Salbutamol, 35. Sulfaphenazolum, 36. Sulpiride, 37. Telmisartan, 38. Terazosin, 39. Terbutaline, 40. Tolazamide, 41. Trimetazidine, 42. Warfarin, 43. Warfarin sodium, 44. Zopiclone, 45. Chloroquine, 46. Clenbuterol, 47. Domperidone, 48. Fentanyl, 49. Gliclazide, 50. Glimepiride, 51. Hexythiazox, 52. Hydroflumethiazide, 53. Indapamide, 54. Lidocaine, 55. Lofexidine, 56. Isoprenaline, 57. Mebendazole, 58. Mirtazapine, 59. Naltrexone, 60. Pidotimod, 61. Primidone, 62. Procainamide, 63. Propafenone, 64. Ractopamine, 65. Trifluoperazine, 66. Trihexyphenidyl, 67. Tiapride.

**Figure S1.** The relationship between the concentration of Fe(acac)_3_ and the loading amount.

Notes: The concentration of Fe(acac)_3_ is the amount of metal salt introduced; the loading amount is the amount of metal loaded after synthesis.


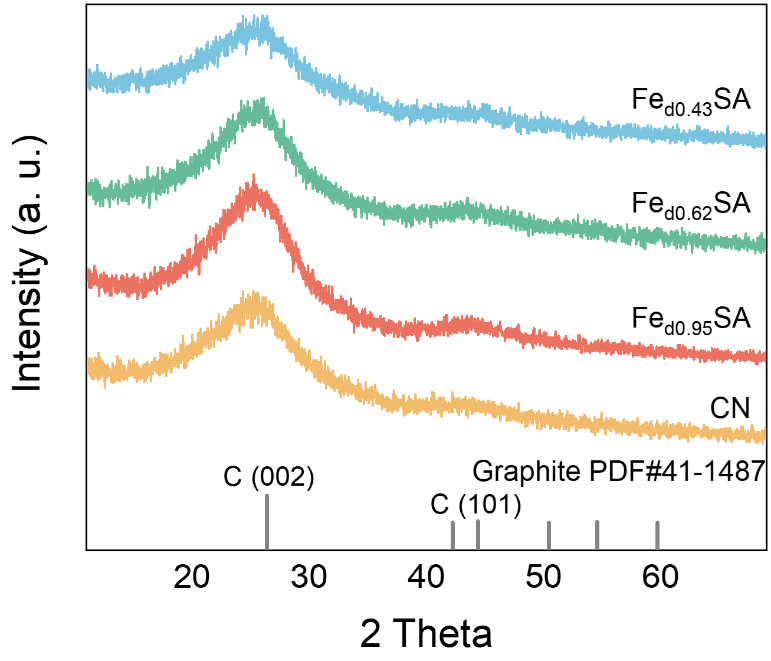


**Figure S2.** XRD patterns of CN, Fe_d0.43_SA, Fe_d0.62_SA, and Fe_d0.95_SA.


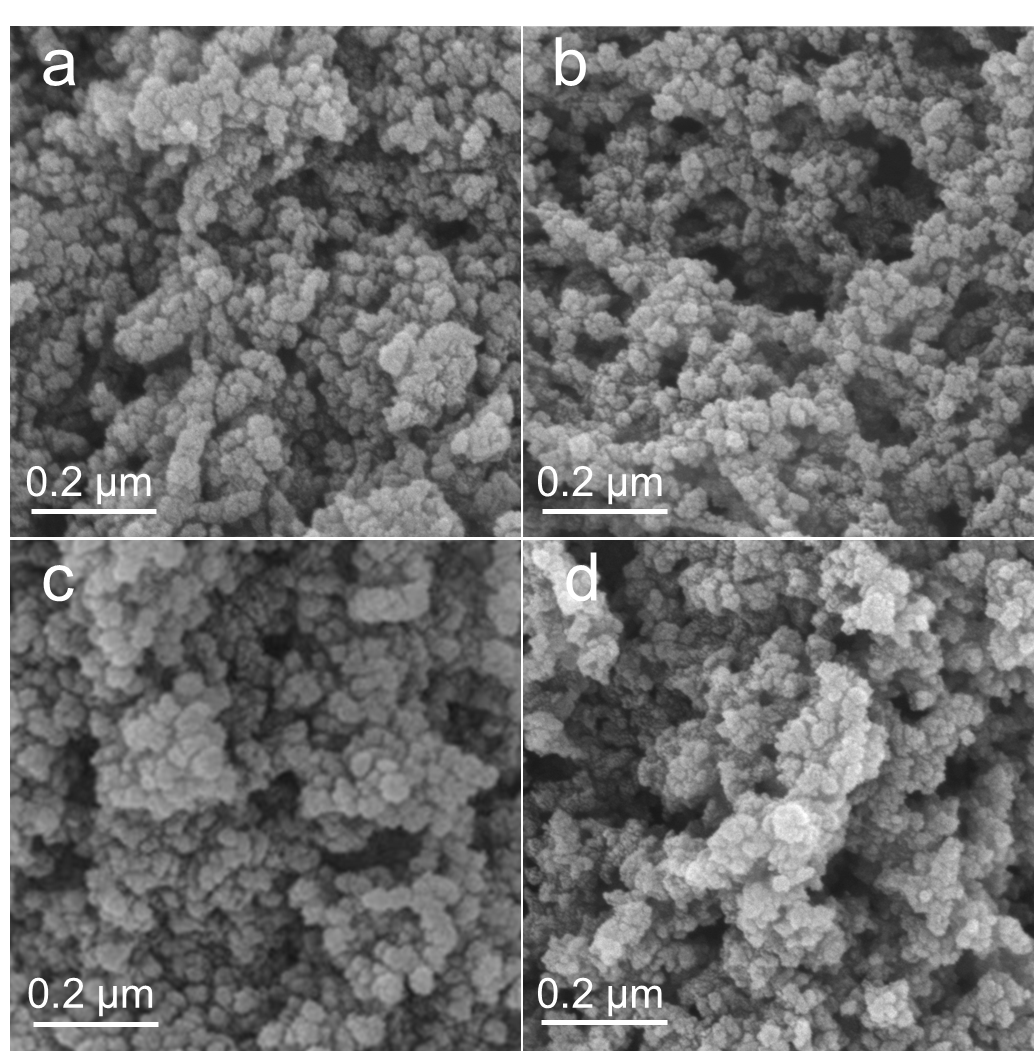


**Figure S3.** SEM images of (a) CN, (b) Fe_d0.43_SA, (c) Fe_d0.62_SA, and (d) Fe_d0.95_SA.


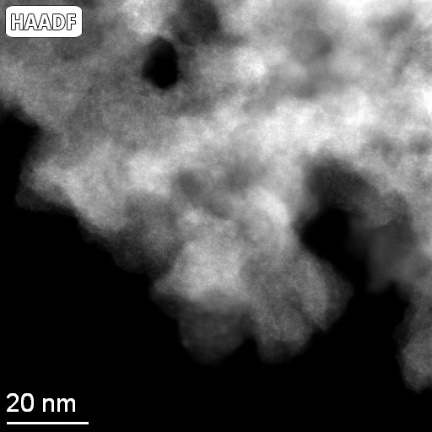

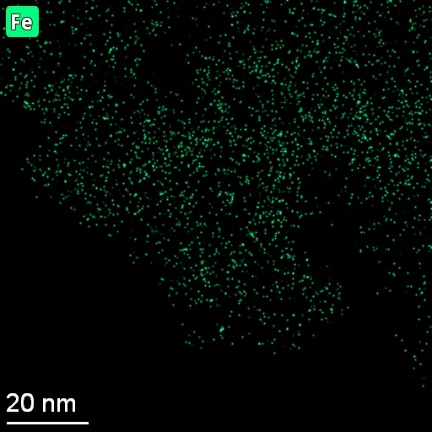

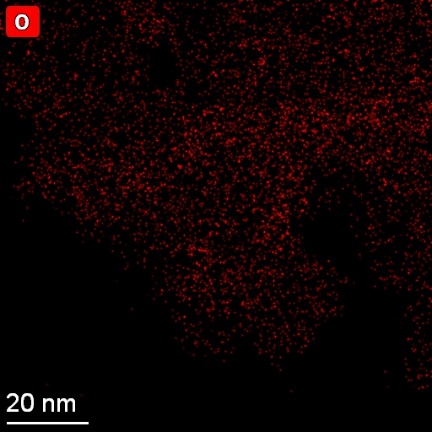

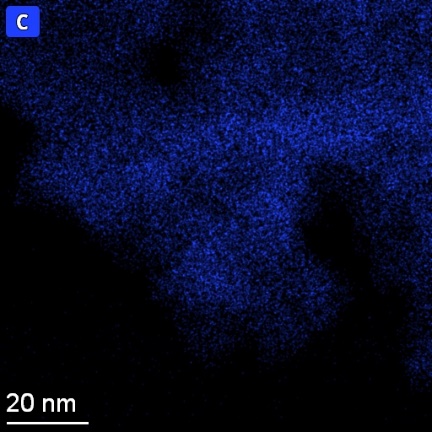


**Figure S4.** HAADF-STEM image of Fe_d0.43_SA corresponding EDS mappings.


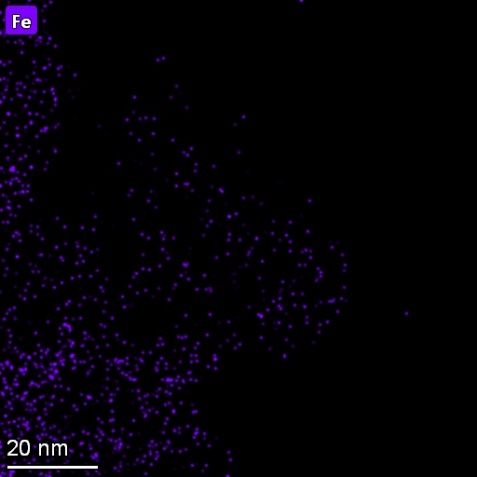

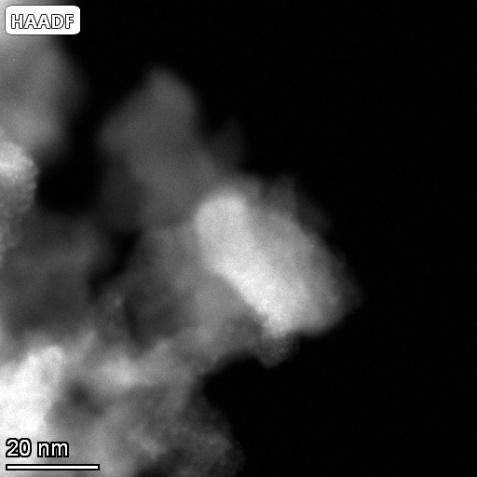

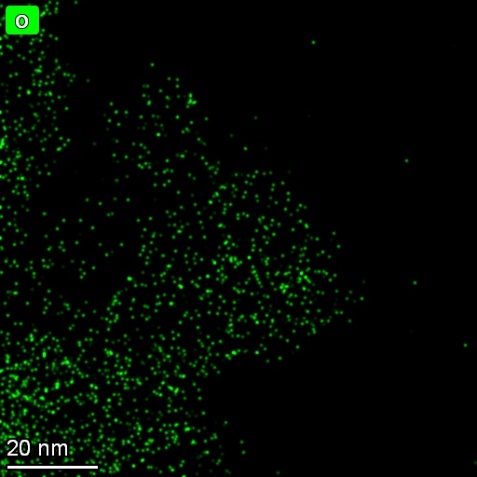

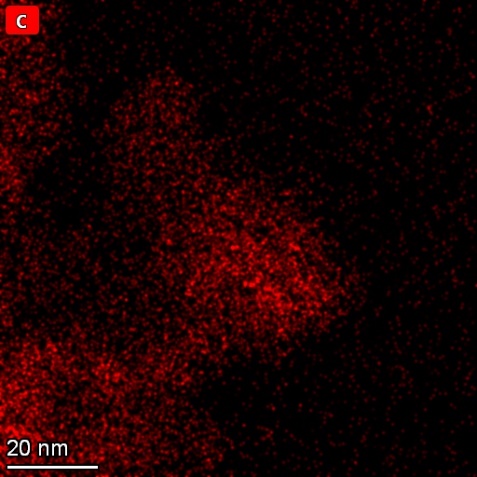


**Figure S5.** HAADF-STEM image of Fe_d0.62_SA corresponding EDS mappings.


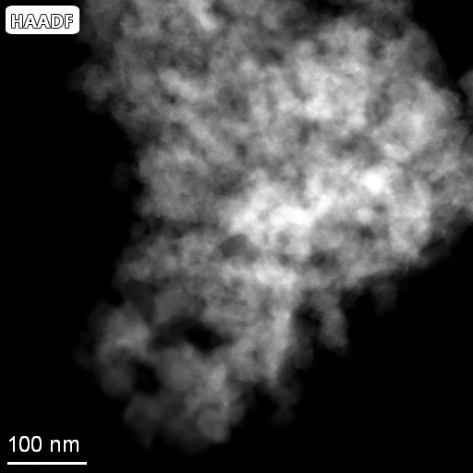

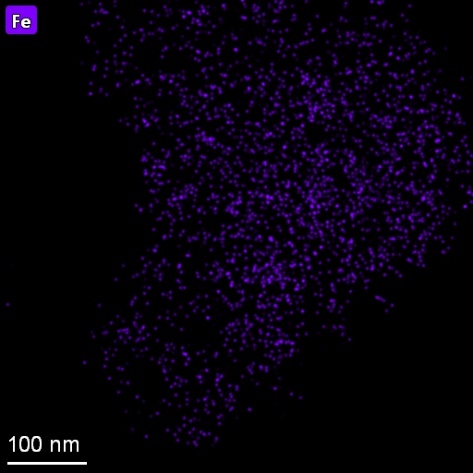

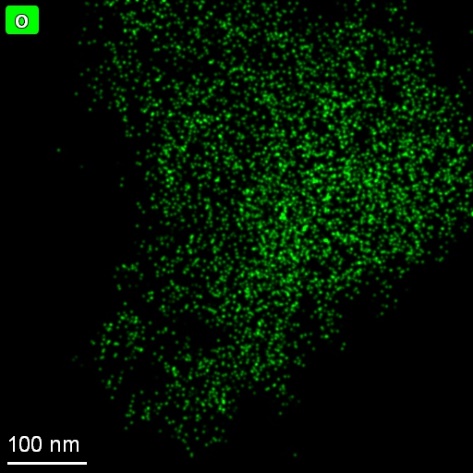

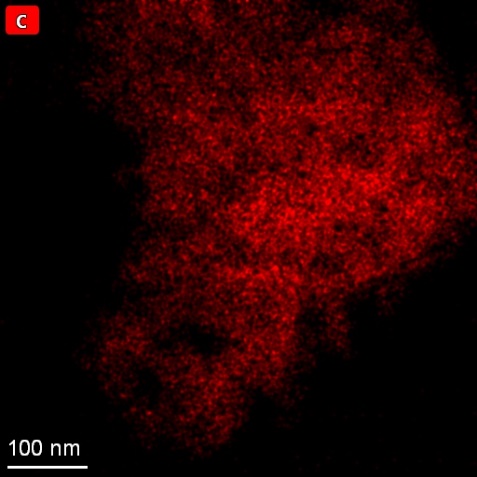


**Figure S6.** HAADF-STEM image of Fe_d0.95_SA corresponding EDS mappings.


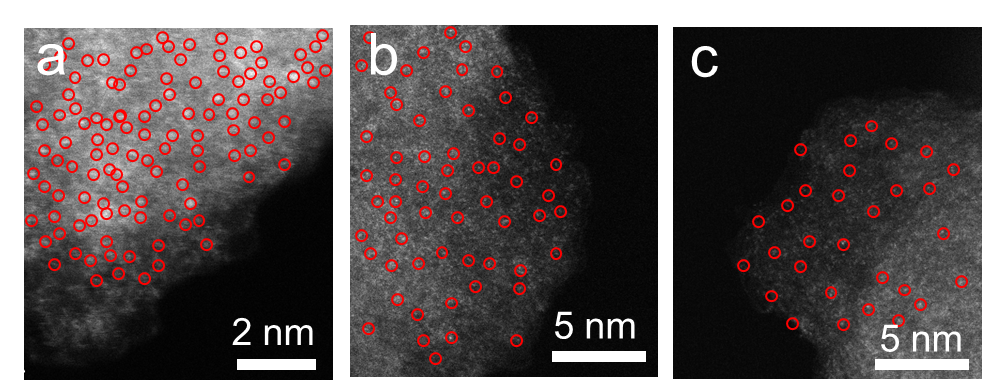


**Figure S7.** AC-HAADF-STEM image of (a) Fe_d0.43_SA, (b) Fe_d0.62_SA, (c) Fe_d0.95_SA.


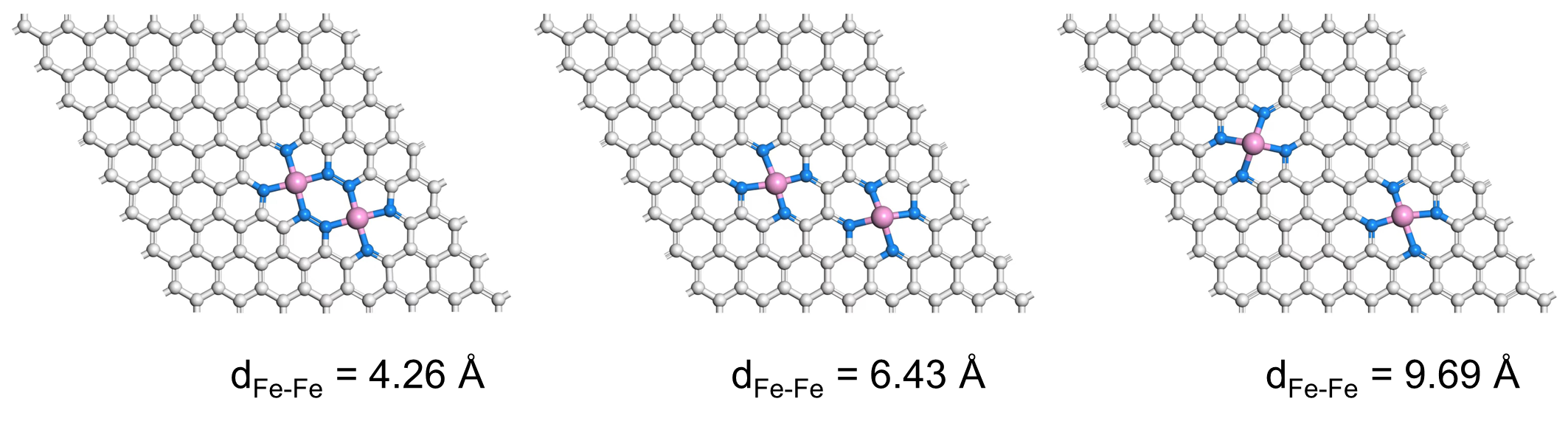


**Figure S8.** Computed structural models of the adjacent Fe atoms. There are 4% errors between the model structures and statistical results about d_Fe-Fe_, which are caused by the Fine-tuning of atoms during structure optimization.

**
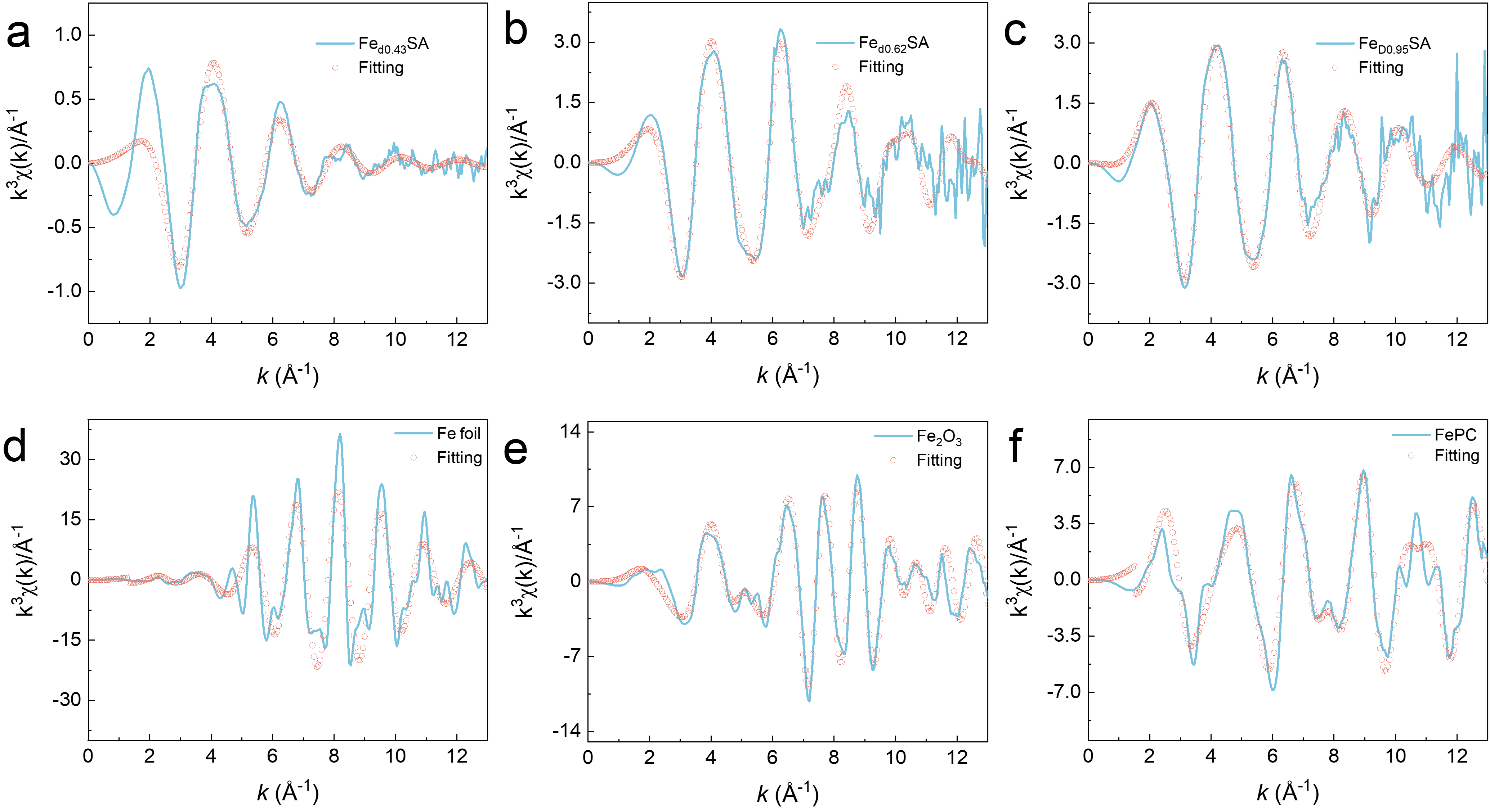
**

**Figure S9.** K-space EXAFS spectra of (a) Fe_d0.43_SA, (b) Fe_d0.62_SA, (c) Fe_d0.95_SA, (d) Fe foil, (e) Fe_2_O_3_, and (f) FePc.

**Figure S10.** Wavelet transform analysis of (a) Fe foil and (b) Fe_2_O_3._

**_
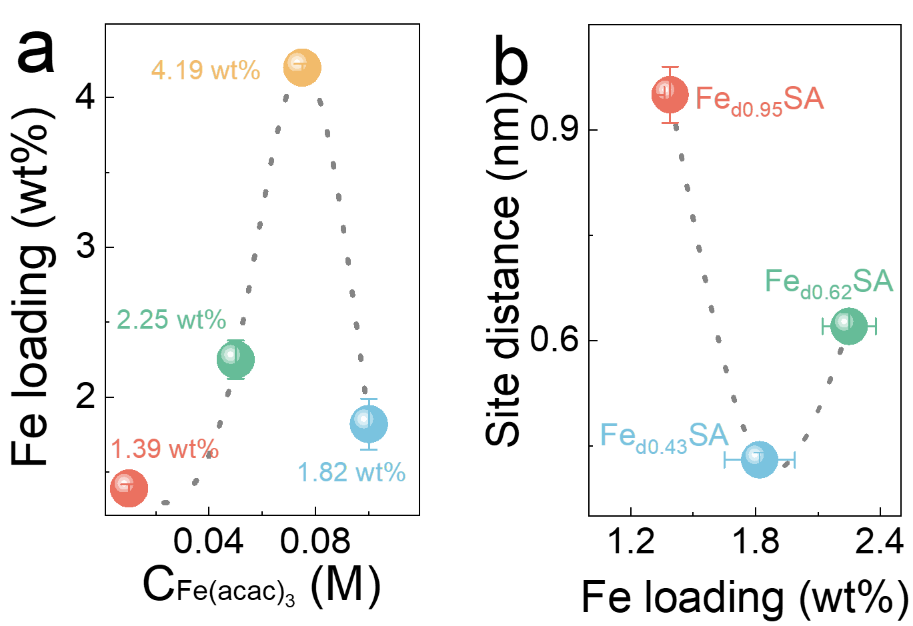
_**

**Figure S11.** Relation between (a) concentration of Fe(acac)_3_ and Fe loading and (b) between Fe loading and site distance.


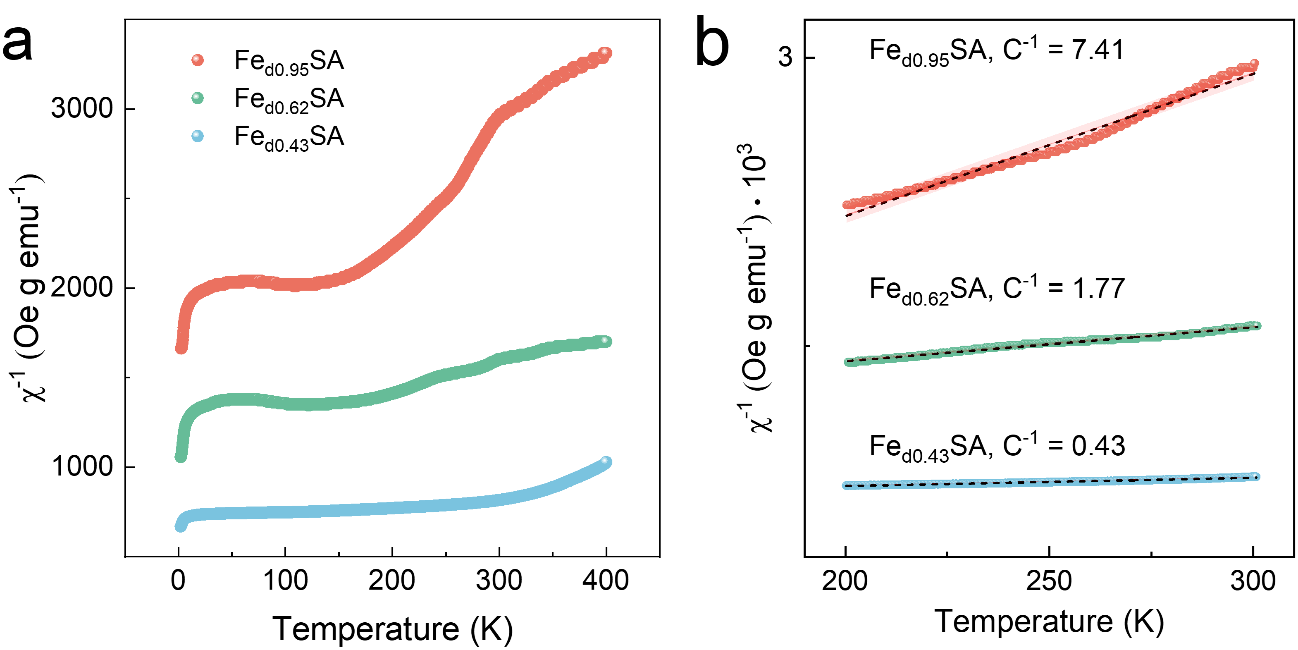


**Figure S12.** Temperature-dependent magnetic susceptibility with the Curie−Weiss fittings for Fe_dx_SA.


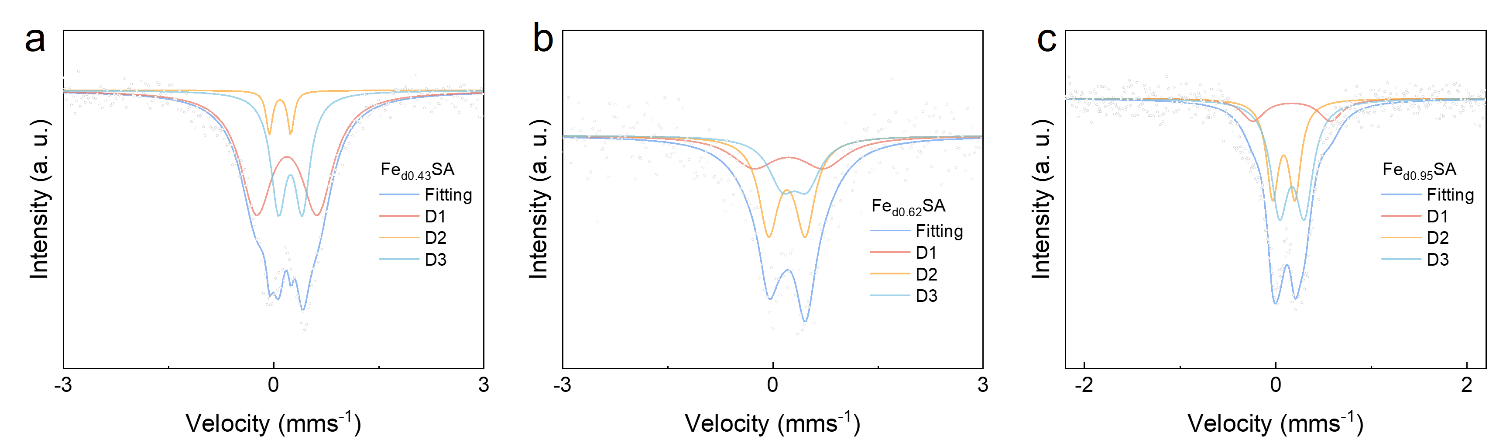


**Figure S13.** Fitted ^57^Fe Mössbauer spectra of as-prepared Fe_d0.43_SA, Fe_d0.62_SA, and Fe_d0.95_SA catalysts.

**Figure S14.** The total DOS of (a) Fe_d0.43_SA, (b) Fe_d0.62_SA, and (c) Fe_d0.95_SA.


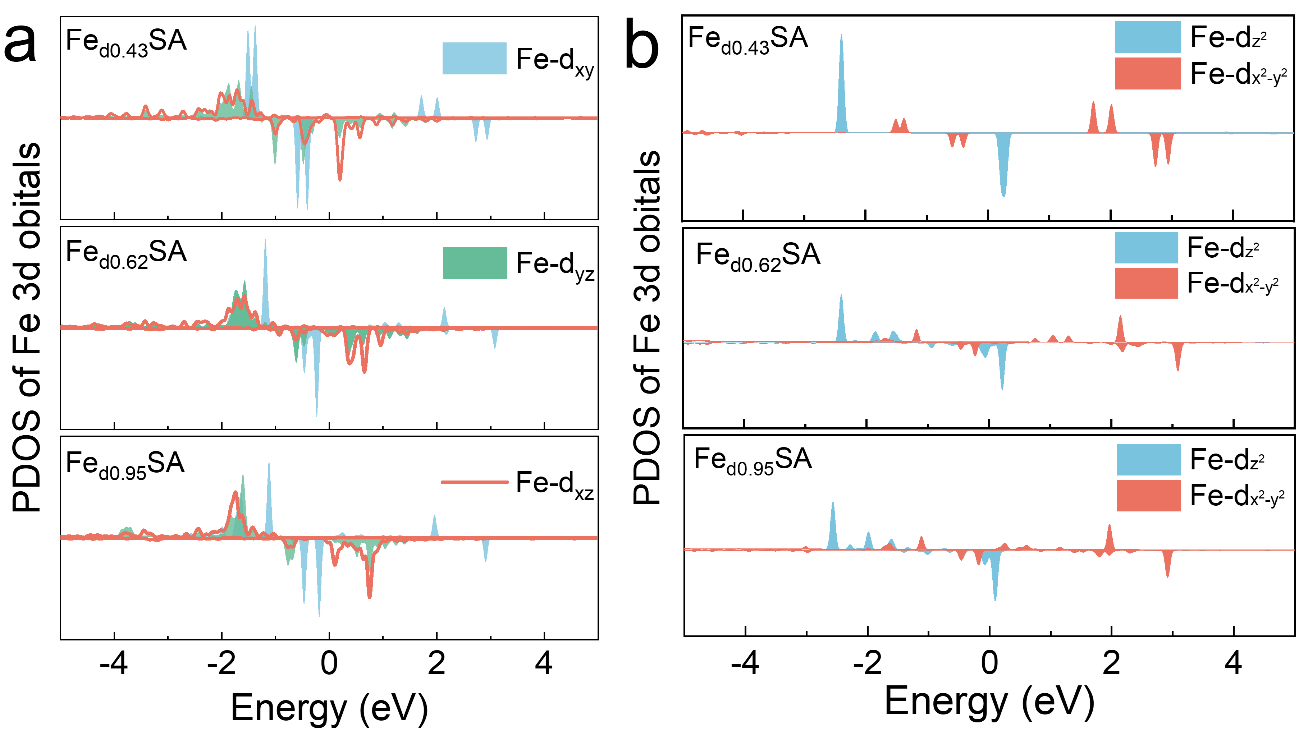


**Figure S15.** PDOS plots of Fe (a) t_2g_ orbitals and (b) e_g_ orbitals in Fe_dx_SA.

**Figure S16.** The influence of (a) Fe_d0.43_SA dosage, (b) PMS dosage for PE removal. (c) The effect of Fe_d0.43_SA dosage for PE adsorption. Experimental conditions (unless otherwise specified): [catalyst]_0_ = 100 mg L^-1^, [PMS]_0_ = 0.3 mM, [PE]_0_ = 40 μM. Error bars are standard error values of three tests (n = 3).

**Figure S17.** The adsorption rates of Fe_dx_SA for PE. Experimental conditions (unless otherwise specified): [catalyst]_0_ = 100 mg L^-1^, [PE]_0_ = 40 μM. Error bars are standard error values of three tests (n = 3).

**Figure S18.** The nitrogen adsorption and desorption curves of (a) CN, (b) Fe_d0.43_SA, (c) Fe_d0.62_SA, and (d) Fe_d0.95_SA.

**Figure S19.** Raman spectra of Fe_dx_SA.

**
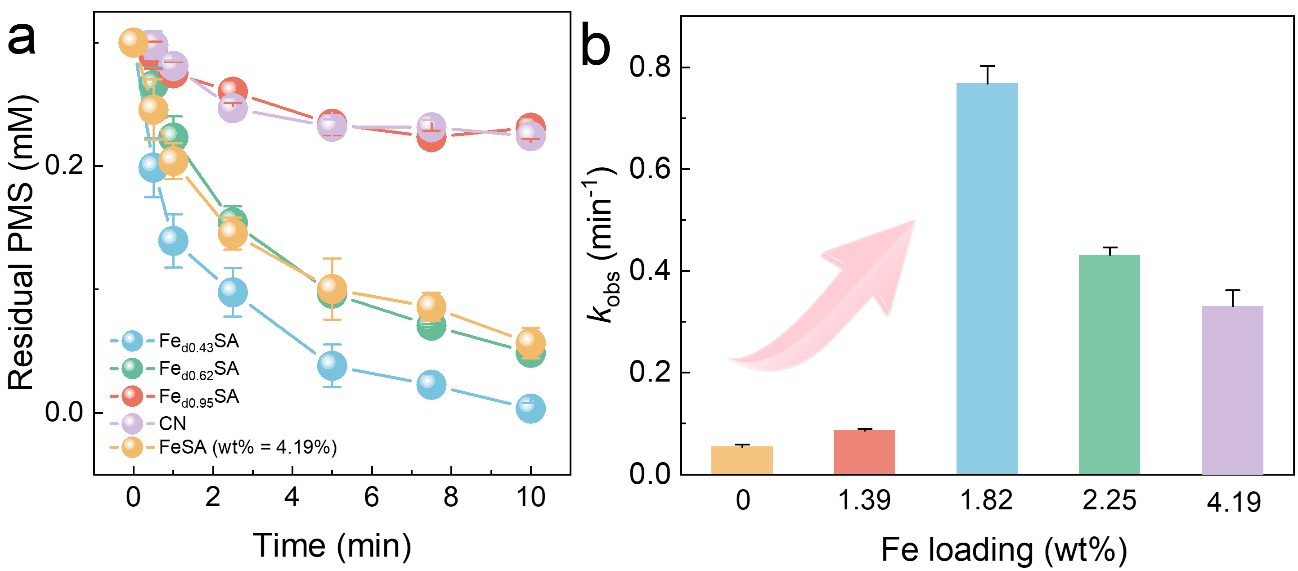
**

**Figure S20.** (a) Residual PMS concentration during the reaction. (b) Relations between *k*_obs_ and Fe loading. Experimental conditions (unless otherwise specified): [catalyst]_0_ = 100 mg L^-1^, [PMS]_0_ = 0.3 mM, [PE]_0_ = 40 μM. Error bars are the standard error values of three tests (n = 3).


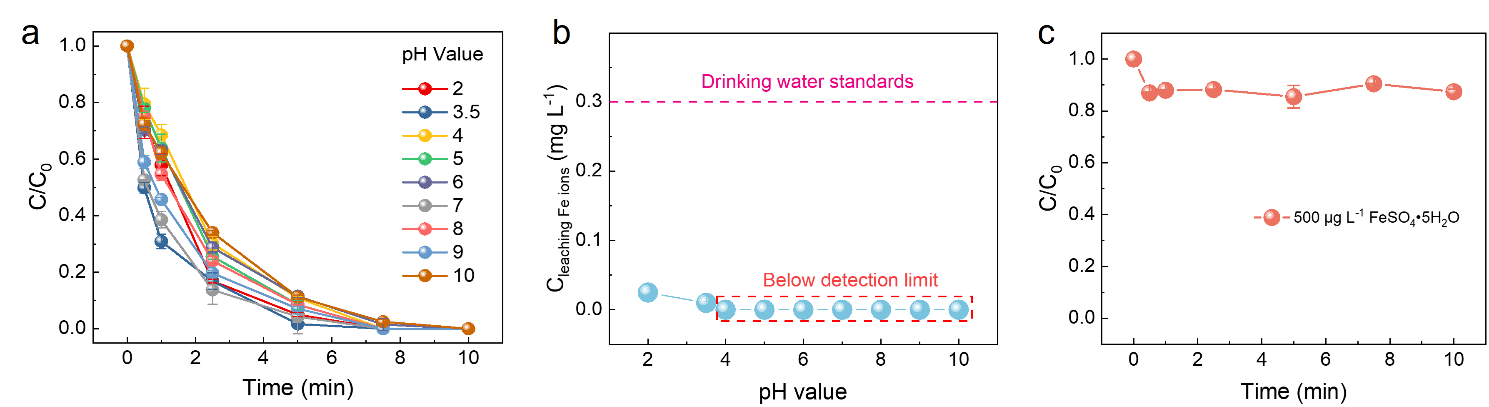


**Figure S21.** The (a) PE removal rate and (b) iron ions leaching in Fe_d0.43_SA/PMS under different initial pH values (2.0-10.0). The pH was adjusted with H_2_SO_4_ and NaOH after PE and PMS mixing, and the unadjusted pH value was 3.5. (c) The degradation of PE in the Fe^2+^/PMS system. Experimental conditions (unless otherwise specified): [catalyst]_0_ = 100 mg L^-1^, [PMS]_0_ = 0.3 mM, [PE]_0_ = 40 μM. Error bars are the standard error values of three tests (n = 3).

Notes: Homogeneous experiments demonstrate that the trace-level leached iron ions are insufficient to effectively activate PMS for rapid PE degradation, confirming the negligible contribution of homogeneous iron species in the Fe_d0.43_SA/PMS system.

**Figure S22.** Effects of scavengers (IPA, MeOH, EtOH, and TBA) on (a) Fe_d0.43_SA/PMS, (b) Fe_d0.62_SA /PMS, (c) Fe_d0.95_SA /PMS, and (d) CN/PMS to remove PE. Experimental conditions: [catalyst]_0_ = 100 mg L^-1^, [PMS]_0_ = 0.3 mM, [PE]_0_ = 40 μM, [IPA]_0_ = [MeOH]_0_ = [EtOH]_0_ = [TBA]_0_ = 300 mM. Error bars are the standard error values of three tests (n = 3).


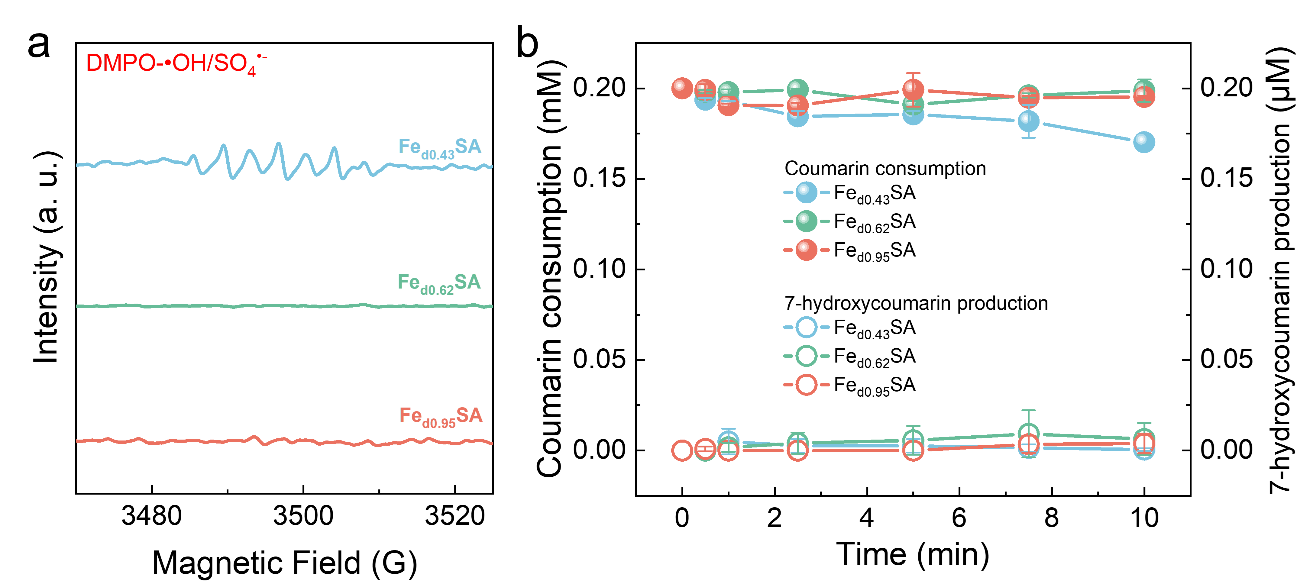


**Figure S23.** (a) EPR spectra in Fe_dx_SA/PMS systems with DMPO as the trapping agent of ^•^OH and SO_4_^•‒^. (b) Coumarin as a probe to quantify ^•^OH in Fe_dx_SA/PMS systems. Experiment condition: [catalyst]_0_ = 100 mg L^-1^, [PMS]_0_ = 0.3 mM, and [Coumarin]_0_ =0.2 mM. Error bars are the standard error values of three tests (n = 3).

Notes: Very low coumarin consumption and extremely low 7-hydroxycoumarin production indicate negligible generation of ^•^OH in all systems.

**Figure S24.** Pre-mixing experiment: Effects of pre-mixing time on (a) Fe_d0.43_SA/PMS, (b) Fe_d0.62_SA/PMS, (c) Fe_d0.95_SA/PMS, and (d) CN/PMS. Experiment condition: [catalyst]_0_ = 100 mg L^-1^, [PMS]_0_ = 0.3 mM, and [PE]_0_ = 40 μM. Error bars are the standard error values of three tests (n = 3).

**Figure S25.** The open-circuit potential.

**Figure S26.** PMSO consumption and PMSO_2_ production in (a) CN/PMS, (b) Fe_d0.43_SA/PMS, (c) Fe_d0.62_SA/PMS, and (d) Fe_d0.95_SA/PMS in the presence of PE. PMSO consumption and PMSO_2_ production in (e) CN/PMS, (f) Fe_d0.43_SA/PMS, (g) Fe_d0.62_SA/PMS, and (h) Fe_d0.95_SA/PMS in the absence of PE. Experimental conditions: [catalyst]_0_ = 100 mg L^-1^, [PMS]_0_ = 0.3 mM, [PE]_0_ = 40 μM, and [PMSO]_0_ = 40 μM. Error bars are the standard error values of three tests (n = 3).

**
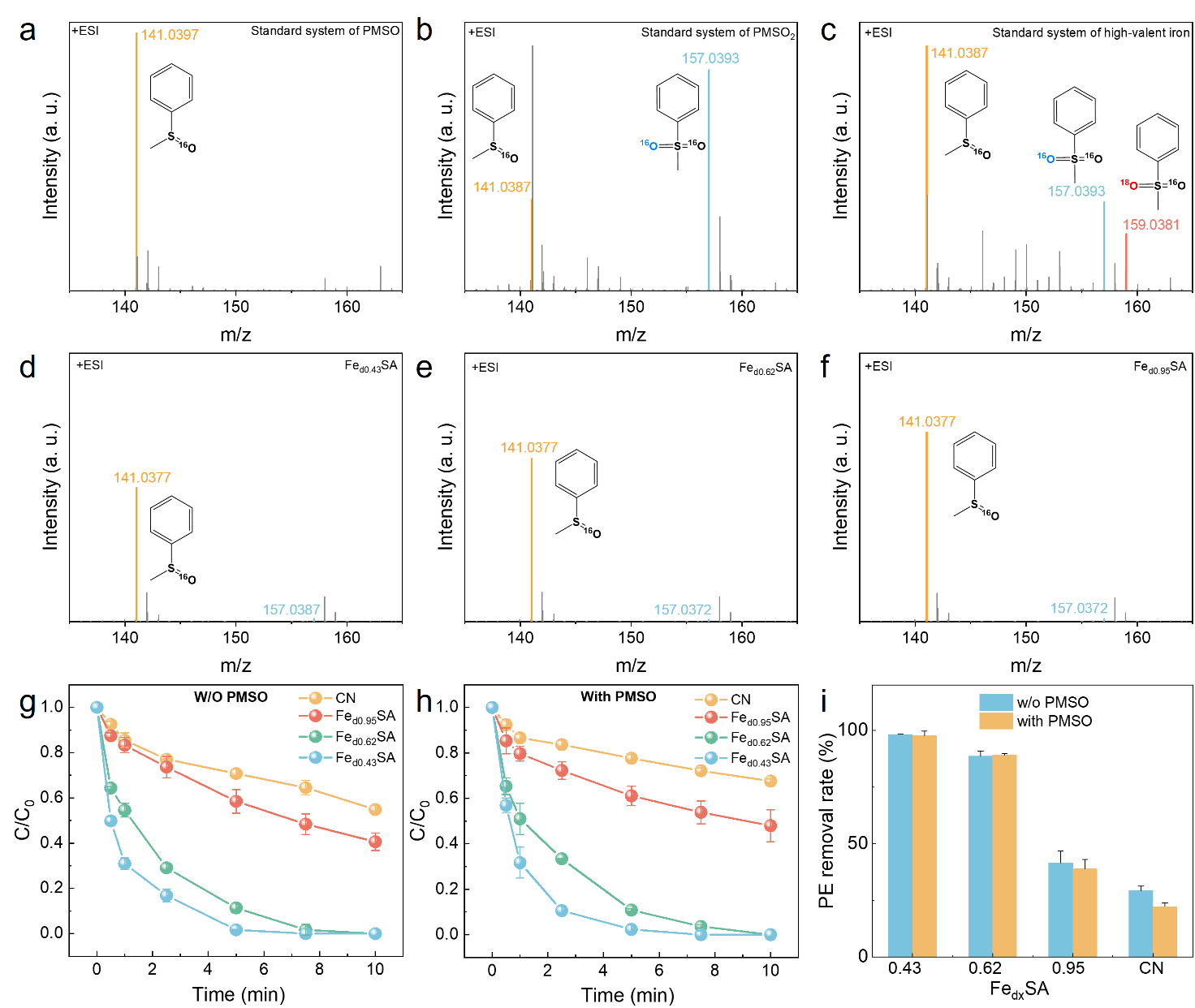
**

**Figure S27.** The UPLC-QTOF-MS/MS spectra of PMSO, PMSO_2_, and PMS^16^O^18^O in (a) PMSO solution, (b) PMSO_2_ solution, (c) Ferrate(VI)/PMSO system with H_2_^18^O isotope-labeling, (d) Fe_d0.43_SA/PMS/PMSO system with H_2_^18^O isotope-labeling, (e) Fe_d0.62_SA/PMS/PMSO system with H_2_^18^O isotope-labeling, (f) Fe_d0.95_SA/PMS/PMSO system with H_2_^18^O isotope-labeling. The degradation of PE at different time intervals in Fe_dx_SA/PMS systems in the (g) absence/(h) presence of PMSO. (i) The comparison of the PE degradation rate in different systems with or without PMSO. Experimental conditions: [catalyst]_0_ = 100 mg L^-1^, [PMS]_0_ = 0.3 mM, [PE]_0_ = 40 μM, and [PMSO]_0_ = 40 μM. Error bars are the standard error values of three tests (n = 3).

Notes: The Fe‒O bond in high valent iron exchanges O atoms with solvent, so ^18^O isotope-labeling PMSO_2_ (PMS^16^O^18^O) was also determined. When the Ferrate(VI)/PMSO system transferred to the H_2_^18^O matrix, two peaks with m/z = 157.0393 and m/z = 159.0381 were detected (**Figure S27**c), ascribed to PMS^16^O^16^O, and PMS^16^O^18^O, suggesting that ^18^O isotope occurred oxygen atom transfer reaction among H_2_^18^O, Ferrate(VI) and PMSO. However, the characteristic peaks of PMS^16^O^16^O and PMS^16^O^18^O were not detected in Fe_dx_SA/PMS/PMSO systems (**Figure S27**d-f), excluding the generation of high-valent iron-oxo.


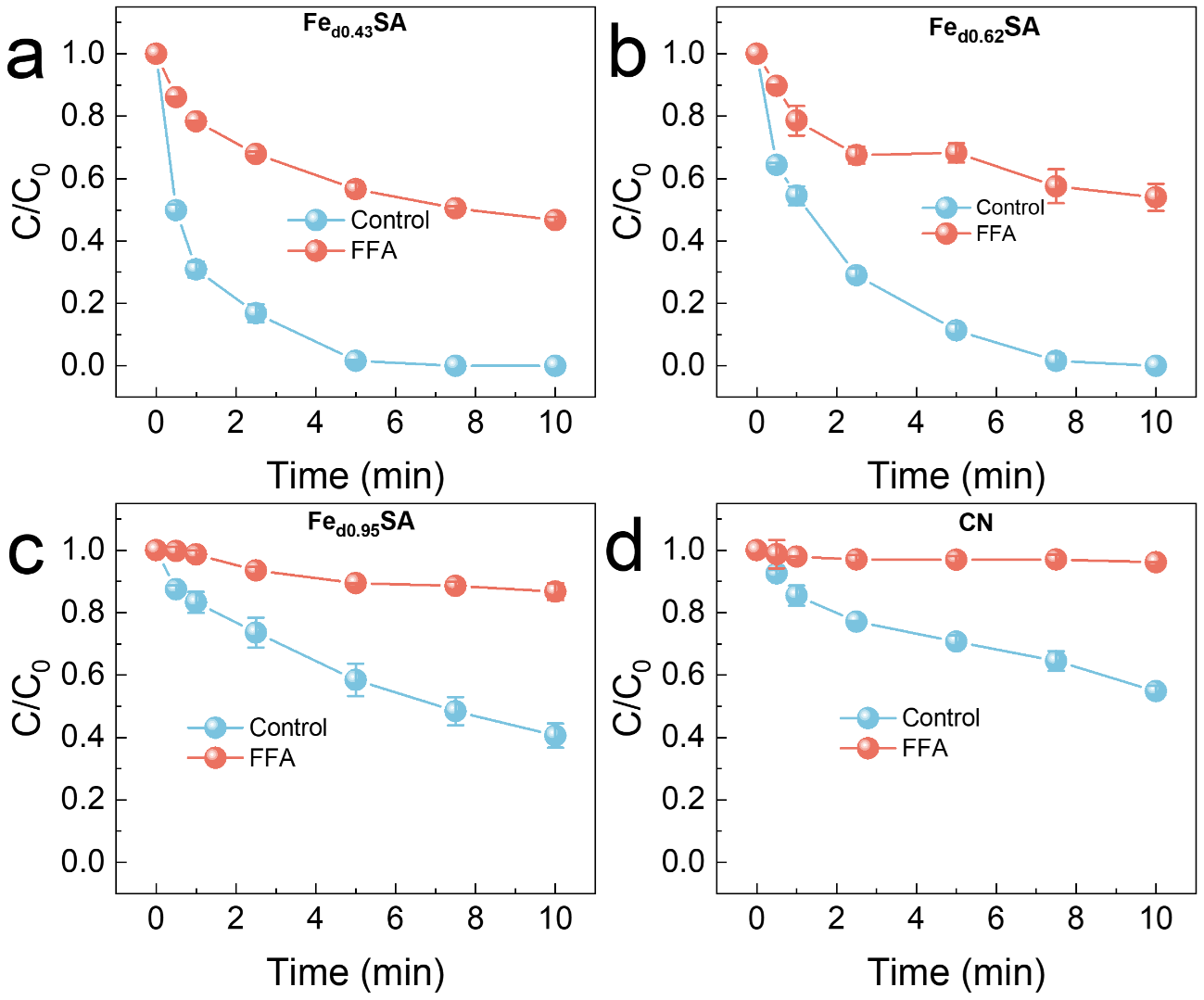


**Figure S28.** Effects of scavengers (FFA, and *p*-BQ) on (a) Fe_d0.43_SA/PMS, (b) Fe_d0.62_SA /PMS, (c) Fe_d0.95_SA /PMS, and (d) CN/PMS to remove PE. Experimental conditions: [catalyst]_0_ = 100 mg L^-1^, [PMS]_0_ = 0.3 mM, [PE]_0_ = 40 μM, [FFA]_0_ = 10 mM, and [*p*-BQ]_0_ = 5 mM. Error bars are standard error values of three tests (n = 3).

**Figure S29.** Effects of *β*-carotene on (a) CN/PMS, (b) Fe_d0.43_SA/PMS, (c) Fe_d0.62_SA/PMS, (d) Fe_d0.95_SA/PMS to PE removal in pure MeOH. Experimental conditions: [catalyst]_0_ = 100 mg L^-1^, [PMS]_0_ = 0.3 mM, and [PE]_0_ = 40 μM. Error bars are the standard error values of three tests (n = 3).

**Figure S30.** Pseudo-first-order rate constants of different systems.

**Figure S31.** The effect of different concentrations SOD addition for PE removal on (a) Fe_d0.43_SA/PMS, (b) Fe_d0.62_SA /PMS, (c) Fe_d0.95_SA /PMS, and (d) CN/PMS. Experimental conditions: [catalyst]_0_ = 100 mg L^-1^, [PMS]_0_ = 0.3 mM, and [PE]_0_ = 40 μM. Error bars are the standard error values of three tests (n = 3).


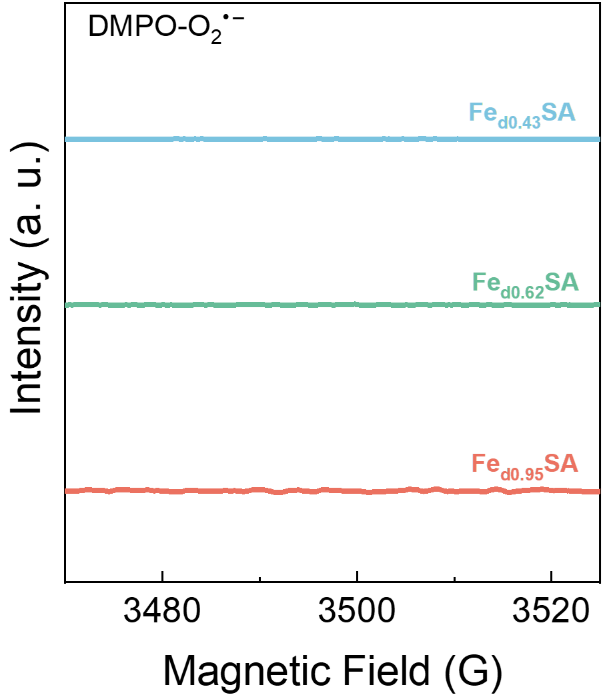


**Figure S32.** EPR spectra in Fe_dx_SA/PMS systems with DMPO as a trapping agent of O_2_^•‒^.

**Figure S33.** Fluorescence spectra of SOSG-EP in (a) PMS only and (b) CN/PMS systems.


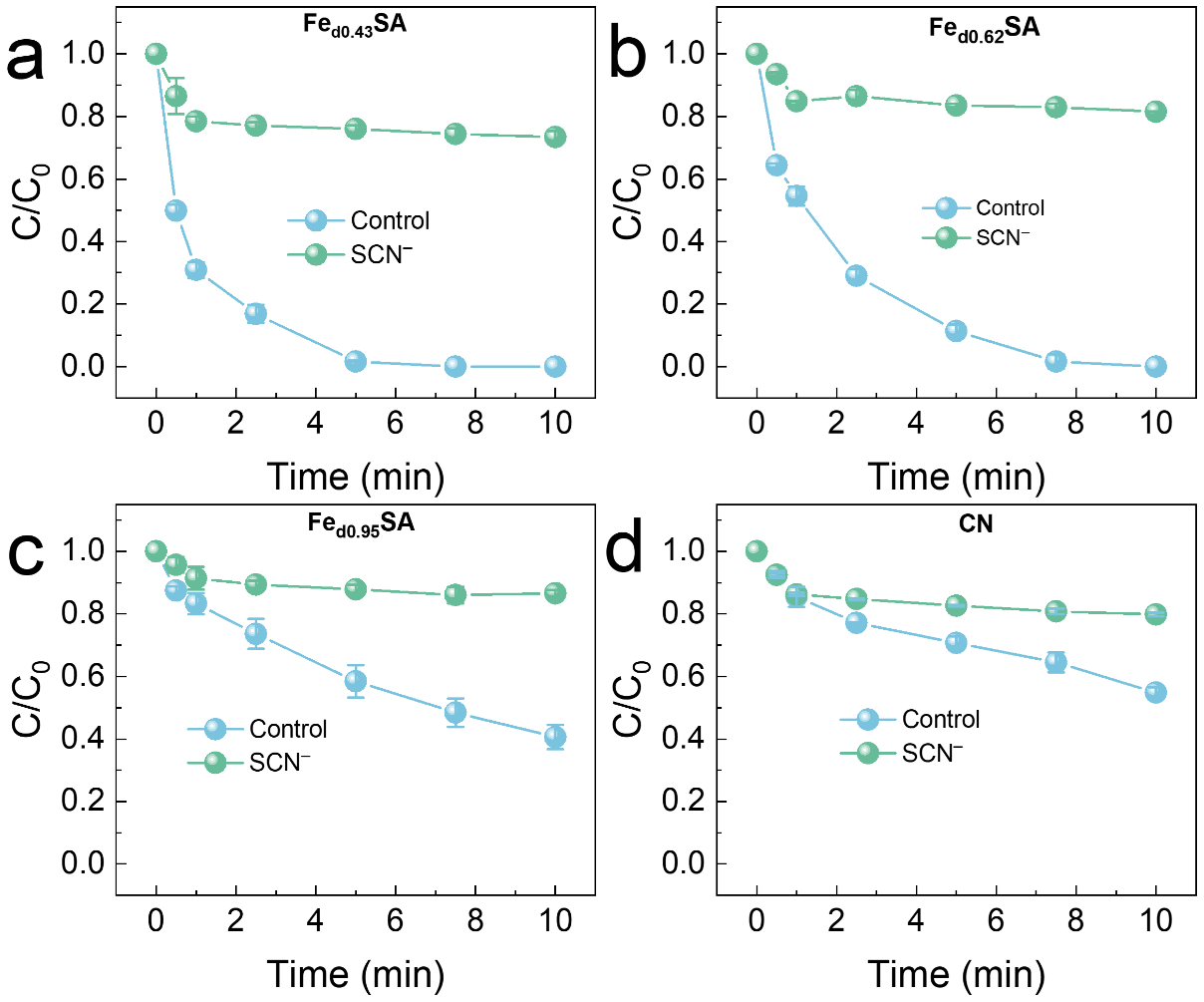


**Figure S34.** Effects of SCN^‒^ on (a) Fe_d0.43_SA/PMS, (b) Fe_d0.62_SA /PMS, (c) Fe_d0.95_SA /PMS, and (d) CN/PMS to remove PE. Experimental conditions: [catalyst]_0_ = 100 mg L^-1^, [PMS]_0_ = 0.3 mM, [PE]_0_ = 40 μM, and [SCN^‒^] = 100 mM. Error bars are the standard error values of three tests (n = 3).


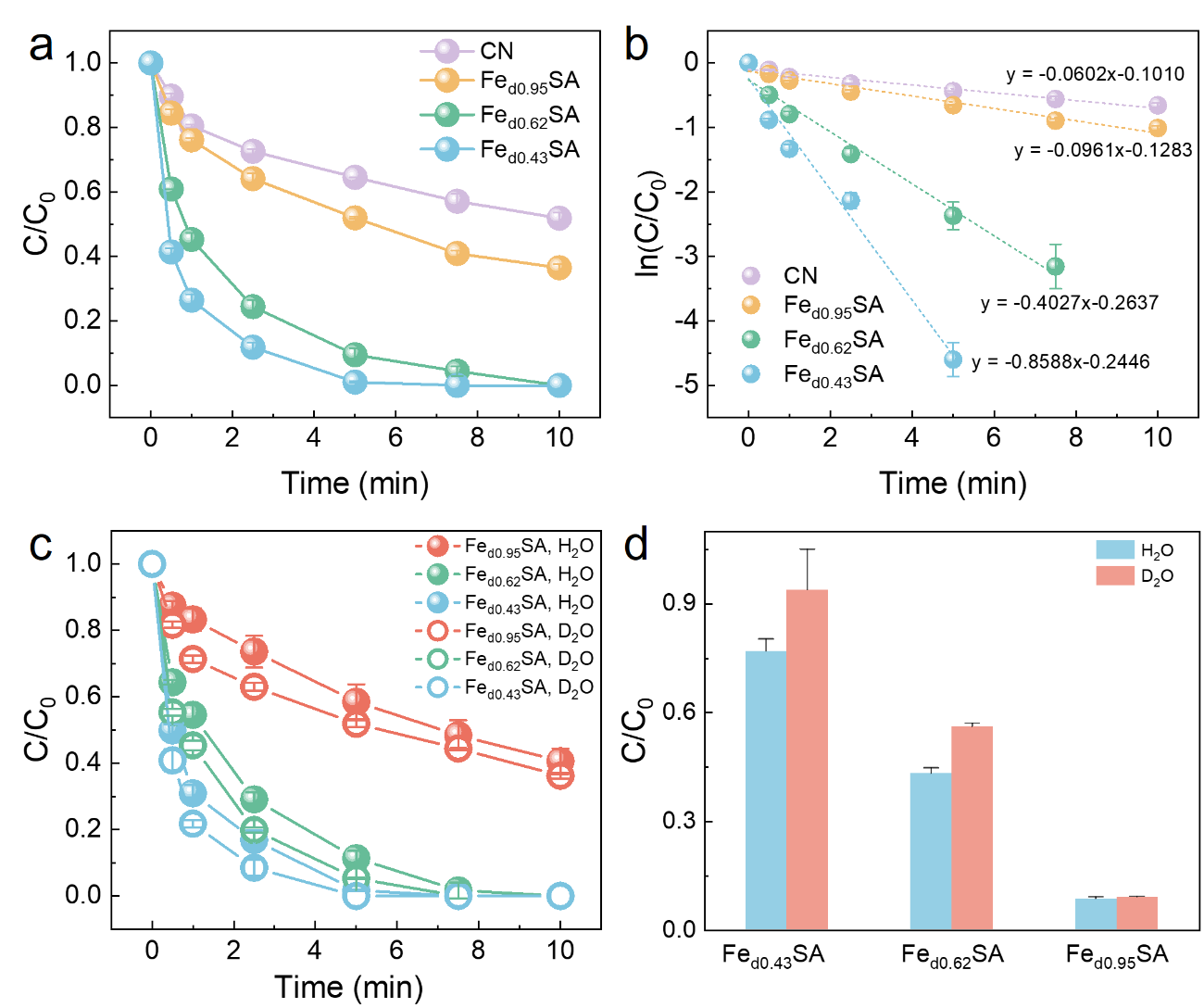


**Figure S35.** (a) The degradation rate of FFA and (b) the corresponding pseudo-first-order rate constants in different Fe_dx_SA systems. (c) Effect of reaction solvents (H_2_O and D_2_O) on PE degradation and (d) the corresponding rate constants in different Fe_dx_SA/PMS systems. Experimental conditions: [catalyst]_0_ = 100 mg L^-1^, [PMS]_0_ = 0.3 mM, [FFA]_0_ = 50 μM, and [PE]_0_ = 40 μM.

Notes: FFA was used as a probe to measure the [^1^O_2_]ss, with the second-order reaction rate constant between ^1^O_2_ and FFA (1.2 × 10^8^ M^-1^ s^-1^). Error bars are the standard error values of three tests (n = 3).

**
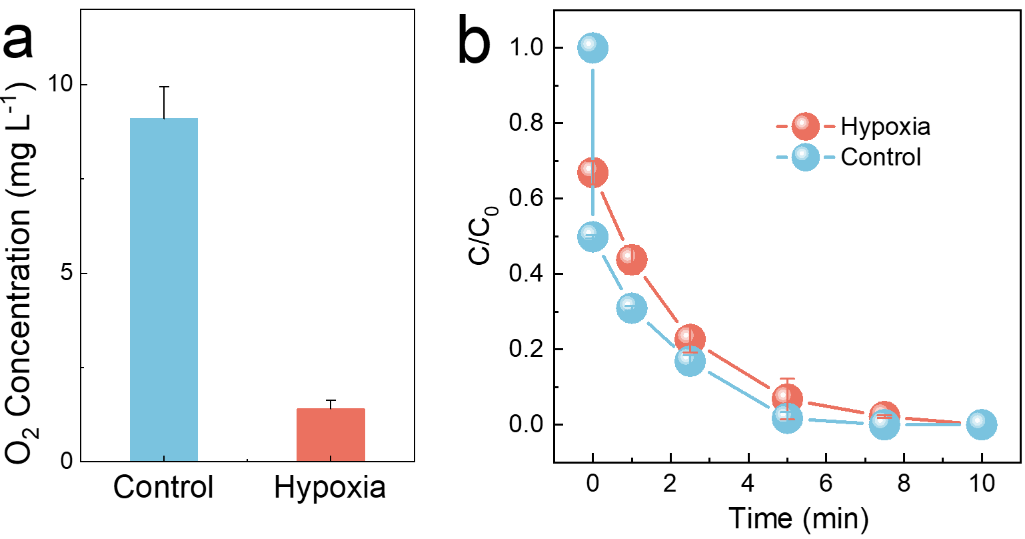
**

**Figure S36.** (a) The concentration of O_2_ in the Fe_d0.43_SA/PMS system. (b) Effects of dissolved O_2_ for PE removal in Fe_d0.43_SA/PMS system. Experimental conditions: [catalyst]_0_ = 100 mg L^-1^, [PMS]_0_ = 0.3 mM, and [PE]_0_ = 40 μM. Error bars are the standard error values of three tests (n = 3).

**Figure S37.** The PMS residue in the Fe_d0.43_SA/PMS system under different gas aeration. Experimental conditions: [catalyst]_0_ = 100 mg L^-1^, [PMS]_0_ = 0.3 mM, and [PE]_0_ = 40 μM. Error bars are the standard error values of three tests (n = 3).

**Figure S38.** The (a) removal rate and (b) *k*_obs_ of PE in pure MeOH solution by Fe_dx_SA/PMS. Experimental conditions: [catalyst]_0_ = 100 mg L^-1^, [PMS]_0_ = 0.3 mM, and [PE]_0_ = 40 μM. Error bars are the standard error values of three tests (n = 3).


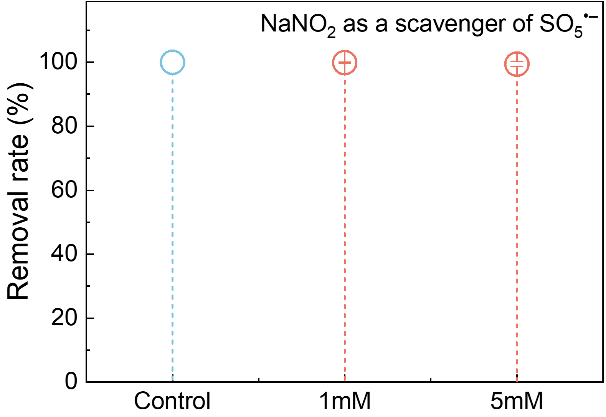


**Figure S39.** Effect of NO_2_^‒^ on the removal of PE in Fe_d0.43_SA/PMS system. Experimental conditions: [catalyst]_0_ = 100 mg L^-1^, [PMS]_0_ = 0.3 mM, and [PE]_0_ = 40 μM. Error bars are the standard error values of three tests (n = 3).


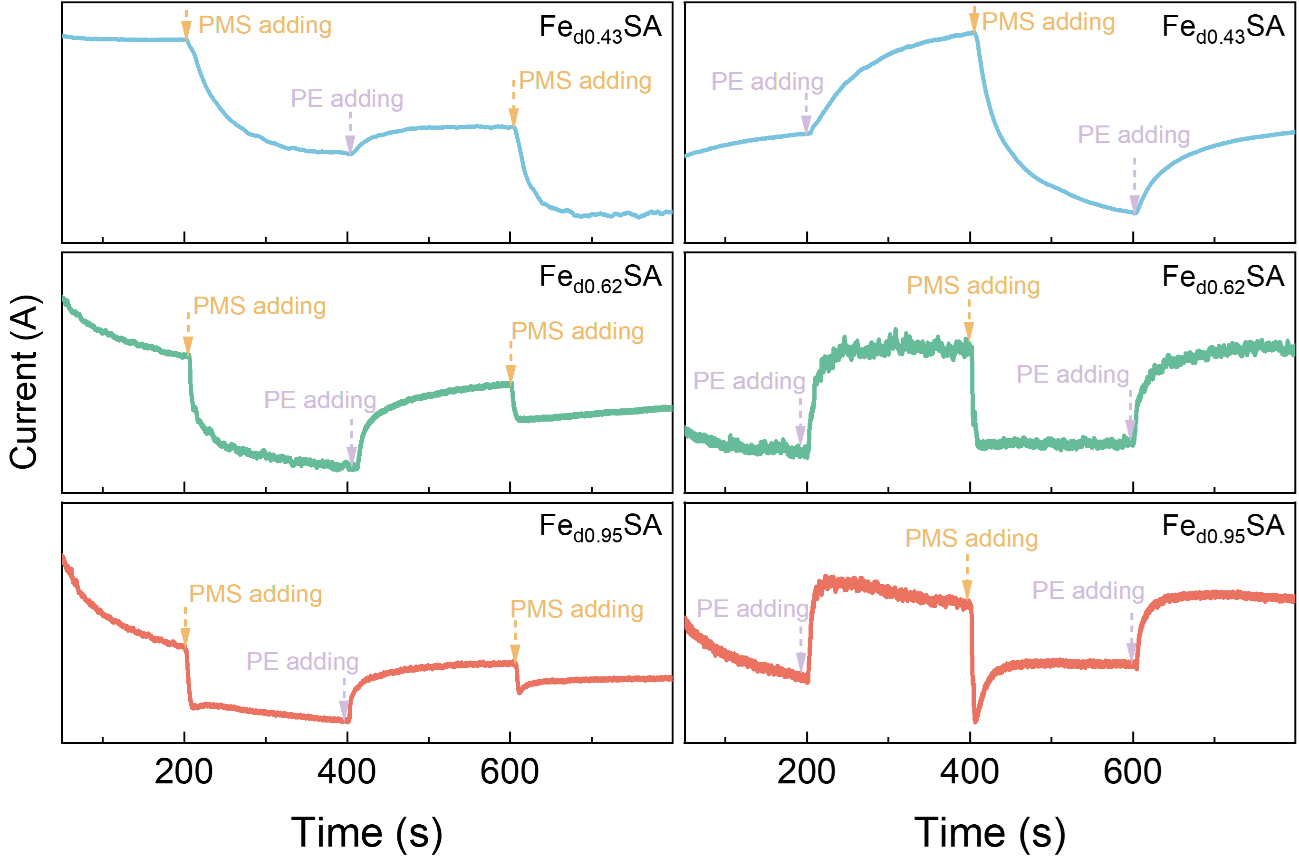


**Figure S40.** Measurements of the electron-transfer regime by chronoamperometry in different systems. Upon the addition of PMS, the current shifted sharply in the negative direction, indicating electron transfer from the catalyst to PMS. In contrast, the introduction of PE led to a positive current shift, indicating electron flow from PE to the catalyst.

**Figure S41.** The change of pH value in different initial pH conditions in the Fe_d0.43_SA/PMS system. Experimental process: pH value was regulated by H_2_SO_4_ and NaOH; firstly, PMS was dissolved in the DI water solution; secondly the pH value was altered at the corresponding value and kept at a stable value; then pH value was continuously detected in the following 10 min; when pH value kept stable for 10 min, catalyst was added into the solution and pH value was dynamically detected in the following 10 min. Experimental conditions: [catalyst]_0_ = 100 mg L^-1^ and [PMS]_0_ = 0.3 mM.


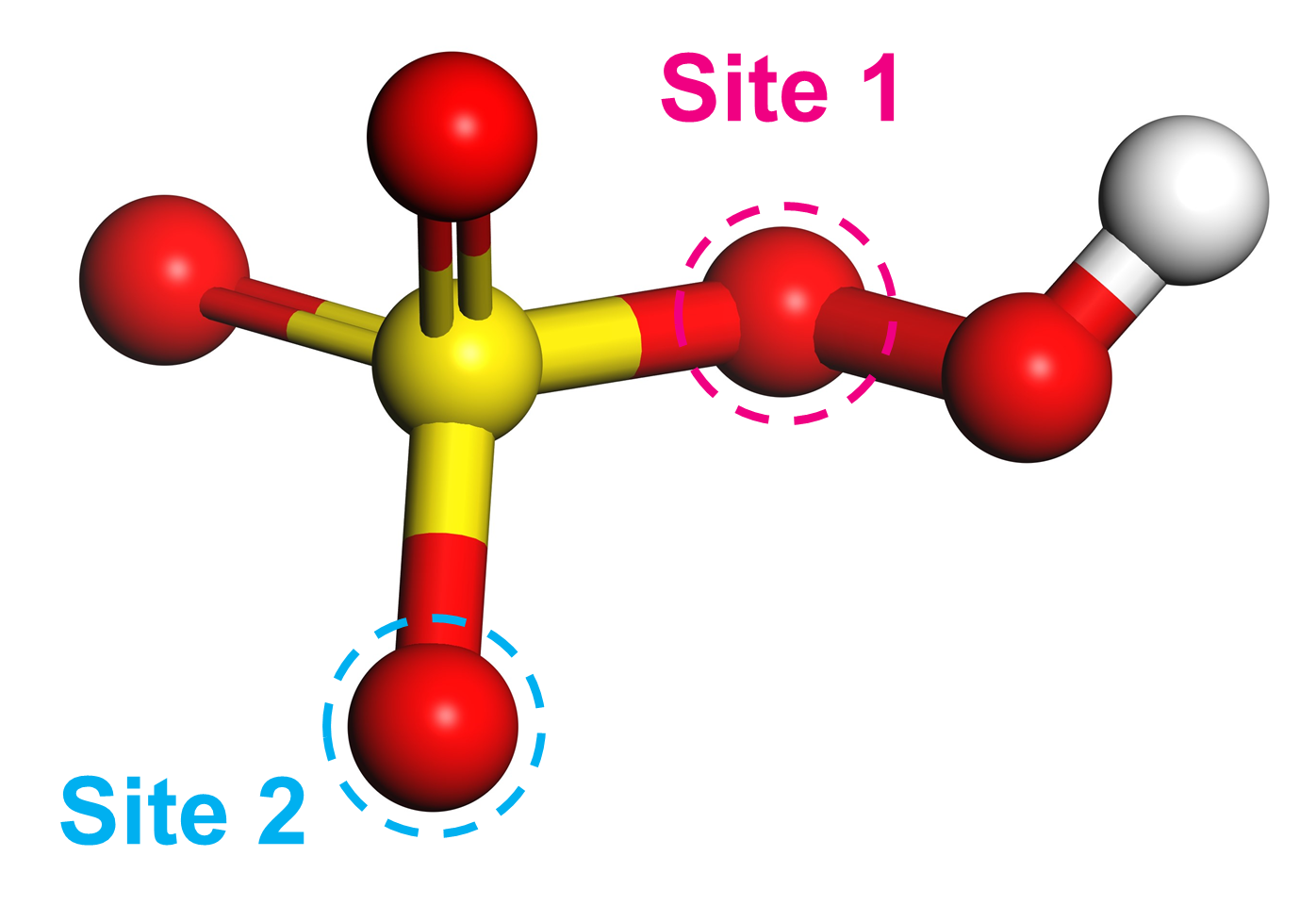


**Figure S42.** The model of PMS molecule.


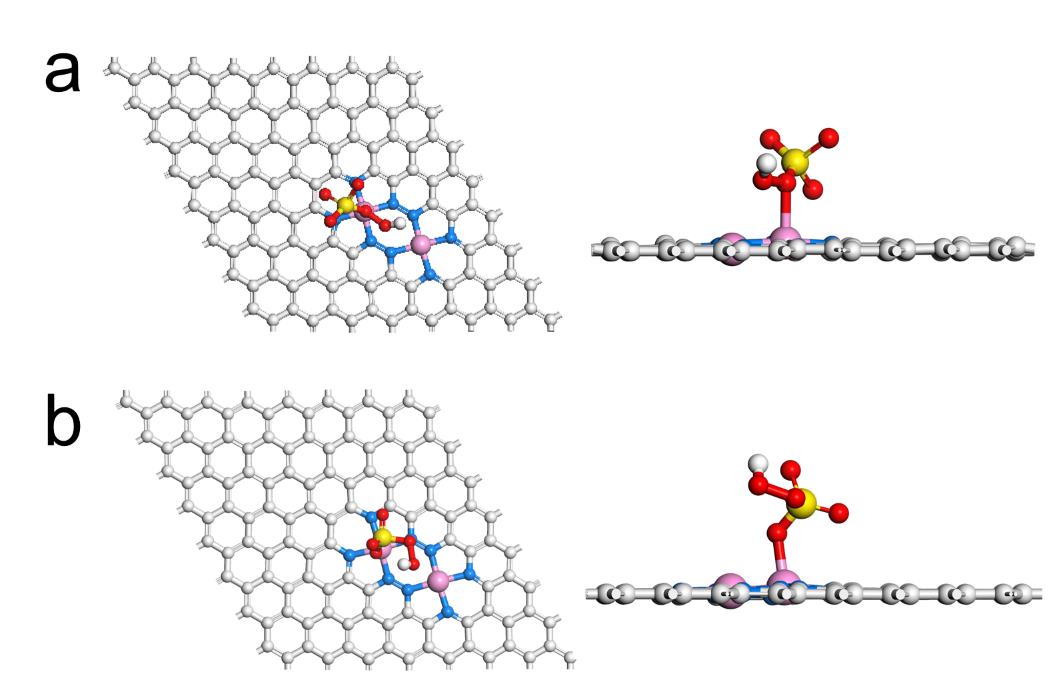


**Figure S43.** The different adsorption models of different O in PMS ((a) O site 1, (b) O site 2) on Fe_d0.43_SA catalyst.


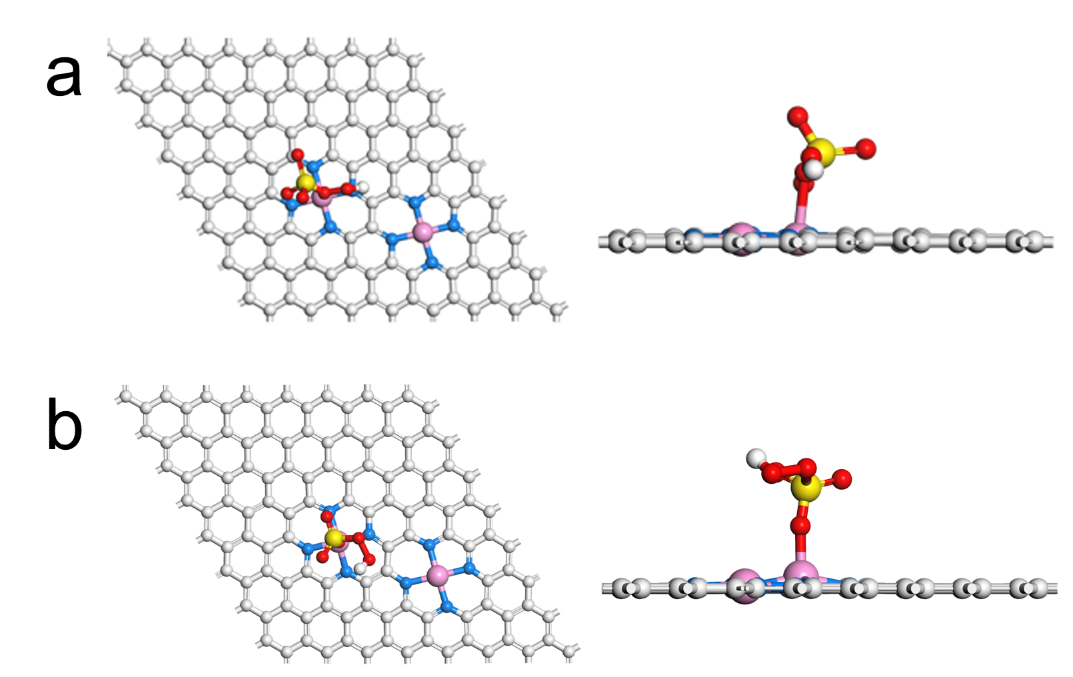


**Figure S44.** The different adsorption models of different O in PMS ((a) O site 1, (b) O site 2) on Fe_d0.62_SA catalyst.

**
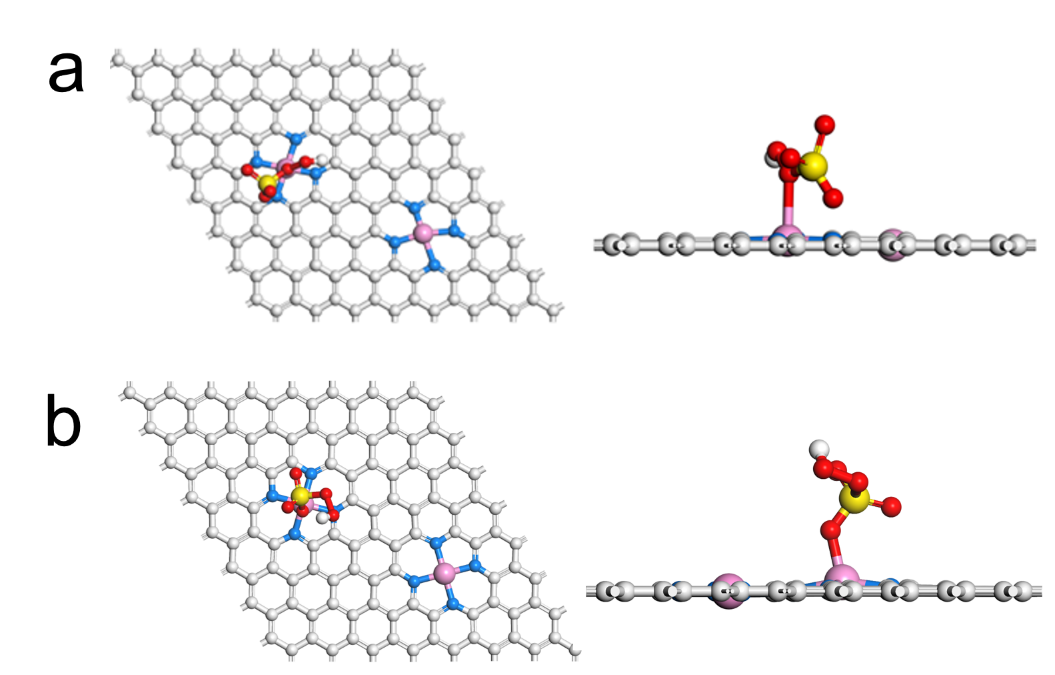
**

**Figure S45.** The different adsorption models of different O in PMS ((a) O site 1, (b) O site 2) on Fe_d0.95_SA catalyst.

**
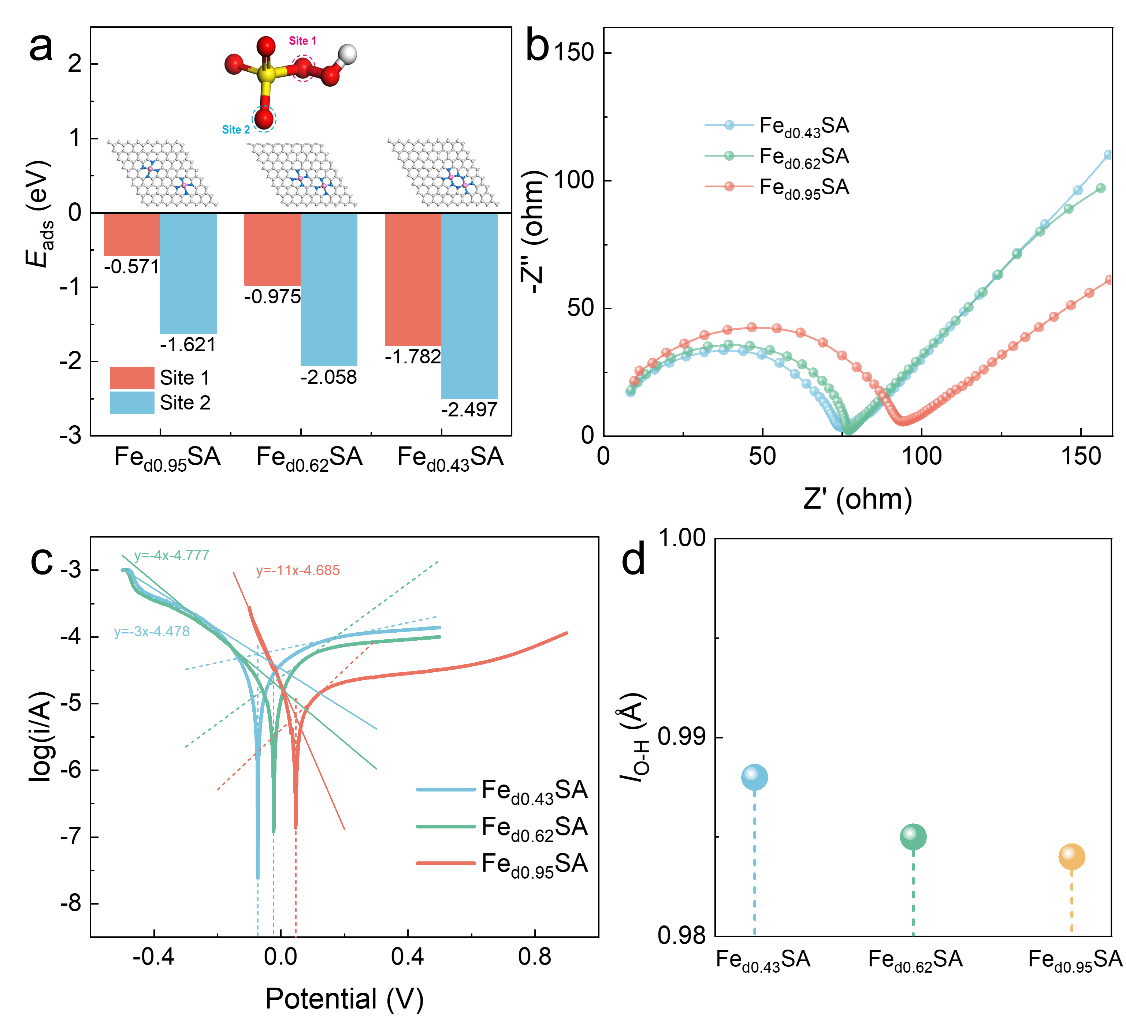
**

**Figure S46.** (a) Optimized configurations of PMS adsorbed on Fe_dx_SA and corresponding adsorption energy. (b) Electrochemical impedance spectra. (c) Tafel polarization diagram of Fe_dx_SA under open-circuit conditions. (d) The comparison of *l*_O-H_ of PMS after adsorption (The initial *l*_O-H_ of PMS before adsorption is 1.007 Å).

Notes: The Nyquist plot reveals that Fe_d0.43_SA exhibits the smallest semicircular diameter in the high-frequency region, corresponding to the lowest charge transfer resistance. This result confirms that the Fe-N_4_ twin sites configuration processed the fastest interfacial electron transfer kinetics among the series. Tafel plot shows that the Fe_d0.43_SA had the lowest Tafel slope and the most negative onset potential among the samples, indicating its superior charge transfer kinetics and stronger electron-driving force for PMS activation. These results together supported the efficient electron-transfer capacity and oriented ^1^O_2_ generation with the spin-state regulation.

**
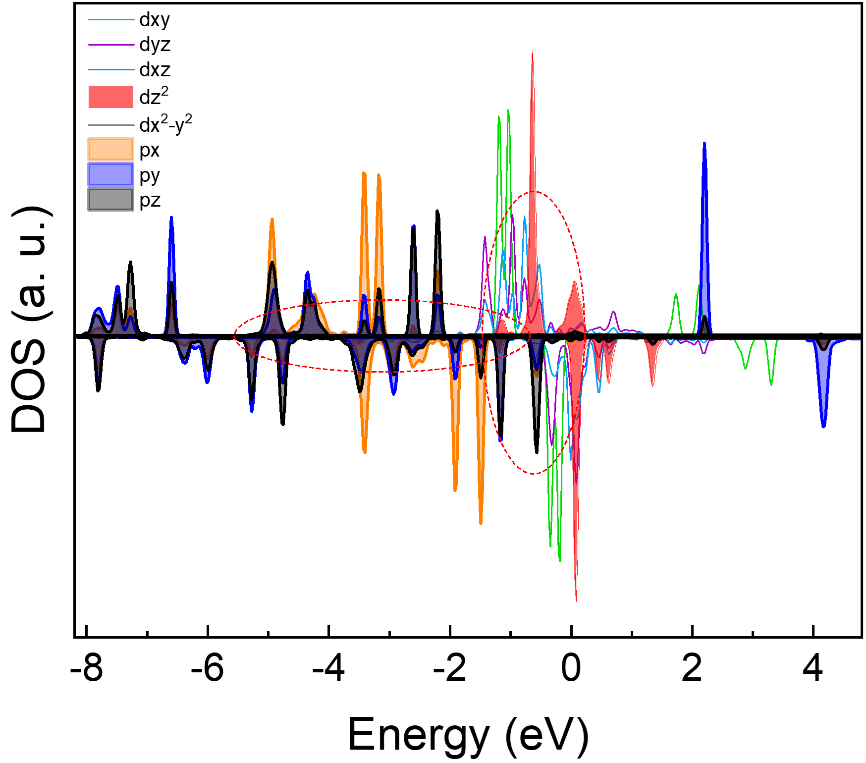
**

**Figure S47.** PDOS of Fe 3d sub-orbitals and O 2p sub-orbitals after PMS adsorbed on the Fe center. The spin-down channel of Fe 3d orbital is the predominant factor in forming *HSO_5_^‒^ with PMS. Besides, Fe dz^2^ orbitals primarily contribute to Fe-O bonding.

**
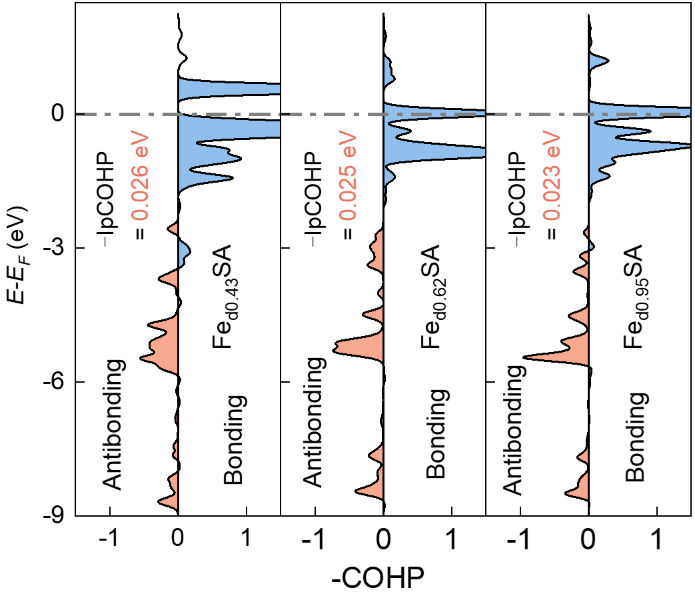
**

**Figure S48.** pCOHP between the Fe atom and the O atom in absorbed PMS.

**
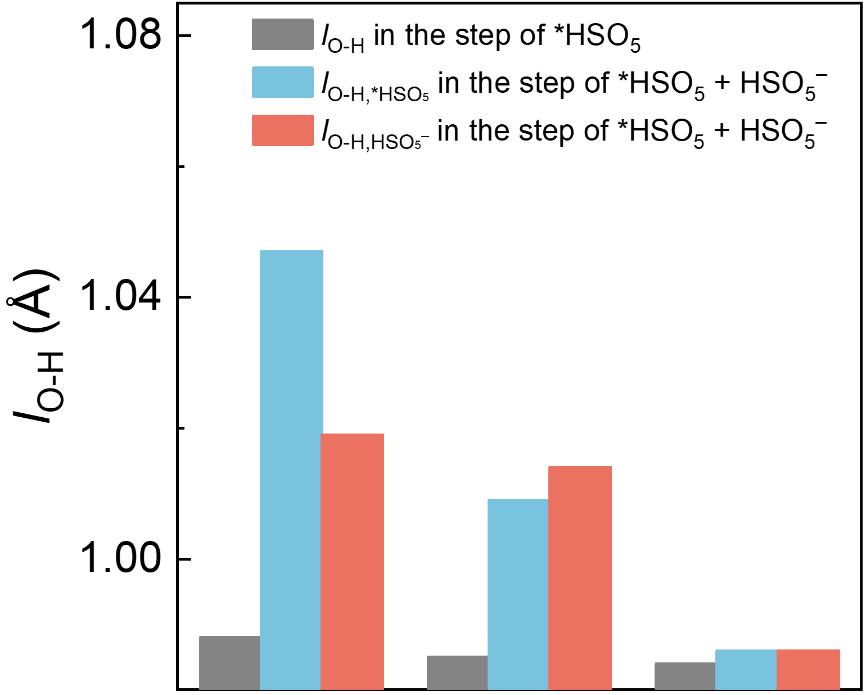
**

**Figure S49.** The comparison of *l*_O-H_ of PMS after adsorption one PMS and two PMS. The initial *l*_O-H_ of PMS before adsorption is 1.007 Å.


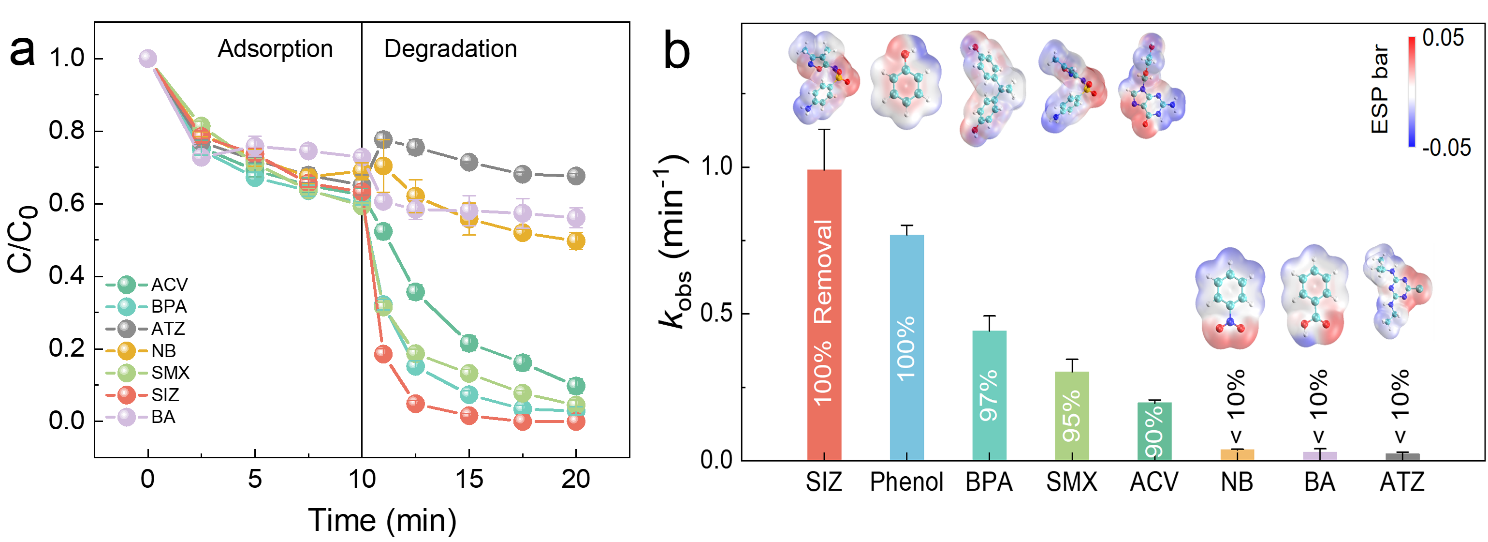


**Figure S50.** (a) The different contaminants removal rates in Fe_d0.43_SA/PMS under the same reaction conditions. (b) Removal of multiple micropollutants by Fe_d0.43_SA (inset: the corresponding ESP of the organic pollutants). Experimental conditions: [catalyst]_0_ = 100 mg L^-1^, [PMS]_0_ = 0.3 mM, and [contaminant]_0_ = 5 ppm. Error bars are the standard error values of three tests (n = 3).


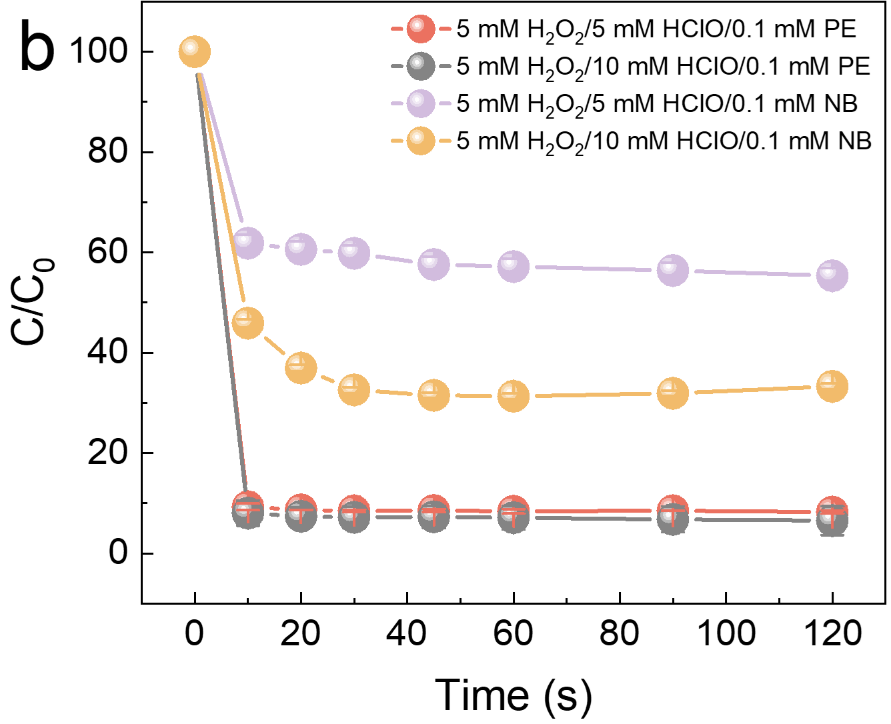


**Figure S51.** The degradation selectivity of PE and NB under different HClO/H_2_O_2_ systems. The reaction between HClO and H_2_O_2_ can rapidly generate ^1^O_2_, which is selected as a standard ^1^O_2_ system to indicate the selectivity of ^1^O_2_ for different contaminants.^[8]^ Error bars are the standard error values of three tests (n = 3).


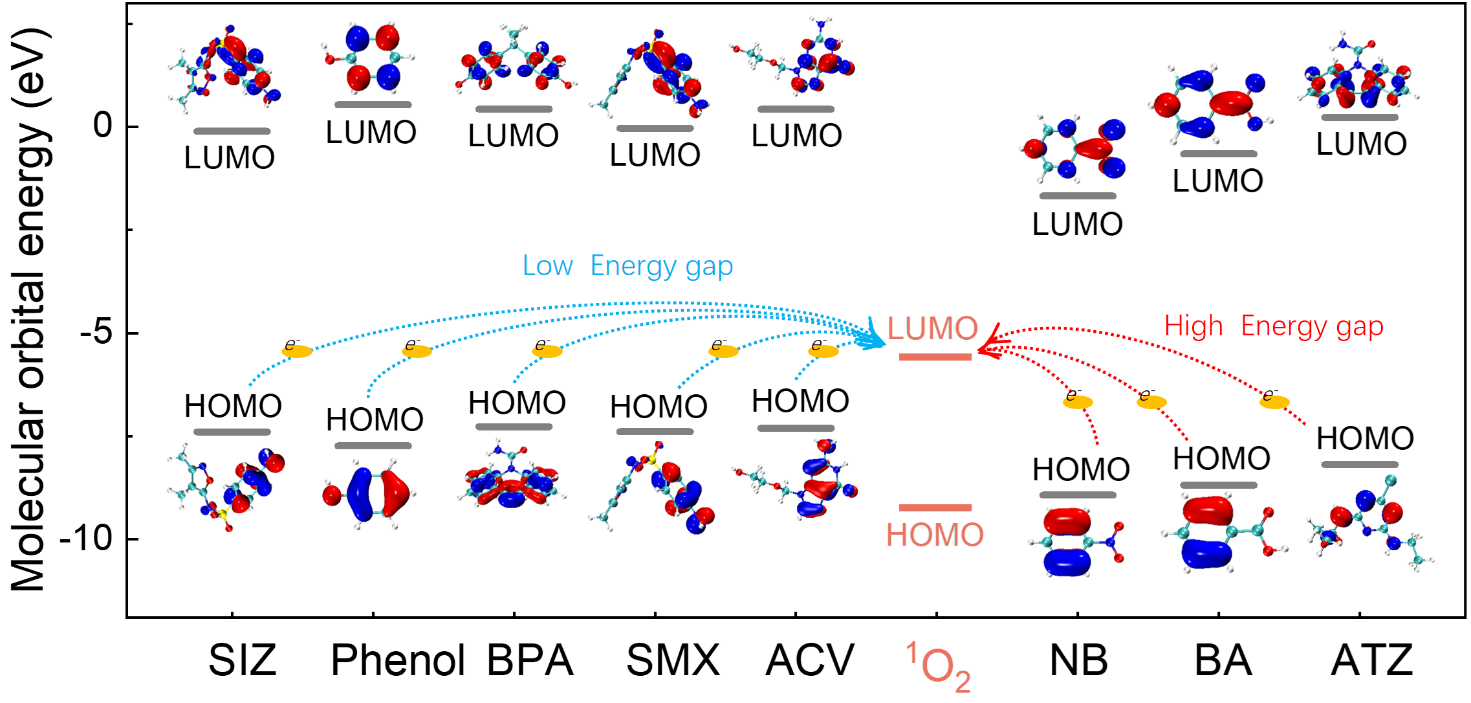


**Figure S52.** Oxidation mechanism of ^1^O_2_ for various micropollutants (inset: corresponding HOMO and LUMO of the organic pollutants).


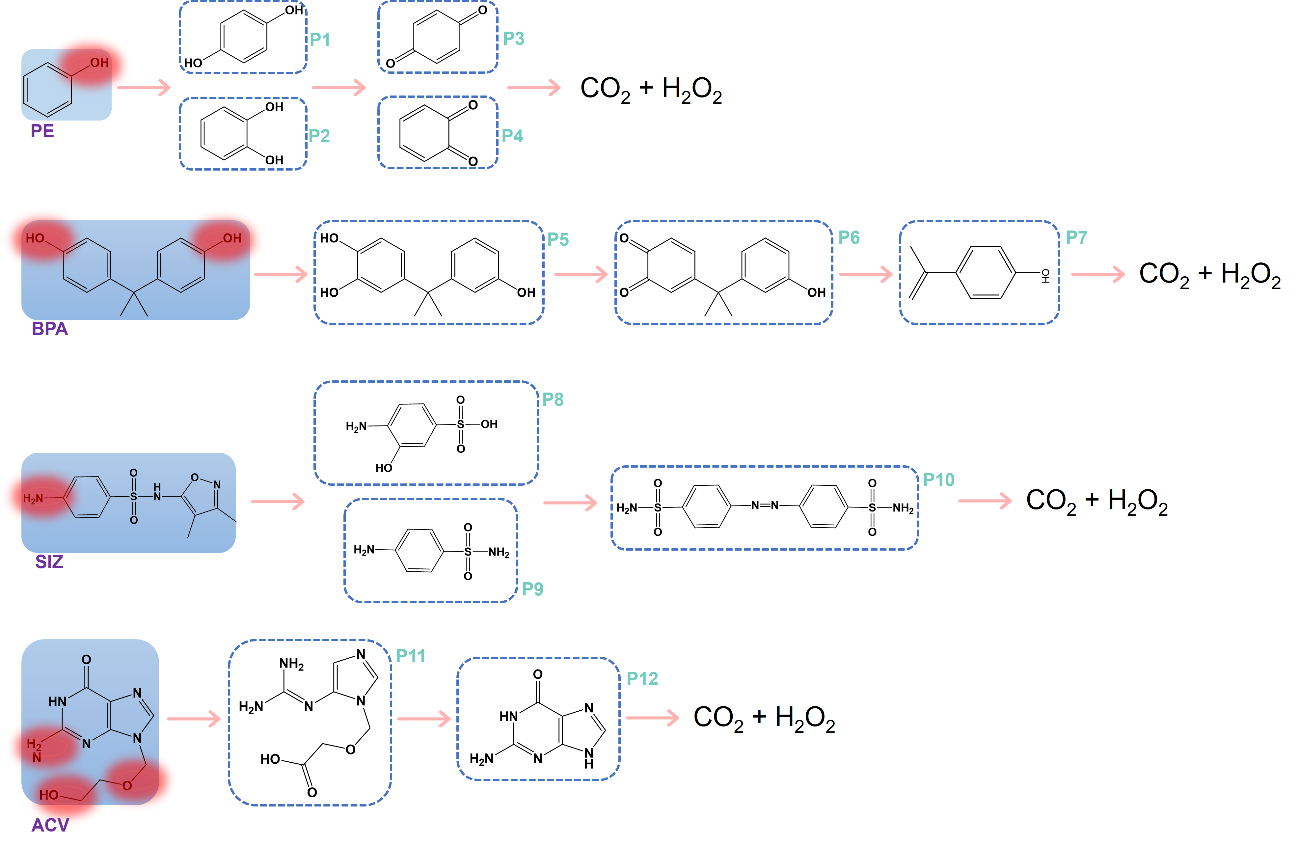


**Figure S53.** The possible degradation routes of PE, BPA, SIZ, and ACV in the Fe_d0.43_SA/PMS system. These contaminants, enriched in electron-donating groups such as phenolic hydroxyls and amino groups, were selectively oxidized by ^1^O_2_ via an initial electrophilic addition to form hydroxylated products. This reaction destabilized the molecular structure, making the intermediates more susceptible to further ^1^O_2_-driven oxidative ring-opening and ultimate mineralization into small, environmentally benign molecules.


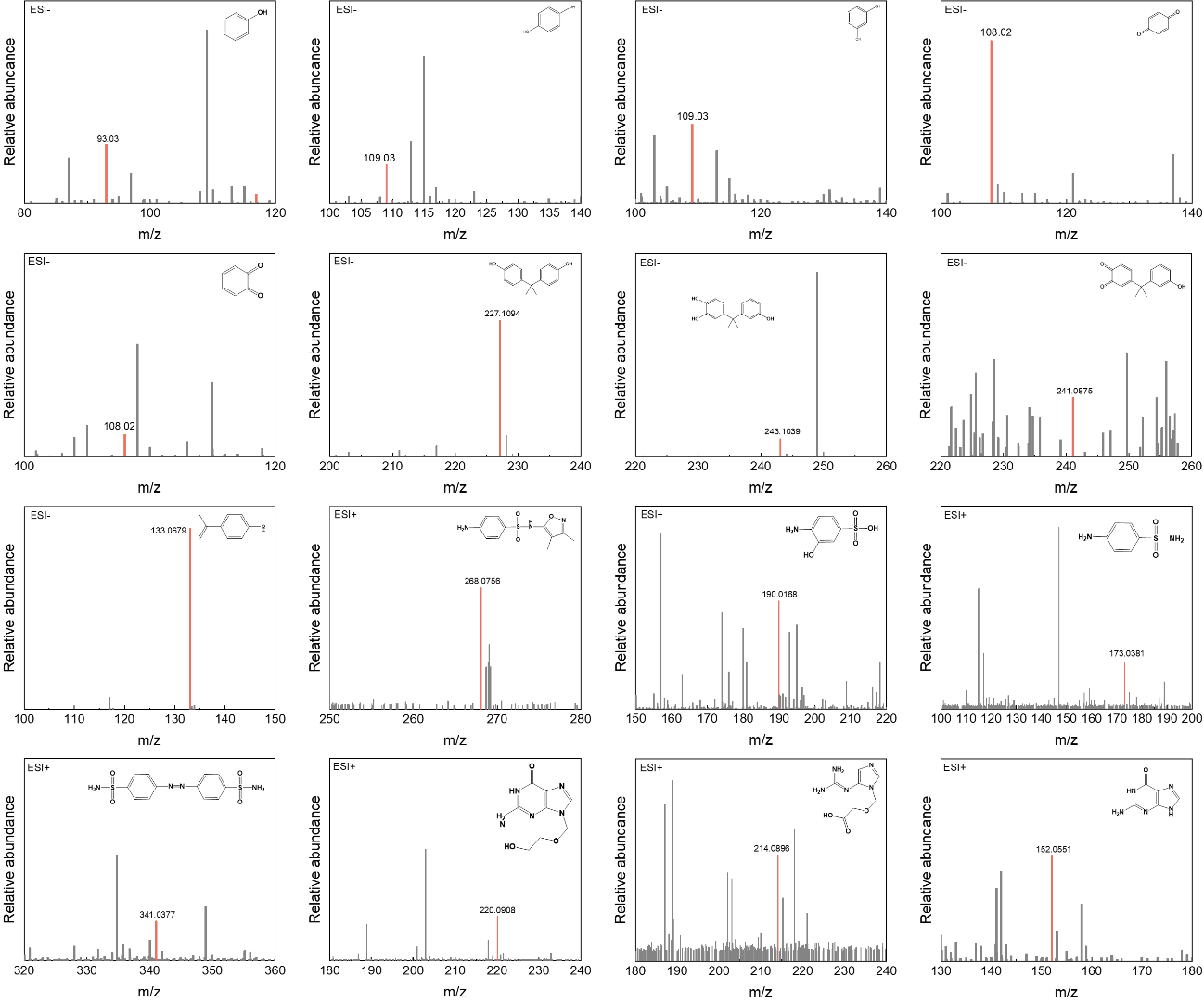


**Figure S54.** The UPLC-QTOF-MS/MS spectra of intermediates in the oxidation process for different contaminants.

**Figure S55.** The effects of various coexisting substances on PE degradation under different concentrations in Fe_d0.43_SA/PMS system. Experimental conditions: [catalyst]_0_ = 100 mg L^-1^, [PMS]_0_ = 0.3 mM, and [PE]_0_ = 40 μM. Error bars are standard error values of three tests (n = 3).

**
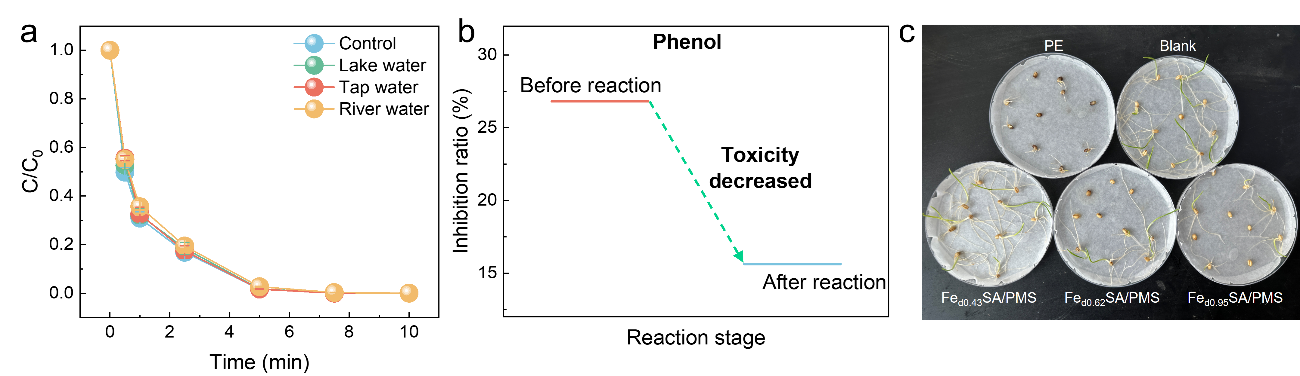
**

**Figure S56.** (a) PE degradation in different water bodies. (b) PE acute toxicity evaluation before and after the reaction. (c) Photograph of wheat seed growth in various systems. Experimental conditions: [catalyst]_0_ = 100 mg L^-1^, [PMS]_0_ = 0.3 mM, [PE]_0_ = 40 μM.

Notes: In **Figure 6**a and **Figure S56**c, PE represents that wheat seeds were treated with PE solution; Blank represents that wheat seeds were treated with clean water; Fe_d0.43_SA, Fe_d0.62_SA, and Fe_d0.95_SA represent that wheat seeds were treated with treated PE solution via Fe_dx_SA/PMS systems.

**
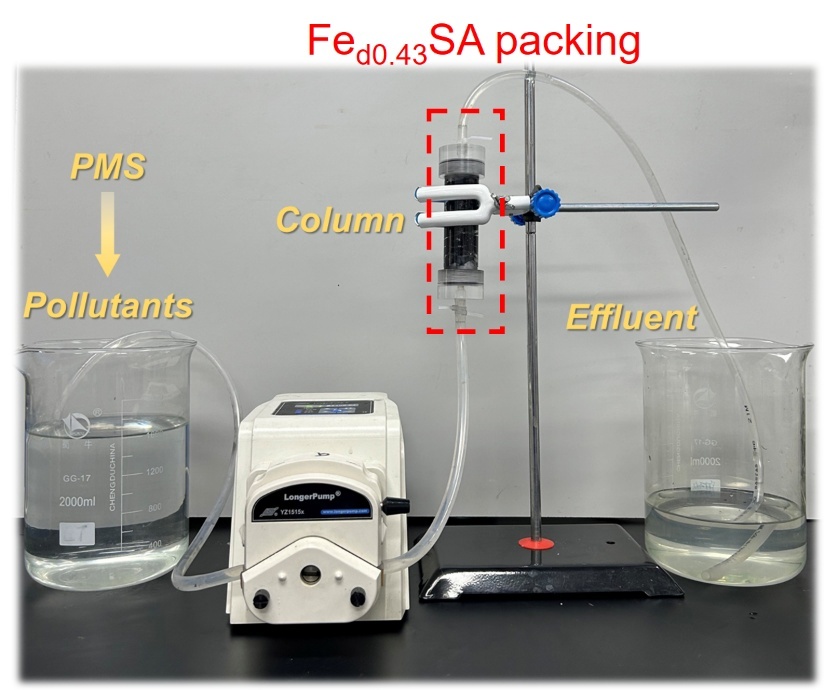
**

**Figure S57.** Photographs of the operation of the continuous-flow experiment conducted with a microreactor.

**
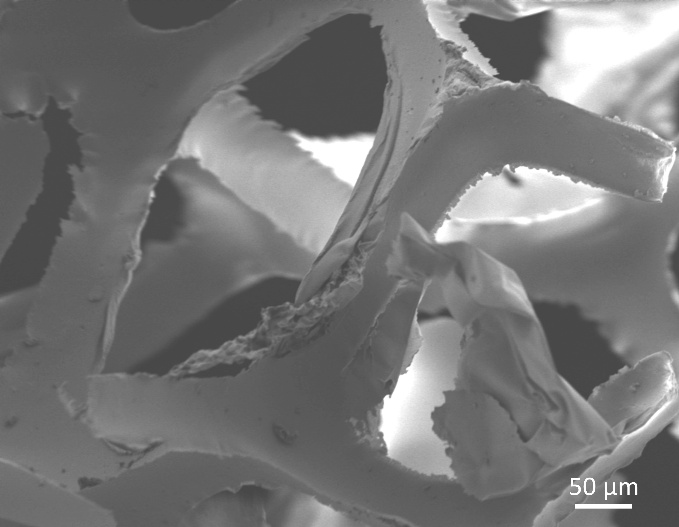
**

**Figure S58.** SEM image of cotton fibers unloaded with catalysts.

**
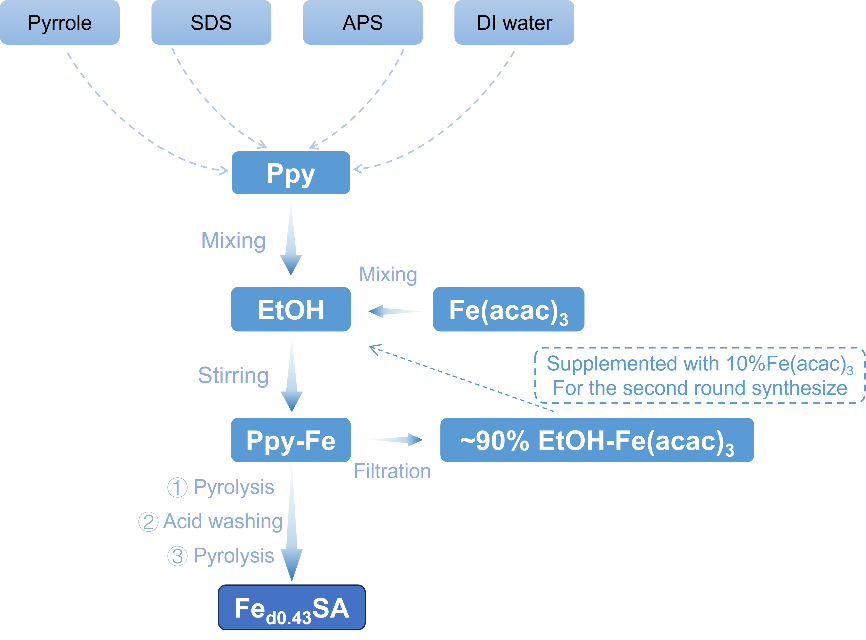
**

**Figure S59.** The flow chart of the large-scale production of catalysts.


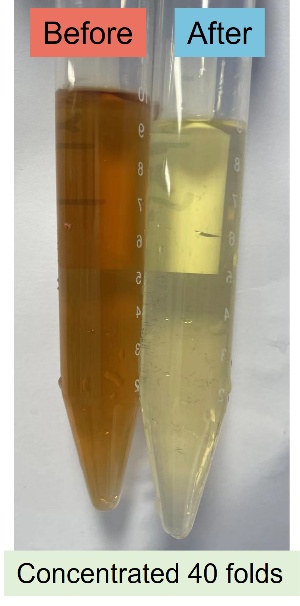


**Figure S60.** The difference in the color of the solution before and after reaction (concentrated 40-fold). Experimental conditions: [catalyst]_0_ = 100 mg L^-1^, [PMS]_0_ = 0.5 mM, stirring for 1 h.


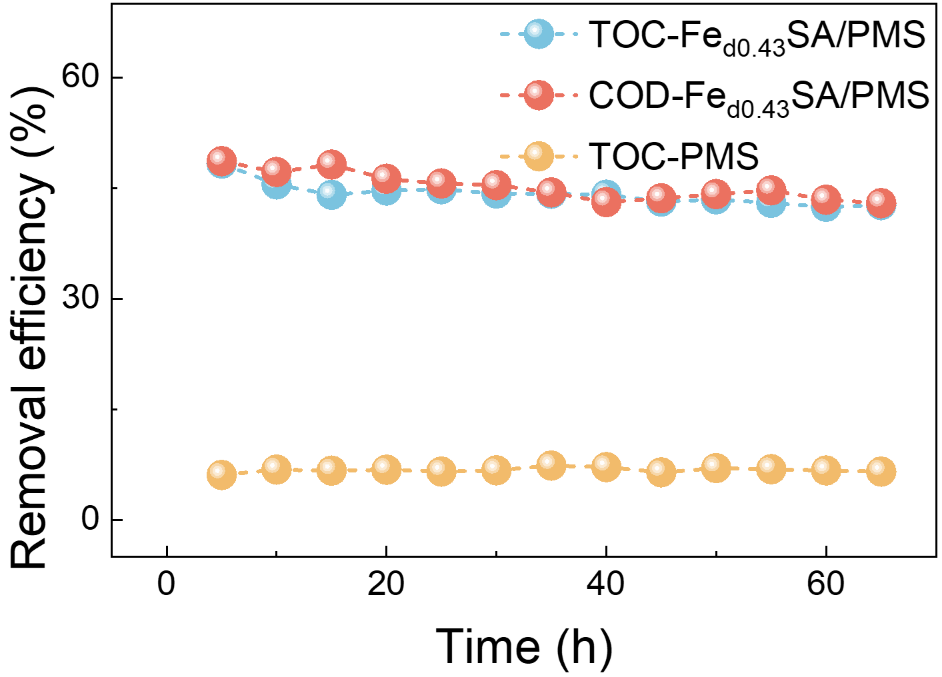


**Figure S61.** The continuous treatment of hospital wastewater in Fe_d0.43_SA/PMS with a continuous-flow mode. Experimental conditions: [catalyst]_column_ = 200 mg, [PMS]_0_ = 0.5 mM, flow rate = 80 mL h^-1^.

**Table S1.** EXAFS fitting parameters at the Fe K-edge for various samples (*Ѕ*_0_^2^=0.79).

| Sample | Shell | CN | R(Å) | σ^2^ | ΔE_0_ | R factor |
| --- | --- | --- | --- | --- | --- | --- |
| Fe foil | Fe-Fe | 8 | 2.47±0.01 | 0.0050 | 6.9±0.9 | 0.0032 |
|  | Fe-Fe | 6 | 2.85±0.01 | 0.0062 |  |  |
| Fe_d0.43_SA | Fe-N | 3.8±0.4 | 2.00±0.023 | 0.0069 | 2.58±2.82 | 0.0090 |
| Fe_d0.62_SA | Fe-N | 4.4±0.4 | 2.00±0.01 | 0.0073 | -5.2±1.7 | 0.0199 |
| Fe_d0.95_SA | Fe-N | 4.6±0.3 | 2.02±0.01 | 0.0085 | -0.1±1.6 | 0.0170 |
| FePc | Fe-N | 4.5±0.3 | 2.02±0.01 | 0.0011 | 9.1±1.9 | 0.0131 |
|  | Fe-C | 8.5±1.3 | 2.02±0.02 | 0.0026 |  |  |
|  | Fe-N1 | 2.4±1.2 | 3.01±0.04 | 0.0012 |  |  |
| Fe_2_O_3_ | Fe-O | 6.3±0.5 | 1.97±0.01 | 0.0100 | -6.7±1.6 | 0.0095 |
|  | Fe-Fe | 2.9±0.3 | 2.92±0.01 | 0.0037 |  |  |
|  | Fe-Fe1 | 8.2±1.0 | 3.37±0.01 | 0.0071 |  |  |
|  | Fe-Fe2 | 9.7±1.2 | 3.64±0.01 | 0.0012 |  |  |

*^a^N*: coordination numbers; *^b^R*: bond distance; *^c^σ*^2^: Debye-Waller factors; *^d^* Δ*E*_0_: the inner potential correction. *R* factor: goodness of fit.

The obtained XAFS data were processed in Athena (version 0.9.26) for background, pre-edge line and post-edge line calibrations. Then Fourier transformed fitting was carried out in Artemis (version 0.9.26). The k^3^ weighting, k-range of 3 - 14 Å^-1^ and R range of 1 - ~3 Å were used for the fitting of Fe foil; k-range of 3 - 11 Å^-1^ and R range of 1 - ~2 Å were used for the fitting of samples. The four parameters, coordination number, bond length, Debye-Waller factor and E_0_ shift (CN, R, ΔE_0_) were fitted without being fixed; the σ^2^ value was set.

**Table S2.** Contents of different N species for various samples.

| Sample | Content (at%) | | | | | |
| --- | --- | --- | --- | --- | --- | --- |
|  | N | Pyridinic N | Pyrrolic N | Graphitic N | Fe-N | N-Ox |
| CN | 12.2 | 42.85 | 37.55 | 12.31 | N/A | 7.29 |
| Fe_d0.43_SA | 13.33 | 41.00 | 19.29 | 21.28 | 8.59 | 9.94 |
| Fe_d0.62_SA | 10.38 | 46.05 | 10.88 | 21.42 | 13.72 | 7.94 |
| Fe_d0.95_SA | 11.38 | 42.39 | 13.97 | 21.35 | 13.59 | 5.94 |

**Table S3.** Mössbauer hyperfine parameters derived from Mössbauer spectra of Fe_dx_SA.

| Sample | Fe species | IS (mm s^-1^) | QS (mm s^-1^) | Area (%) | assignment |
| --- | --- | --- | --- | --- | --- |
| Fe_d0.43_SA | D1 | 0.20 | 0.87 | 65.00% | IS Fe(III) |
|  | D2 | 0.10 | 0.31 | 4.80% | LS Fe(III) |
|  | D3 | 0.25 | 0.34 | 30.20% | LS Fe(II) |
| Fe_d0.62_SA | D1 | 0.23 | 0.96 | 27.9% | IS Fe(III) |
|  | D2 | 0.21 | 0.51 | 46.38% | LS Fe(III) |
|  | D3 | 0.31 | 0.27 | 25.82% | LS Fe(II) |
| Fe_d0.95_SA | D1 | 0.17 | 0.81 | 14.16% | IS Fe(III) |
|  | D2 | 0.09 | 0.23 | 32.60% | LS Fe(III) |
|  | D3 | 0.18 | 0.26 | 52.80% | LS Fe(II) |

**Table S4.** The catalytic performance comparison of recently reported SACs/PMS systems.

| Catalysts | Pollutants | Catalyst dosage (g L^-1^) | PMS dosage  (mM) | *k*_obs_  (min^−1^) | *k*-value^#^  (min^−1^·M^-1^) | Ref. |
| --- | --- | --- | --- | --- | --- | --- |
| Fe_d0.43_SA | SIZ | 0.1 | 0.3 | 0.99 | 3300 | This work |
| Fe_d0.43_SA | PE | 0.1 | 0.3 | 0.76 | 2533 | This work |
| Fe_d0.43_SA | BPA | 0.1 | 0.3 | 0.44 | 1466 | This work |
| Fe_d0.43_SA | SMX | 0.1 | 0.3 | 0.30 | 1000 | This work |
| Co-N_3_ | SIZ | 0.2 | 1.85 | 0.16 | 86.4 | ^9^ |
| Co_SA_-NC | SIZ | 0.15 | 0.3 | 0.248 | 827 | ^10^ |
| ISA-Fe/MC | PE | 0.05 | 1.63 | 1.096 | 673 | ^11^ |
| CoSAC-NG | BPA | 0.005 | 1.0 | 0.0442 | 44.2 | ^12^ |
| Fe-N-C | BPA | 0.075 | 0.6 | 0.395 | 606 | ^13^ |
| SACu@NBC | BPA | 0.1 | 1.3 | 0.156 | 120 | ^14^ |
| SAFe-MCN | SMX | 0.05 | 0.5 | 0.201 | 402 | ^15^ |
| Fe_UAC_@Fe_SA_-NC | SMX | 0.15 | 1.3 | 1.13 | 868 | ^16^ |
| Fe-N-O-GC-350 | SMX | 0.1 | 0.4 | 0.24 | 600 | ^17^ |
| SA-Cu/rGO | SMX | 0.1 | 1.3 | 0.0876 | 67 | ^18^ |
| Co-C-700 | SMX | 0.1 | 0.4 | 0.43 | 1075 | ^19^ |
| CoPc/G-NH_2_ | PE | 0.02 | 0.2 | 0.226 | 1130 | ^20^ |

*^#^k*-value = *k*_obs_/PMS dosage

**Table S5.** The analysis of in situ Raman.

| Sample | ISO_4_^2‒^/IHSO_5_^‒^ | Peak position of HSO_5_^‒^ (cm^-1^) |
| --- | --- | --- |
| Fe_d0.43_SA/PMS | 3.41 | 1052 |
| Fe_d0.62_SA/PMS | 3.32 | 1053 |
| Fe_d0.95_SA/PMS | 3.28 | 1055 |
| CN/PMS | 2.85 | 1060 |
| PMS | 0.86 | 1062 |

**Table S6.** HPLC conditions for organics content analysis.

| Compounds | Ultrapure water | Methanol | Acetic acid (0.1%) | Acetonitrile | Flow rate (mL min^-1^) | Wavelength (nm) |
| --- | --- | --- | --- | --- | --- | --- |
| Sulfamethoxazole |  |  | 60% | 40% | 1.0 | 264 |
| sulfisoxazole |  |  | 60% | 40% | 1.0 | 271 |
| Phenol |  |  | 50% | 50% | 1.0 | 254 |
| Bisphenol A | 80% | 20% |  |  | 1.0 | 276 |
| Atrazine | 80% | 20% |  |  | 1.0 | 225 |
| acyclovir | 20% | 80% |  |  | 1.0 | 254 |
| benzoic acid |  |  | 50% | 50% | 1.0 | 230 |

**Table S7.** The gradient elution conditions of UPLC-Q-TOF-MS/MS

| **Time (min)** | **Mobile phase B (%)** |
| --- | --- |
| 0-2 | 2 |
| 2-12 | 2-50 |
| 12-18 | 50 |
| 18-18.5 | 50-2 |
| 18.5-21 | 2 |

**Table S8.** The calculation of the cost for pollutant treatment (1 ton).

| Material | Unit price ($ ton^-1^) | Quality required (g) | Cost for 1 ton pollutant solution (10^-3^ $) |
| --- | --- | --- | --- |
| Pyrrole | 1150 | 13.910 | 16 |
| SDS | 2990 | 20.068 | 60 |
| APS | 3250 | 51.900 | 169 |
| Fe(acac)_3_ | 6000 | 4.844 | 30 |
| H_2_SO_4_ | 150 | 169.540 | 25 |
| DI water | 30 | 692.000 | 20 |
| PMS | 1340 | 46.050 | 61 |
| **Total cost** | | | **381 (~0.381$)** |

*According to the water purification capacity of 96 L g^-1^catalyst, 1 ton of pollutant solution (40 mM phenol) needed 10.4 g of catalyst. The flow chart for the large-scale production of the catalyst is shown in Figure S59, where some resources can be recycled for the next production round. The unit prices of these chemicals are based on the average prices on the website (www.made-in-china.com, June. 2025). The data is calculated excluding the cost and mass of packaging materials.

**References**

[1] G. Kresse, Furthmuller J. Efficient Iterative Schemes for AbInitio Total-Energy Calculations Using A Plane-Wave Basis Set, *Phys. Rev. B Condens Matter* **1996**, *54*, 11169-11186.

[2] G. Kresse, Furthmüller J. Efficiency of Ab-initio Total Energy Calculations for Metals and Semiconductors Using A Plane-Wave Basis Set, *Comput. Mater. Sci.* **1996**, *6*, 15-50.

[3] J. Perdew, Burke K, Ernzerhof M. Generalized Gradient Approximation Made Simple, *Phys. Rev. Lett.* **1996**, *77*, 3865-3868.

[4] P. Blochl. Projector Augmented-Wave Method, *Phys. Rev. B Condens Matter* **1994**, *50*, 17953-17979.

[5] G. Kresse, Ab Initio Molecular Dynamics for Liquid Metals, *J. Non-Cryst. Solids* **1995**, *192-193*, 222-229.

[6] G. Kresse, J. Hafner, Ab Initio Molecular-Dynamics Simulation of the Liquid-Metal-Amorphous-Semiconductor Transition in Germanium, *Phys. Rev. B Condens Matter* **1994**, *49*, 14251-14269.

[7] G. Kresse, D. Joubert, From Ultrasoft Pseudopotentials to the Projector Augmented-Wave Method, *Phys. Rev. B* **1999**, *59*, 1758-1775.

[8] A. Maetzke, S. Knak Jensen, Reaction Paths for Production of Singlet Oxygen from Hydrogen Peroxide and Hypochlorite, *Chem. Phys. Lett.* **2006**, *425*, 40-43.

[9] M. Qian, M. Lu, M. Yan, C. Chen, Y. Hu, Y. Li, J. Chen, X.-L. Wu, Single-atom Co-N3 sites supported on Waste Paper-Derived Active Carbon for Synergistic Adsorption and Catalytic Degradation of Antibiotics, *J. Environ. Chem. Eng.* **2023**, *11*, 109219.

[10] X. H. Wang, Z. K. Xiong, H. L. Shi, Z. L. Wu, B. K. Huang, H. Zhang, P. Zhou, Z. C. Pan, W. Liu, B. Lai, Switching the Reaction Mechanisms and Pollutant Degradation Routes through Active Center Size-Dependent Fenton-Like Catalysis, *Appl. Catal. B* **2023**, *329*, 122569.

[11] Z. Li, K. Li, S. Ma, B. Dang, Y. Li, H. Fu, J. Du, Q. Meng, Activation of Peroxymonosulfate by Iron-Biochar Composites: Comparison of Nanoscale Fe with Single-Atom Fe, *J Colloid Interface Sci* **2021**, *582*, 598-609.

[12] H. Zhao, J. Song, P. Lu, Y. Mu, Single Atom Co-Anchored Nitrogen‑Doped Graphene for Peroxymonosulfate Activation with High Selectivity of Singlet Oxygen Generation, *Chem. Eng. J.* **2023**, *456*, 141045.

[13] Z. Huang, H. Yu, L. Wang, M. Wang, X. Liu, D. Shen, S. Shen, S. Ren, T. Lin, S. Lei, Ferrocene Doped ZIF-8 Derived Fe-N-C Single Atom Catalyst to active Peroxymonosulfate for Removal of Bisphenol A, *Sep. Purif. Technol.* **2023,** *305*, 122402.

[14] J. Pan, B. Gao, P. Duan, K. Guo, M. Akram, X. Xu, Q. Yue, Y. Gao, Improving Peroxymonosulfate Activation by Copper Ion-Saturated Adsorbent-Based Single Atom Catalysts for the Degradation of Organic Contaminants: Electron-Transfer Mechanism and the Key Role of Cu Single Atoms, *J. Mater. Chem. A* **2021**, *9*, 11604-11613.

[15] G. Zhao, W. Li, H. Zhang, W. Wang, Y. Ren. Single Atom Fe-Dispersed Graphitic Carbon Nitride (g-C3N4) as A Highly Efficient Peroxymonosulfate Photocatalytic Activator for Sulfamethoxazole Degradation, *Chem. Eng. J.* **2022**, *430*, 132937.

[16] S. An, J. Yang, Q. Jin, Highly Efficient Peroxymonosulfate Activation of Single-Atom Fe Catalysts via Integration with Fe Ultrafine Atomic Clusters for the Degradation of Organic Contaminants, *Sep. Purif. Technol.* **2022**, *300*, 121910.

[17] S. Wang, L. Xu, J. Wang, Iron-Based Dual Active Site-Mediated Peroxymonosulfate Activation for the Degradation of Emerging Organic Pollutants, *Environ. Sci. Technol.* **2021**, *55*, 15412-15422.

[18] F. Chen, X. Wu, L. Yang, C. Chen, H. Lin, J. Chen, Efficient Degradation and Mineralization of Antibiotics via Heterogeneous Activation of Peroxymonosulfate by Using Graphene Supported Single-Atom Cu Catalyst, *Chem. Eng. J.* **2020**, *394*, 124904.

[19] S. Wang, J. Wang, Single Atom Cobalt Catalyst Derived from Co-Pyrolysis of Vitamin B12 and Graphitic Carbon Nitride for PMS Activation to Degrade Emerging Pollutants, *Appl. Catal. B* **2023**, *321*, 122051.

[20] B. Huang, X. Ren, J. Zhao, Z. Wu, X. Wang, X. Song, X. Li, B. Liu, Z. Xiong, B. Lai, Modulating Electronic Structure Engineering of Atomically Dispersed Cobalt Catalyst in Fenton-like Reaction for Efficient Degradation of Organic Pollutants, *Environ. Sci. Technol.* **2023**, *57*, 14071-14081.
